# Deciphering global transcriptional dynamics coordinated by gene-gene regulatory interactions using single-cell data

**DOI:** 10.1038/s44320-026-00235-4

**Published:** 2026-08-03

**Authors:** Liying Zhou, Songhao Luo, Zhiwei Huang, Zhenquan Zhang, Zihao Wang, Jiajun Zhang

**Affiliations:** 1 School of Mathematics, Sun Yat-sen University, Guangzhou, 510275 China; 2 Guangdong Province Key Laboratory of Computational Science, Sun Yat-sen University, Guangzhou, 510275 China; 3 Department of Mathematics, University of California Irvine, Irvine, CA 92627 USA; 4 School of Mathematics and Statistics, Guangdong University of Technology, Guangzhou, 510520 China

**Keywords:** Chromatin, Transcription & Genomics, Signal Transduction

## Abstract

Transcription is an inherently dynamic and stochastic process that often occurs in bursts, governed by gene–gene regulatory interactions and thereby driving cell-to-cell heterogeneity. However, a genome-wide, mechanistic understanding of how regulatory networks globally shape transcriptional bursting dynamics remains lacking. Here, we present BurstLink, an interpretable and tractable statistical-mechanistic framework that simultaneously infers coupled regulatory interactions and transcriptional bursting kinetics at the genome-wide scale from single-cell data. BurstLink introduces reweighted mutual information to quantify regulatory strength as network edge weights, while jointly inferring regulatory directionality and interaction type for each gene pair within a unified mechanistic model of transcriptional bursting. Applied to mouse embryonic fibroblasts data, BurstLink reveals several genome-wide regulatory mechanisms on transcriptional bursting: downstream target genes exhibit higher burst frequency and gene-expression variability than upstream transcription factor genes; stronger transcription factor binding affinity is associated with lower burst frequency and higher burst size of target genes. Notably, positive regulation primarily enhances the burst frequency and gene-expression variability in target genes, in contrast to negative regulation. In summary, BurstLink deciphers multiple general principles of global transcriptional dynamics, providing novel biological insights into cell fate decisions.

## Synopsis

BurstLink jointly infers regulatory interactions and transcriptional bursting kinetics across an entire gene regulatory network from single-cell data, revealing genome-wide principles of how regulation shapes bursting.

- BurstLink couples an interpretable statistical mechanistic model with a scalable optimizer, enabling fast genome-wide joint inference that explicitly accounts for technical noise and cell-size variation in single-cell data.
- At genome-wide scale, downstream target genes show higher burst frequency and expression variability than their upstream transcription factors.
- Stronger transcription-factor binding affinity is associated with lower burst frequency and larger burst size of target genes.
- Positive regulation increases the burst frequency and expression variability of target genes while constraining burst size, in contrast to negative regulation.

## Abstract (web summary)

BurstLink jointly infers regulatory interactions and transcriptional bursting kinetics across an entire gene regulatory network from single-cell data, revealing genome-wide principles of how regulation shapes bursting.

## Graphical abstract

**Figure:**
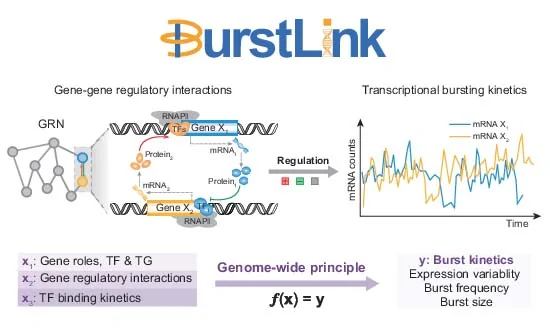

**Figure:**
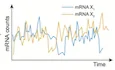

## Introduction

Cellular heterogeneity in gene expression arises from the stochastic nature of biochemical processes, playing a pivotal role in cell fate decisions, development, and human complex diseases (Desai et al, 2021; Faure et al, 2017). Transcriptional bursting, a prevalent phenomenon in both prokaryotes and eukaryotes, accounts for gene-expression heterogeneity from the dynamic mechanistic perspective, where mRNA production occurs in transient bursts as gene switches between inactive and active states (Eling et al, 2019; Friedrich et al, 2019; Raj and Van Oudenaarden, 2008; Tunnacliffe and Chubb, 2020). Increasingly, genome-wide studies have been revealing general principles of transcriptional bursting from different scales of gene expression, such as DNA sequence, epigenetic factors, and RNA synthesis (Chalancon et al, 2012). However, these principles are predominantly explored at the level of individual genes, despite growing evidence that the gene–gene interaction-constructed regulatory network also has been shown to play a crucial role in influencing cellular heterogeneity and transcriptional bursting (Kuzmin et al, 2018; van Duin et al, 2023). Notably, computational and inference methods for studying the global regulation of transcriptional bursting by gene regulatory networks (GRNs) are lacking.

Gene–gene regulatory interactions, as fundamental topological structures of GRNs, characterize the regulatory relationships between transcription factors (TFs) and their target genes (TGs), including both activated and repressed regulation (Karlebach and Shamir, 2008). Many studies pointed out that such interactions impact the stochastic fluctuations in gene expression, thereby affecting gene expression noise and regulating the transcriptional bursting kinetics (Schuh et al, 2020). For instance, expression noise can propagate through a gene-pair regulatory interaction loop, where the expression abundance of a TG may be altered by stochastic fluctuations in the abundance of a regulating TF, resulting in cellular heterogeneity (Chalancon et al, 2012; Pedraza and van Oudenaarden, 2005). Further, Gupta et al proposed a theoretical framework to characterize how the transcriptional bursting of a TF gene is propagated to its target genes (Gupta et al, 2022). Collectively, these studies underscore the critical role of gene-gene regulatory interactions in mediating gene expression, including transcriptional bursts. However, the links between regulatory interactions and transcriptional bursting have rarely been revealed on a genome-wide scale.

Recent advances in single-cell sequencing technologies, such as single-cell RNA sequencing (scRNA-seq), allowing for the measurement of genome-wide gene expression levels and cellular heterogeneity, provide an opportunity to infer the genome-wide transcriptional mechanisms (Badia-i-Mompel et al, 2023; Gupta et al, 2022). In the context of GRN inference, the reconstruction of putative regulatory interactions from scRNA-seq gene expression patterns has been comprehensively reviewed (Nguyen et al, 2021; Pratapa et al, 2020; Saint-Antoine and Singh, 2020). Existing approaches include information-theoretic methods, such as PIDC (Chan et al, 2017) and Phixer (Singh et al, 2019); machine-learning-based methods, such as GENIE3 (Huynh-Thu et al, 2010) and GRNBoost2 (Moerman et al, 2019); and integrative approaches, represented by SCENIC (Aibar et al, 2017; Bravo González-Blas et al, 2023). Single-cell multi-omics data further enhances the construction of directional and signed regulatory networks by integrating scRNA-seq with single-cell ATAC sequencing (scATAC-seq) data (Badia-i-Mompel et al, 2023), such as CellOracle (Kamimoto et al, 2023) and Pando (Fleck et al, 2023). For transcriptional bursting, scRNA-seq data, despite being static, inherently carries information about the dynamic gene expression processes over time, since the system’s current state reflects its prior behavior (Wang et al, 2023a; Wu and Jiang, 2021). Utilizing scRNA-seq data, some approaches infer transcriptional burst kinetic parameters, such as burst frequency (BF) and burst size (BS), under the assumption that genes are not externally regulated (Huang et al, 2026; Kim and Marioni, 2013; Larsson et al, 2019; Luo et al, 2023b; Ochiai et al, 2020; Ramsköld et al, 2024). Further approaches consider transcriptional bursting regulated by other biological processes, such as cell cycle (Sukys & Grima, 2025; Volteras et al, 2024), and 3D chromatin architecture (Trzaskoma et al, 2024). Furthermore, single-cell nascent RNA sequencing (scGRO-seq) now enables the direct measurement of burst kinetics, revealing coordinated transcriptional activity across the genome (Mahat et al, 2024). However, these existing methods typically address either gene–gene regulatory interactions or transcriptional bursting kinetics in isolation, without jointly modeling their interplay. Bridging this gap requires mathematical stochastic modeling capable of simultaneously inferring the coupled dynamics of gene-gene regulatory interactions and transcriptional bursting from single-cell data.

The mathematical stochastic model to be developed needs to satisfy two requirements. First, it should be interpretable, enabling the simultaneous capture of regulatory interactions between two genes and their transcriptional bursting kinetics. Many stochastic dynamic models, such as the genetic toggle switch circuit model (Gardner et al, 2000), describe transcriptional bursting as a Markovian switching process between inactive and active states. While these models can capture the underlying dynamics, their analysis and numerical solution, using approaches like finite-state projection (Munsky and Khammash, 2006) and stochastic simulation (Gillespie, 1976), often become computationally expensive and inefficient, especially for genome-wide inference tasks. This challenge brings forth the second requirement: the model should be tractable, making it better suited for genome-wide applications.

Statistical mechanistic models are an ideal strategy to meet both requirements, where the steady-state solution of a mechanistic model is expressed in terms of common statistical distributions. For example, the classic telegraph model (Peccoud and Ycart, 1995) has a steady-state solution that follows a Poisson–Beta distribution (Kim and Marioni, 2013), which can be computed numerically using the Gauss–Jacobi quadrature method (Hale and Townsend, 2013). Larsson et al successfully applied this statistical mechanistic model to infer transcriptional burst kinetics on a genome-wide scale for an individual gene (Larsson et al, 2019). Building on this, our previous work extended the telegraph model to include auto-regulation feedback by employing a statistical mechanistic model based on Poisson mixture distributions, revealing that auto-regulation distinctly modulates BF and BS (Luo et al, 2023a). Despite these advances, the development of statistical mechanistic models that account for the coupled dynamics of regulatory interactions and transcriptional bursting, as well as scalable inference algorithms to support genome-wide analyses, remains an urgent problem.

To address this problem, we proposed the BurstLink framework, which consists of two key modules: (1) a statistical mechanistic model that characterizes the coupled dynamics of gene regulatory interactions and transcriptional bursting in terms of a tractable statistical distribution with interpretable parameters. This model also explicitly accounts for the cell size variability and technical noise inherent to scRNA-seq data measurements (Tang et al, 2023). Computational analyses revealed that there are reasonable mappings between our statistical mechanistic model and the genetic toggle switch circuit model, reflecting similarities in both distribution landscapes and parameter spaces. (2) A scalable inference algorithm, integrated into a user-friendly Python package, which infers dynamic information including burst kinetics (BF, BS) and gene-expression variability (CV²) for individual gene and gene-gene regulatory interactions (regulation types, strengths, and visualization) across the whole GRN from scRNA-seq and scATAC-seq data. BurstLink was validated for precision and robustness on the synthetic data and implemented on several single-cell datasets (Desai et al, 2021; Kamimoto et al, 2023; Larsson et al, 2019).

Based on the inferred genome-wide results from mouse embryonic fibroblast data, we performed a series of downstream analyses. First, we identified gene roles (TFs, TGs) within the GRN and observed that TGs exhibit higher CV² and BF compared to TFs. Second, we investigated the influence of TF binding dynamics on transcriptional bursting, revealing that stronger TF binding affinity results in lower BF and higher BS. Third, we found that gene–gene regulatory interactions modulate burst kinetics differently, with positive regulations increasing gene-expression variability and BF while constraining BS, in contrast to negative regulations. Finally, applied to the scRNA-seq datasets from control and treatment groups exposed to DNA-damaging drugs, BurstLink enabled comparative analyses that uncovered the reshaping of both burst kinetics and gene regulation architecture following treatment. Overall, these genome-wide analyses provide new insights into the global interplay between gene regulatory interactions and transcriptional bursting.

## Results

### Overview of BurstLink

**Figure 1:**
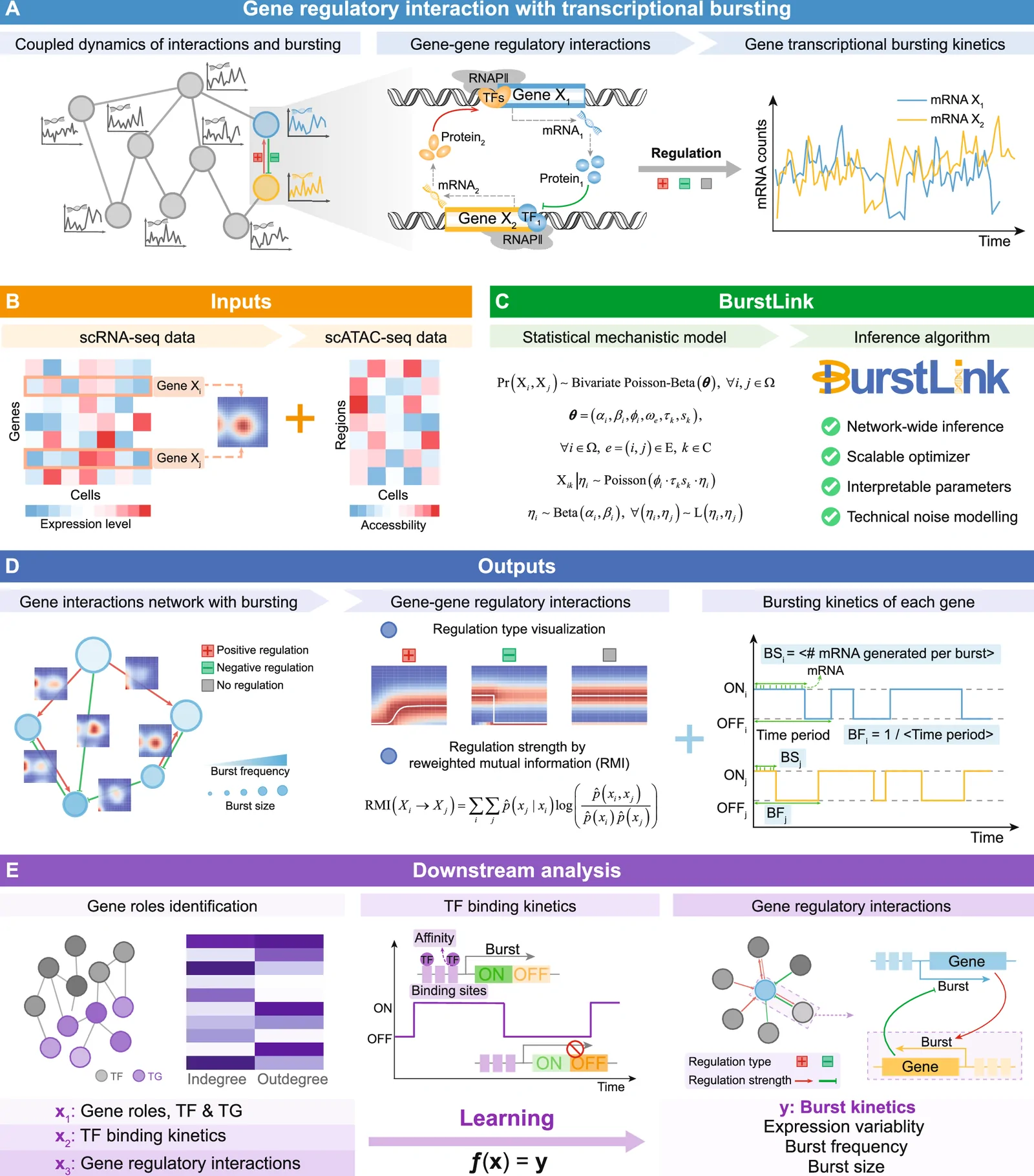
Overview of the BurstLink framework. (**A**) Schematic for the coupled dynamic system of gene regulatory interactions and transcriptional bursting kinetics. (**B**) The primary input to BurstLink is a large collection of 2D scRNA-seq count matrices, each corresponding to a pair of genes (left panel). In addition, scATAC-seq data can be incorporated to assist in screening candidate gene pairs from the scRNA-seq data (right panel). (**C**) The inference of gene regulatory interactions and bursting is based on the statistical mechanistic model, which is represented by a bivariate Poisson–Beta distribution. (**D**) Outputs of the BurstLink is gene interactions networks with bursting kinetics: (1) the inferred dynamic regulation information, including regulation visualization, regulation types, and regulation strengths measured by RMI for two directions; and (2) the inferred burst kinetics parameters, including expression variabilities, burst frequencies, and burst sizes of each gene. (**E**) Schematic for a series of genome-wide downstream analyses. BF burst frequency, BS burst size, TF transcription factor gene, TG target gene.

Cell-to-cell heterogeneity in gene expression primarily arises from transcriptional bursting, which is subject to complex gene regulatory interactions (Eldar and Elowitz, 2010; Sanchez and Golding, 2013). A key question in understanding this phenomenon is: how do dynamic gene regulatory interactions, as the fundamental architecture of GRNs, dictate transcriptional bursting on a genome-wide scale (Fig. 1A)? Here, we specify that the regulation forms in regulatory interactions mainly consist of positive regulation (promotion), negative regulation (inhibition), and non-regulation.

To solve the problem, we developed BurstLink, which can infer the coupled dynamics of regulatory interactions and transcriptional burst kinetics from single-cell data. The input of BurstLink is the set of two-dimensional joint mRNA count distributions for all gene pairs from scRNA-seq data (Fig. 1B, left). Prior chromatin accessibility information, derived from scATAC-seq or bulk ATAC-seq, is used to pre-filter these data and to provide evidence for putative direct gene–gene interactions. This pre-screening step identifies candidate regulators and substantially reduces computational cost by constraining the number of gene pairs subjected to inference (Fig. 1B, right). The output of BurstLink is the global gene regulatory interaction network incorporating bursting kinetics. For each gene pair, the inferred results are categorized into two parts (Fig. 1D): (1) the dynamic features of the regulatory interactions, including regulation types (positive, negative, and nonregulation) and regulation strengths measured by reweighted mutual information (RMI), which can be visualized by rescaled conditional probability; and (2) the dynamic parameters of the burst kinetics, including BF, BS and CV^2^ for each gene (see “Methods”).

BurstLink models each gene–gene pair, the minimal unit of a GRN, with an interpretable and tractable statistical mechanistic model based on a bivariate Poisson–Beta distribution (Fig. 1C), corresponding to the steady-state solution of the classic telegraph model widely used to describe transcriptional bursting kinetics (Vu et al, 2016). It is worth noting that the parameters of the model, ${\alpha }_{i},{\beta }_{i},{\phi }_{i}$, correspond to the activation rate, deactivation rate, and transcription rate of gene i, respectively. We have proved that our statistical mechanistic model, represented by interpretable statistical distributions, can be mapped to a complex dynamic mechanistic model in the next section (see “Methods”). In addition, to account for confounding factors in the scRNA-seq data, the model explicitly incorporates technical noise and cell size through additional parameters ${\tau }_{k}$ and ${s}_{k}$ (see “Methods”). Utilizing the alternating direction method of multipliers (ADMM) and the Gauss–Jacobi quadrature, BurstLink becomes computationally tractable, enabling rapid solutions instead of simulation for the complex dynamical gene-expression model, thus facilitating fast and scalable network-wide inference (see “Methods”). Furthermore, BurstLink identifies regulation types by visualizing rescaled conditional probabilities and quantifies regulation strengths using asymmetric RMI grounded in information theory (see “Methods”).

To explore the general principles of how gene regulatory interactions influence transcriptional bursting (represented by a vector ${{{\boldsymbol{y}}}}$), we performed a series of progressively deeper downstream analyses (Fig. 1E; Appendix Fig. S1): (1) comparing transcriptional bursting kinetics across different gene roles (TF and TG, represented by a vector ${{{{\boldsymbol{x}}}}}_{1}$) within the inferred gene regulatory interaction network; (2) analyzing the influence of TF binding kinetics (represented by a vector ${{{{\boldsymbol{x}}}}}_{2}$) on transcriptional bursting kinetics; and (3) investigating the genome-wide impact of gene regulatory loop (represented by a vector ${{{{\boldsymbol{x}}}}}_{3}$) on transcriptional bursting. We described the effects of these potential factors on transcriptional bursting mathematically as ${{{\boldsymbol{y}}}}={{{\boldsymbol{f}}}}({{{{\boldsymbol{x}}}}}_{i})$ (i=1,2,3), where ${{{\boldsymbol{f}}}}$ is a vector function measuring the relationship between factors ${{{{\boldsymbol{x}}}}}_{i}$ and the transcriptional bursting vector ${{{\boldsymbol{y}}}}$.

### BurstLink provides an interpretable framework for a dynamic mechanistic model

**Figure 2:**
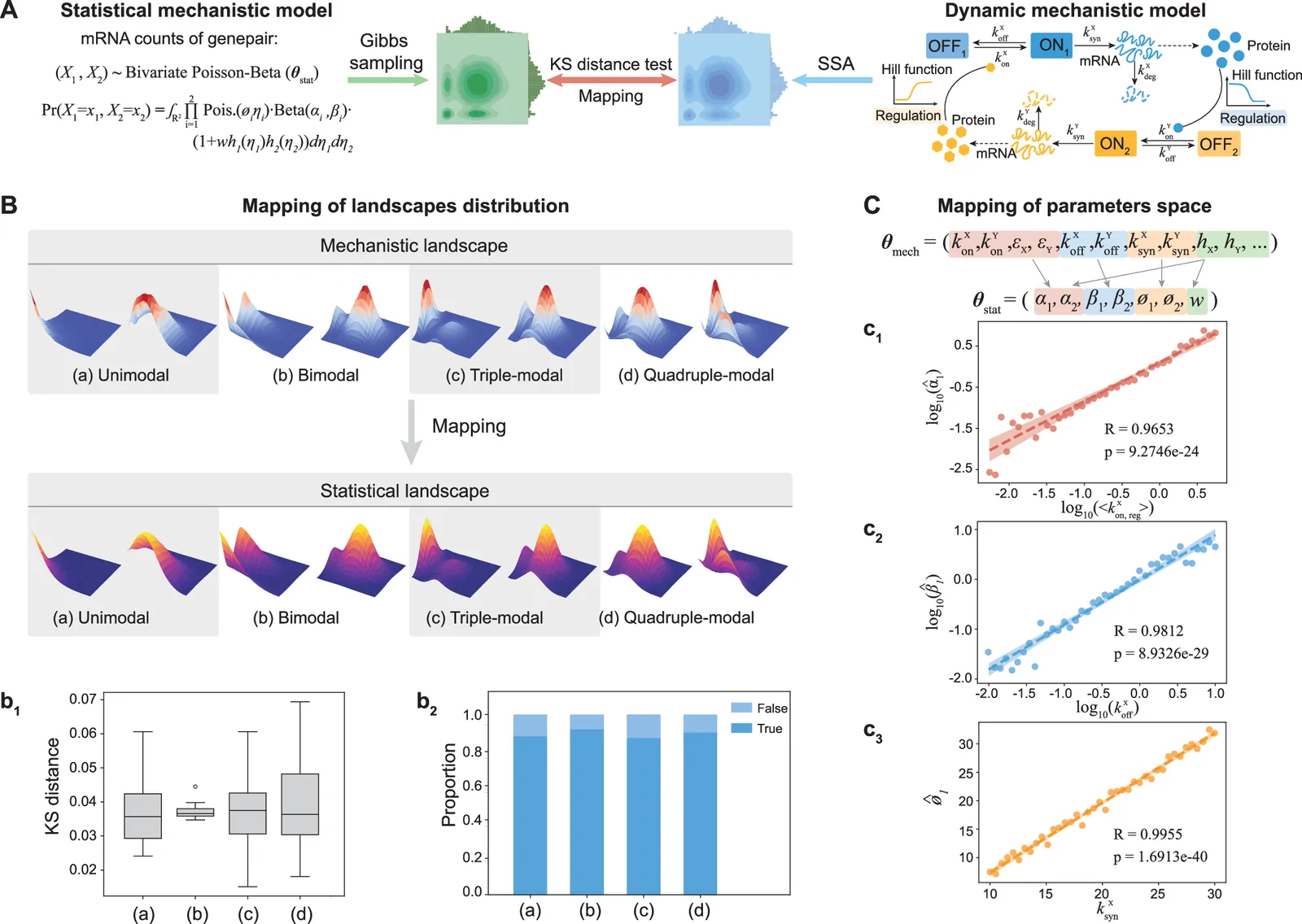
Mapping between the statistical mechanistic model and the dynamic mechanistic model. (**A**) Schematic for the mapping procedure between the statistical mechanistic model and the mechanistic model. The statistical mechanistic model is shown in the left panel. Given preset parameters, gene-pair co-expression samples are sampled from a bivariate Poisson–Beta distribution via a Gibbs sampler. The dynamic mechanistic model is shown in the right panel, where the gene encodes proteins in the ON state to regulate another gene expression as TFs via a gene–gene regulatory interaction loop. Gene-expression simulated data is generated by SSA, and a further steady distribution can be obtained. A generalized KS-test is used in the comparison between two static distributions. (**B**) Mapping results for landscape distribution between the statistical mechanistic model and the dynamic mechanistic model, such as unimodal, bimodal, triple-modal, and quadruple-modal distributions. The generalized Kolmogorov–Smirnov (KS) test results to compare the distributions of two models are shown in ${{\mbox{b}}}_{1}$, ${{\mbox{b}}}_{2}$, where dark blue (True) denotes the consistency of two distributions and light blue (False) denotes the inconsistency of two distributions. (**C**) Mapping results for the parameter space between statistical mechanistic model and dynamic mechanistic model. Scatter plots of the correspondence for ${\langle }{k}_{{{{\rm{on}}}},{{{\rm{reg}}}}}^{{\mbox{X}}}(m){\rangle}_{m}$ and ${\alpha }_{1}$, ${k}_{{{{\rm{off}}}}}^{{\mbox{X}}}$ and ${\beta }_{1}$, ${k}_{{{{\rm{syn}}}}}^{{\mbox{X}}}$ and ${\phi }_{1}$ are illustrated in ${{\mbox{c}}}_{1}$, ${{\mbox{c}}}_{2}$ and ${{\mbox{c}}}_{3}$ respectively, which are correlated in the sense of the Pearson correlation test (see the indicated values of *R* and *p* value). Detailed parameter configurations used in the simulations is provided in Appendix Table S2. Source data are available online for this figure.

In the context of the BurstLink framework, a fundamental question is whether our statistical mechanistic model (Fig. 2A, left) can effectively characterize the gene regulatory interactions and transcriptional bursting. To clarify this issue, we constructed a dynamic mechanistic model of biochemical reactions based on the genetic toggle switch circuit (Fig. 2A, right), which captures gene-pair expression kinetics (Gardner et al, 2000). This discrete stochastic model assumes the coexistence of a gene–gene regulatory loop and transcriptional bursting, where proteins encoded by two genes act as TFs to regulate each other, resulting in repressing or activating the switching process between inactive and active gene states (see “Methods”) (Tian and Burrage, 2006). To evaluate the correspondence between the statistical mechanistic model and the dynamic mechanistic model, we generated simulated data ${{{\mbox{S}}}}_{{{\mbox{mech}}}}$ using a stochastic simulation algorithm (SSA) (Gillespie, 1976) over a sufficiently large-scale parameter range (${\theta }_{{{\mbox{mech}}}}$; see “Methods”). These simulated data were fed into the BurstLink inference pipeline to estimate the parameters of the statistical mechanistic model ${\hat{\theta }}_{{{\mbox{stat}}}}$. Given the inferred parameters ${\hat{\theta }}_{{{\mbox{stat}}}}$, we then generated samples ${{{\mbox{S}}}}_{{{\mbox{stat}}}}$ via Gibbs sampling. Finally, a generalized Kolmogorov–Smirnov (KS) test was performed to compare the data $({{{\mbox{S}}}}_{{{\mbox{mech}}}},{{{\mbox{S}}}}_{{{\mbox{stat}}}})$ from the two models (Fig. 2A).

We showed the mapping of co-expression distribution landscapes between our statistical mechanistic model and the dynamic mechanistic model. The dynamic mechanistic model generates flexible co-expression distribution landscapes, including unimodal, bimodal, triple-modal, and quadruple-modal steady-state distributions, all of which can be reconstructed by BurstLink (Fig. 2B, top; Appendix Fig. S2; Appendix Table S1). The generalized KS-test results show small KS distances and high consistency across various co-expression landscapes (Fig. 2B-b1, b_2_).

We further examined the parameter mapping between the two models. The parameters ${\alpha }_{1}$, ${\alpha }_{2}$ of the statistical mechanistic model correspond to the average OFF-to-ON switching rates ${\left\langle {k}_{{{\mbox{on}}},{{\mbox{mech}}}}^{{{\mbox{X}}}}(m)\right\rangle }_{m}$, ${\left\langle {k}_{{{\mbox{on}}},{{\mbox{mech}}}}^{{{\mbox{Y}}}}(n)\right\rangle }_{n}$ for genes ${{\mbox{X}}}$ and ${{\mbox{Y}}}$ with n and m mRNA transcripts, respectively. As regulated by binding and unbinding of TFs, the OFF-to-ON switching rates ${k}_{{{\mbox{on}}},{{\mbox{reg}}}}^{{{\mbox{X}}}}(\cdot )$ and ${k}_{{{\mbox{on}}},{{\mbox{reg}}}}^{{{\mbox{Y}}}}(\cdot )$ are dynamic, incorporating the basic switching rates ${k}_{{{\mbox{bas}}}}^{{{\mbox{X}}}},{k}_{{{\mbox{bas}}}}^{{{\mbox{Y}}}}$ and regulatory parameters ${h}_{{{\mbox{X}}}},{h}_{{{\mbox{Y}}}},{r}_{{{\mbox{X}}}},{r}_{{{\mbox{Y}}}},{K}_{{{\mbox{X}}}},{K}_{{{\mbox{Y}}}}$ by the Hill function (Fig. 2C). Here, ${\langle}{k}_{{{{\mbox{on}}}},{{{\mbox{mech}}}}}^{{{\mbox{X}}}}(m){\rangle}_{m}$ and ${\langle}{k}_{{{{\mbox{on}}}},{{{\mbox{mech}}}}}^{{{\mbox{Y}}}}(n){\rangle}_{n}$ are computed at the stationary state. While varying the average OFF-to-ON switching rates over a wide range and fixing other mechanistic parameters (see “Methods”), there is a strong Pearson correlation between ${\alpha }_{1}$ and ${\langle}{k}_{{{{\mbox{on}}}},{{{\mbox{mech}}}}}^{{{\mbox{X}}}}(m){\rangle}_{m}$ (*R* = 0.9653, *p* value = 9.2746 × 10^−24^; Fig. 2C-c_1_). Similarly, the parameters ${\beta }_{1}$, ${\beta }_{2}$ correspond to the ON-to-OFF switching rates ${k}_{{{\mbox{off}}}}^{{{\mbox{X}}}}$, ${k}_{{{\mbox{off}}}}^{{{\mbox{Y}}}}$ with the high correlation (*R* = 0.9812, *p* value = 8.9326 × 10^−29^; Fig. 2C-c_2_). The parameters ${\phi }_{1}$, ${\phi }_{2}$ correspond to synthetic rates ${k}_{{{\mbox{syn}}}}^{{{\mbox{X}}}},{k}_{{{\mbox{syn}}}}^{{{\mbox{Y}}}}$ (*R* = 0.9955, *p* value = 1.6913 × 10^−40^; Fig. 2C-c_3_). Overall, these results demonstrate that BurstLink provides an interpretable and tractable framework of the dynamic mechanistic model, enabling the characterization of gene regulatory interactions and transcriptional bursting.

### Validation and benchmarking of BurstLink

**Figure 3:**
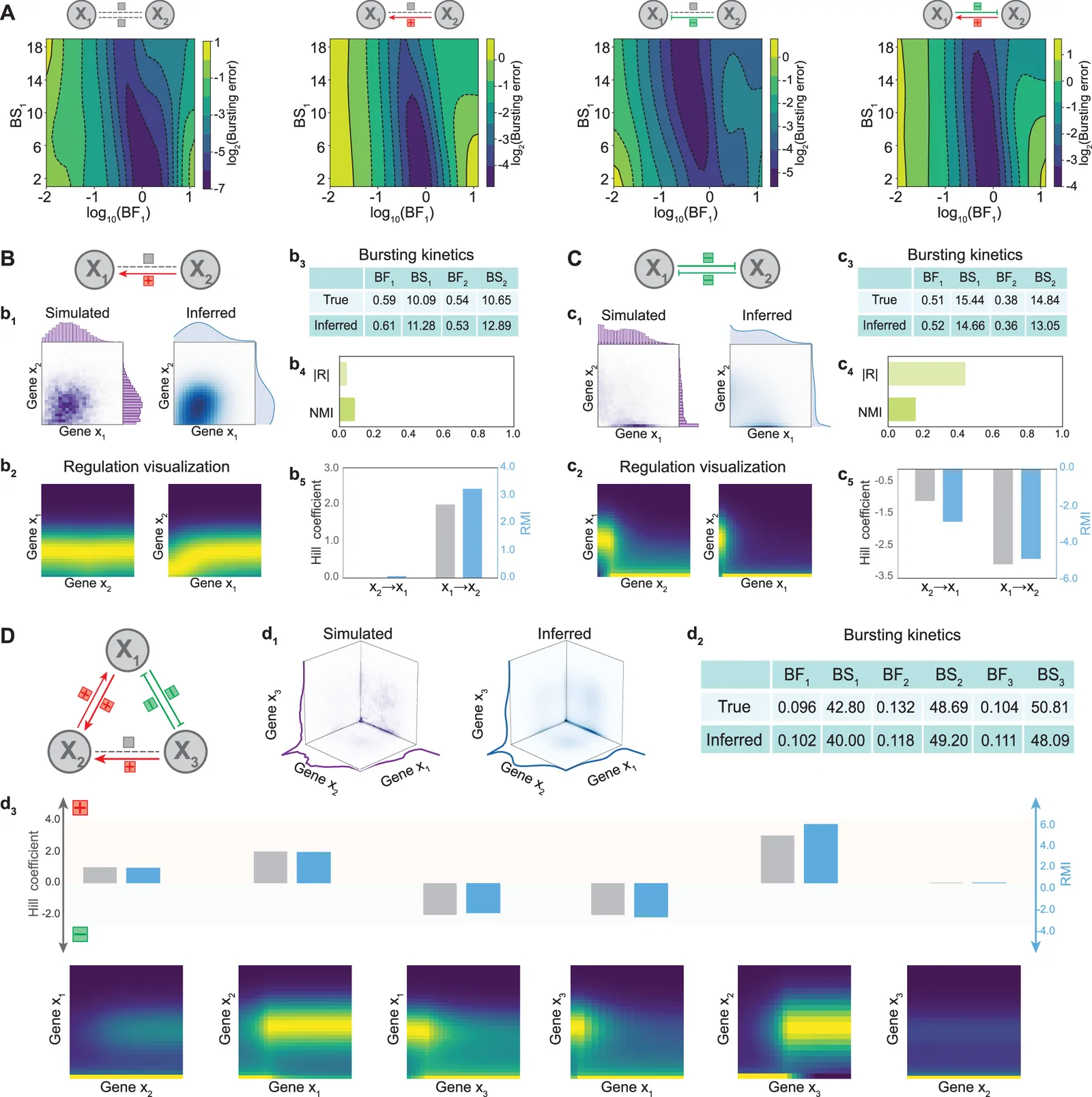
Validation on synthetic scRNA-seq data. (**A**) Contours illustrate the error of inferred burst kinetics from synthetic scRNA-seq data for four different cases. (**B**) The inferred results for synthetic scRNA-seq data under the presence or absence of regulation, including the inferred co-expression distribution (b_1_), regulatory interaction visualization (b_2_), the inferred regulation strengths by RMI (b_5_), and bursting kinetics (b_3_). The rescaled conditional probabilities are visualized as heatmaps, where the densest regions in yellow correspond to the highest rescaled conditional probability, and the sparsest regions in blue correspond to the lowest rescaled conditional probability. The trend in the densest regions indicates the regulation type (positive, negative, or nonregulation). (**C**) The inferred results for synthetic scRNA-seq data with different regulation strengths, similar to (**B**). (**D**) The inferred results for synthetic scRNA-seq data from a three-gene regulatory network, similar to (**B**). BF burst frequency, BS burst size, R Pearson correlation coefficient, NMI normalized mutual information, RMI reweighted mutual information. Source data are available online for this figure.

To evaluate the accuracy and robustness of BurstLink, we performed the inference procedure on synthetic scRNA-seq data sampling from the dynamic mechanistic model with known parameters (see “Methods”). Firstly, we assessed the precision of burst kinetics inference (BF and BS) under four different types of regulation. By varying BF and BS over a wide range and computing the estimation error, we found that the inference error remains low across a broad region of the parameter space and increases markedly only when both burst frequency and burst size are very small, indicating that adequate gene expression levels improve inference accuracy regardless of the regulation type (Fig. 3A). We also evaluated the robustness of BurstLink by varying cell number (200, 300, 500, 1000, and 2000) and dropout rate (0.1, 0.3, 0.5, 0.7, and 0.9) in the simulated samples. The accuracy and robustness of the inference increase with increasing cell number and decreasing dropout rate (Appendix Fig. S19).

Next, we assessed the validity of the inference for the regulatory interactions. In the first scenario, we considered a setup where gene ${{{\mbox{X}}}}_{2}$ is positively regulated by gene ${{{\mbox{X}}}}_{1}$ (${h}_{1}=0$, ${h}_{2}=-2$). Applying BurstLink to this synthetic data, we obtained the following inferred results: (1) the inferred joint distribution, consistent with the ground-truth joint distribution of two genes obtained from SSA (Fig. 3B-b_1_), (2) regulatory interaction visualization using the rescaled conditional probability heatmap (Fig. 3B-b_2_), and (3) the dynamic information, including regulation strengths and burst kinetic parameters (Fig. 3B-b_3_, b_5_). Specifically, the left heatmap in the regulation visualization panel of Fig. 3B-b_2_ shows that ${{{\mbox{X}}}}_{1}$ remains stable despite changes in ${{{\mbox{X}}}}_{2}$, which suggests that there is no regulation of ${{{\mbox{X}}}}_{2}$ on ${{{\mbox{X}}}}_{1}$. The right heatmap pattern illustrates that the number of ${{{\mbox{X}}}}_{2}$ increases from a low level and gradually stabilizes with the rise of ${{{\mbox{X}}}}_{1}$, demonstrating a positive regulatory effect of ${{{\mbox{X}}}}_{1}$ on ${{{\mbox{X}}}}_{2}$. These results indicate that the inferred regulation types are consistent with those set in the dynamic mechanistic model. Furthermore, RMI quantifies the regulation strength in both directions (Fig. 3B-b_5_), however, the values of traditional correlation coefficients (R) and normalized mutual information (NMI) are too small to assess these regulatory relationships (Fig. 3B-b_4_). The inferred burst kinetics (BF and BS) closely matched the values set in the dynamic mechanistic model (Fig. 3B-b_3_).

In the second scenario, we configured a regulatory setup where ${{{\mbox{X}}}}_{1}$ and ${{{\mbox{X}}}}_{2}$ negatively regulate each other with different regulation strengths (${h}_{1}=1$, ${h}_{2}=3$; Fig. 3C-c_1_). BurstLink recovered both transcriptional bursting kinetics (Fig. 3C-c_3_) and gene regulatory information from the simulated data (Fig. 3_C-c2_, c_4_, c_5_). The inferred results indicate negative regulation in both directions, and further shows that ${{{\mbox{X}}}}_{2}$ responds more rapidly and sensitively to ${{{\mbox{X}}}}_{1}$, due to a stronger regulation parameter (${h}_{2}=3$; Fig. 3C-c_2_). Moreover, RMI effectively distinguishes the relative magnitude of regulation strengths (Fig. 3C-c_5_), accurately capturing the asymmetry in the interaction. In the third scenario, we generated synthetic data with a three-gene mutual regulatory network, in which all three genes regulate one another with preset regulatory strengths (Fig. 3D). The inferred distribution recapitulates both the joint and marginal features of the simulated co-expression landscape (Fig. 3D-d_1_). The inferred regulation types and strengths align with those specified in the dynamic mechanistic model (Fig. 3D-d_3_), and the inferred burst kinetics (BF and BS) reflect the true values set in the mechanistic configuration (Fig. 3D-d_2_). Overall, BurstLink demonstrates effectiveness, robustness, and scalability in inferring the coupled dynamics of regulatory interactions and burst kinetics from synthetic data, laying a solid foundation for application to experimental scRNA-seq data analysis.

Furthermore, we performed systematic benchmarking against current GRN inference methods using scRNA-seq data from mouse embryonic fibroblasts (C57 × CAST) (Larsson et al, 2019) (see “Methods”). As summarized in Appendix Fig. S3, benchmark analyses evaluated accuracy, robustness, computational scalability, and methodological capabilities. The results demonstrate that BurstLink achieves regulatory interaction inference performance comparable to current state-of-the-art methods across a wide range of evaluation metrics and benchmark settings. Importantly, beyond competitive GRN reconstruction performance, BurstLink jointly infers transcriptional bursting dynamics and regulatory interactions within a unified mechanistic framework, providing biologically interpretable insights into both GRN structure and bursting regulation that are not readily available from existing methods.

### Genome-wide inference on single-cell multi-omics data of mouse embryonic fibroblasts

We applied BurstLink to the scRNA-seq data of mouse embryonic fibroblasts (CAST/EiJ × C57BL/6J, Data ref: Larsson et al, 2019). From the original UMI data containing 10,727 genes and 448 cells, we retained 4311 genes involving 291,228 gene pairs observed mRNA count matrices, which were screened by candidate gene pairs based on the base-GRN constructed from scATAC-seq data (Data ref: Kamimoto et al, 2023) (Appendix Fig. S4A,B; see “Methods”). We employed these data in our inference framework and eventually obtained 4173 genes with valid inferred burst kinetic parameters, varying over a wide range of gene expression means and variabilities (Appendix Fig. S4C,D).

**Figure 4:**
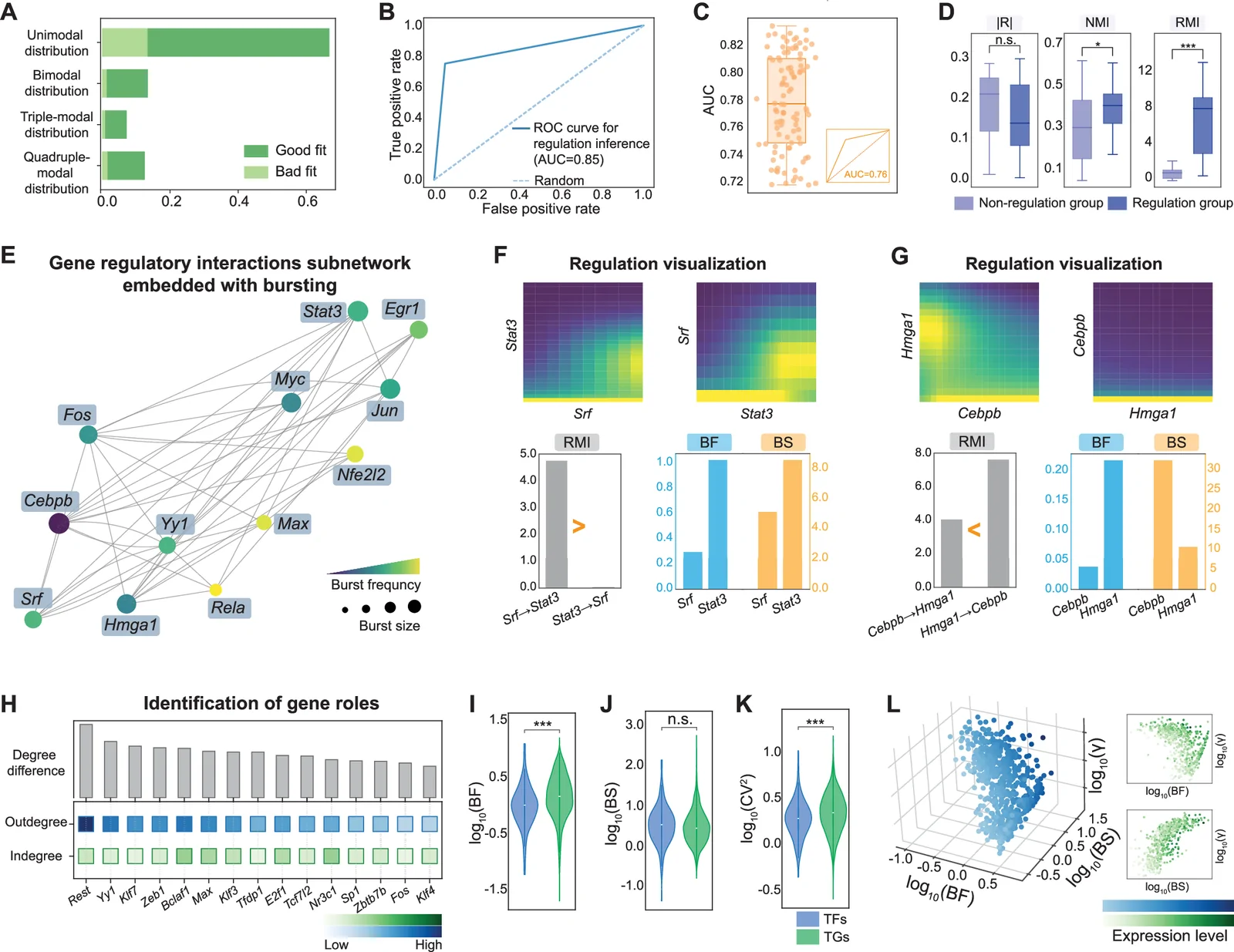
Genome-wide inference on single-cell multi-omics data of mouse embryonic fibroblasts. (**A**) Goodness-of-fit assessment for all inferred gene pairs, where the histogram displays the proportion of gene pairs exhibiting good fit (green) or poor fit (light green). (**B**) The ROC curve is used to show the accuracy of inferring the existence of regulation using the base-GRN as a label, where AUC is the area under the ROC curve. (**C**) Distribution of AUC values across 100 randomly selected fully connected subnetworks, showing overall AUCs between 0.72 and 0.82. (**D**) Box plots for R, NMI, and RMI between nonregulation and regulation groups. Statistical significance was assessed using a two-sided Welch’s *t*-test. The exact *P* values are: *P* = 0.31, 0.02, and 3.32 × 10^−10^. The sample size is n = 34. Box plots show the median (center), the 25th and 75th percentiles (box bounds), and whiskers extending to the most extreme data points within 1.5 × the interquartile range. (**E**) UMAP embedding of the inferred gene regulatory interactions subnetwork with bursting, where the size and color of points represent burst size and frequency, respectively. (**F**,** G**) Visualization of regulatory interactions for representative gene pairs (*Srf*-*Stat3* and *Cebpb*-*Hmga1*), showing RMI, BF, and BS. (**H**) Bar plot illustrating the identification of TFs and TGs based on the difference between out-degree and in-degree, shown in the upper panel, and a bubble chart displaying the corresponding out-degree and in-degree values in the lower panel. (**I**–**K**) Violin plots for ${{\mbox{CV}}}^{2}$, BF, and BS between the TFs group and TGs group. Violin plots show the data distribution. The embedded box plots show the median (center), the 25th and 75th percentiles (box bounds), and whiskers extending to the most extreme data points within 1.5 × the interquartile range. Statistical significance was assessed using a two-sided Welch’s *t*-test. The exact *P* values are: *P* = 2^.6^6 × 10^−6^, *P* = 0.33 and *P* = 2.22 × 10^−3^. The sample size is n = 4173. (**L**) 3D scatter plot showing the relationship among BF, BS, and affinity γ with point color representing gene expression level. The right panels display the corresponding pairwise 2D projections for ${\log }_{10}({\mbox{BF}})$ versus ${\log }_{10}(\gamma )$ and ${\log }_{10}({\mbox{BS}})$ versus ${\log }_{10}(\gamma )$. ROC receiver operating characteristic curve, *AUC* area under the ROC curve, R Pearson correlation coefficient, NMI normalized mutual information, RMI reweighted mutual information, TF transcription factor gene, TG target gene, BF burst frequency, BS burst size, CV^2^ gene-expression variability. Statistical significance is denoted as follows: *P < 0.05, **P < 0.01, and ***P < 0.001. Source data are available online for this figure.

We performed a series of statistical tests to validate the inferred results. Firstly, goodness-of-fit tests (see “Methods”) demonstrate that the joint distributions from the inferred parameters are consistent with those obtained from the input data across different co-expression landscapes (Fig. 4A). Additionally, the inferred mean expression and variability closely align with the original scRNA-seq data (Appendix Fig. S5A). Second, we evaluated regulatory interactions by comparing the inferred results with the base-GRN. The receiver operating characteristic (ROC) curve exhibits a high area under the ROC curve (AUC = 0.85; Fig. 4B), indicating that BurstLink can accurately identify regulatory interactions. Third, to further evaluate the inference performance of BurstLink without relying on prior regulatory information from base-GRN, we randomly constructed fully connected subnetworks composed of ten genes from scRNA-seq data and directly applied BurstLink for inference. For each subnetwork, the inferred presence or absence of regulation is compared with the ground-truth labels from scATAC-seq data to compute ROC curves. Across 100 randomly sampled subnetworks, the AUC values range from 0.72 to 0.82 (Fig. 4C), demonstrating that BurstLink reliably recovers gene–gene regulatory relationships even without scATAC-seq-based priors. Forth, we illustrated the necessity of quantifying regulatory interactions. Based on the priori information of the base-GRN, the gene pairs are classified into two groups with regulation (bidirectional and unidirectional regulation) and without regulation. We found that is significantly higher in the regulation group than in the nonregulation group (Fig. 4D, right), outperforming traditional measures such as R and NMI (Fig. 4D, left and middle). These results highlight that is capable of significantly amplifying the regulatory information and effectively determining gene–gene interactions.

We visualized the inferred coupled dynamics information for 4173 genes using UMAP embedding and observed that these selected genes are transcribed with varied burst kinetics and complex regulatory interaction networks (Appendix Fig. S5B). Here, we presented the inferred regulation and bursting results of one representative subnetwork as an illustrative example (Fig. 4E; Appendix Fig. S6; Appendix Table S3). Visualizations of two gene pairs (*Srf*-*Stat3* and *Cebpb*-*Hmga1*) show that *Srf* and *Stat3* positively regulate each other with different regulation strengths (Fig. 4F), while *Cebpb* negatively regulates *Hmga1* but is not reciprocally regulated (Fig. 4G), consistent with previous studies (Lynch et al, 2011; Rajotte et al, 1996). Besides, we presented more gene-pair visualization examples with diverse gene–gene regulatory interaction types (Appendix Fig. S7) and manually searched relevant literature for validation (Appendix Table S4).

To investigate transcriptional bursting kinetics across different gene roles (TFs and TGs), we categorized genes as TFs or TGs based on the inferred gene regulatory interaction network (Appendix Fig. S5B). According to graph theory, two metrics are introduced: (1) the regulating-interaction level, computed by out-degree, representing a gene’s role as a TF coordinating other genes; and (2) the regulated-interaction level, computed by in-degree in a graph, indicating a gene’s role as a TG influenced by other genes. Overall, TFs accounted for only 8.34% of the selected genes, whereas the remaining 91.66% were identified as TGs (Appendix Fig. S5D), which is consistent with the fact that transcription factors typically constitute only a small fraction of the transcriptome (Han et al, 2018). This classification identified the well-known upstream TFs (*Rest*, *Yy1*, *Klf7*, *Zeb1*, *Rest*, and *Bclaf1*; Fig. 4H) and the downstream TGs (Appendix Fig. S5E). We generated a double-layer visualization of the inferred regulatory network (Appendix Fig. S5C) based on the TF-TG classification, which enables a clear separation of upstream TFs from downstream TGs and reveals coordinated patterns of transcriptional regulation across the network.

We further compared transcriptional burst kinetics between TFs and TGs. Genome-wide analyses reveal that TGs have significantly higher BF than TFs (Fig. 4I), while BS shows no significant difference (Fig. 4J). Additionally, TGs exhibit significantly higher CV^2^ than TFs (Fig. 4K), consistent with a phenomenon known as noise propagation (Hooshangi et al, 2005; Pedraza and van Oudenaarden, 2005), where expression noise of TGs arises from TF fluctuations.

Moreover, we investigated how TF binding dynamics influence transcriptional bursting on a genome-wide scale (Appendix Fig. S5F). Specifically, we examined the affinity between TFs and their binding sites, defined by the equilibrium binding constant $\gamma ={\alpha }_{i}/{\beta }_{i},(i=1,2)$, which can be directly computed from the inferred parameters of BurstLink. Benefiting from the interpretability of our statistical mechanistic model, the affinity corresponds to the parameter $\gamma ={k}_{{{{\rm{on}}}}}/{k}_{{{{\rm{off}}}}}$ in a dynamic stochastic model (Khetan et al, 2024). The larger γ leads to a stronger tendency for TFs to bind to an empty site. As a result, we discovered that there are lower BFs and larger BSs with the increasing equilibrium binding constant γ (Fig. 4L), indicating that stronger TF binding affinity prolongs mean burst duration and enhances burst size simultaneously (Mazzocca et al, 2021; Mondal and Kolomeisky, 2024).

### Gene regulatory interactions modulate transcriptional burst kinetics on a genome-wide scale

We investigated how gene regulatory interactions influence expression variability and burst kinetics on a genome-wide scale, based on the inferred gene regulatory interactions and burst kinetics. Notably, genes exhibit widely varying inferred burst kinetics even at the same average expression level, implying that the identical expressions of different genes are regulated, possibly by different molecular mechanisms (Appendix Fig. S5B) (Suter et al, 2011). Since the gene expression levels affect the noise (Appendix Fig. S4D) (Hornung et al, 2012; Newman et al, 2006), all the selected genes are divided into two groups based on their average expression levels (high-expression group and low-expression group) to ensure unbiased comparison. We then explored this phenomenon from both macro-level and micro-level perspectives.

**Figure 5:**
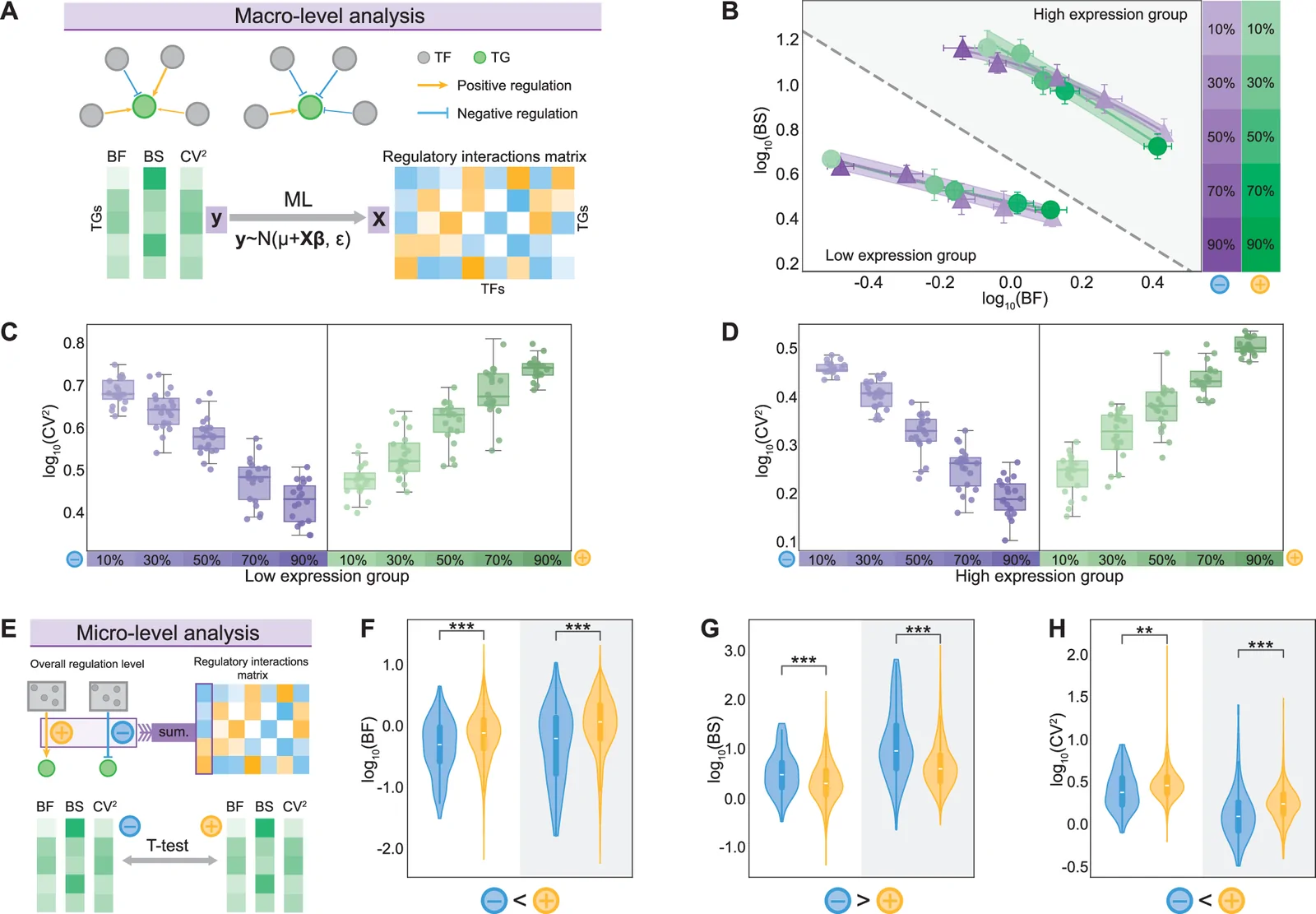
Genome-wide regulation on transcriptional burst kinetics by different gene regulatory interactions. (**A**) Schematic overview of the macro-level analysis, illustrating machine learning to study and predict the bursting kinetics in different gene regulatory loops based on the inferred results. (**B**) Scatter plot with error bars shows the predicted BF and BS under increasing proportions of negative regulations (purple) and positive regulations (green) for low-expression and high-expression groups. Error bars represent mean ± standard deviation (s.d). (**C**,** D**) Box plots showing CV^2^ across different proportions of negative and positive regulations for low-expression and high-expression gene groups. Box plots show the median (center), the 25th and 75th percentiles (box bounds), and whiskers extending to the most extreme data points within 1.5 × the interquartile range. (**E**) Schematic overview of the micro-level analysis, where individual genes are stratified by regulatory input composition, and BF, BS and CV^2^ are compared using a statistical *t*-test. (**F**–**H**) Violin plots comparing gene bursting kinetics and expression variability between positive regulations and negative regulations in low-expression and high-expression level groups. Violin plots show the data distribution. The embedded box plots show the median (center), the 25th and 75th percentiles (box bounds), and whiskers extending to the most extreme data points within 1.5 × the interquartile range. Statistical significance was assessed using a two-sided Welch’s *t*-test. The exact *P* values are: Fig. 5F, *P* = 8.97 ×  10^−5^, 1.12 × 10^−5^; Fig. 5G, *P* = 1.12 × 10^−4^, 5.16 ×  10^−7^; Fig. 5H, *P* = 0.0063 and 0.0008. The sample size is n = 4173. ML machine learning, TF transcription factor gene, TG target gene; BF burst frequency, BS burst size, CV^2^ gene-expression variability. Statistical significance is denoted as follows: *P < 0.05, **P < 0.01, and ***P < 0.001. Source data are available online for this figure.

At the macro level, we analyzed the influence of regulatory loop types on the overall gene population (Fig. 5A; see “Methods”). For each gene-expression level group, we utilized the Bayesian ridge model to predict burst kinetics and expression variability under different regulatory loop types (positive and negative regulations) and repeated the prediction procedure 20 times to ensure robustness. As a result, in both the low-expression and high-expression groups, an increasing proportion of negative regulation across the gene regulatory network is associated with decreased CV^2^ and BF but increased BS, whereas the opposite pattern is observed with an increasing proportion of positive regulation (Fig. 5B–D). At the micro level, we studied the impact of regulatory interactions on burst kinetics and expression variability based on the mean-field theory. Each gene is regulated by several specific genes within the gene regulatory interactions network. To quantify the total regulatory effect on a gene, we defined an average overall regulation level (Fig. 5E; see “Methods”). Similarly, we divided these selected genes into two groups (low and high-expression groups). Across both groups, genes with positive regulation consistently exhibit higher BFs and lower BSs compared to those genes with negative regulation (Fig. 5F,G). Moreover, the positive regulation genes show significantly higher CV^2^ than the negative regulation genes on a genome-wide scale (Fig. 5H). These results were particularly pronounced in the high-expression group, extending previous findings (Dublanche et al, 2006; Luo et al, 2023a) by incorporating gene regulatory interactions.

We further applied BurstLink to the scRNA-seq data from mouse embryonic stem (ES) cells (C57BL/6 × CAST/EiJ) and obtained the genome-wide inference results (Appendix Fig. S8A). Interestingly, BF and BS are positively correlated for overlapping genes between mouse embryonic fibroblasts and mouse ES cells, respectively (Appendix Fig. S8C,D), consistent with previous studies (Larsson et al, 2019). Additionally, most genes share the same regulatory loop types across the two gene systems (Appendix Fig. S8B), indicating that the regulatory structure is conserved at the genome-wide level.

### BurstLink identifies differential burst kinetics and regulatory interactions induced by IdU treatment through comparative analysis

**Figure 6:**
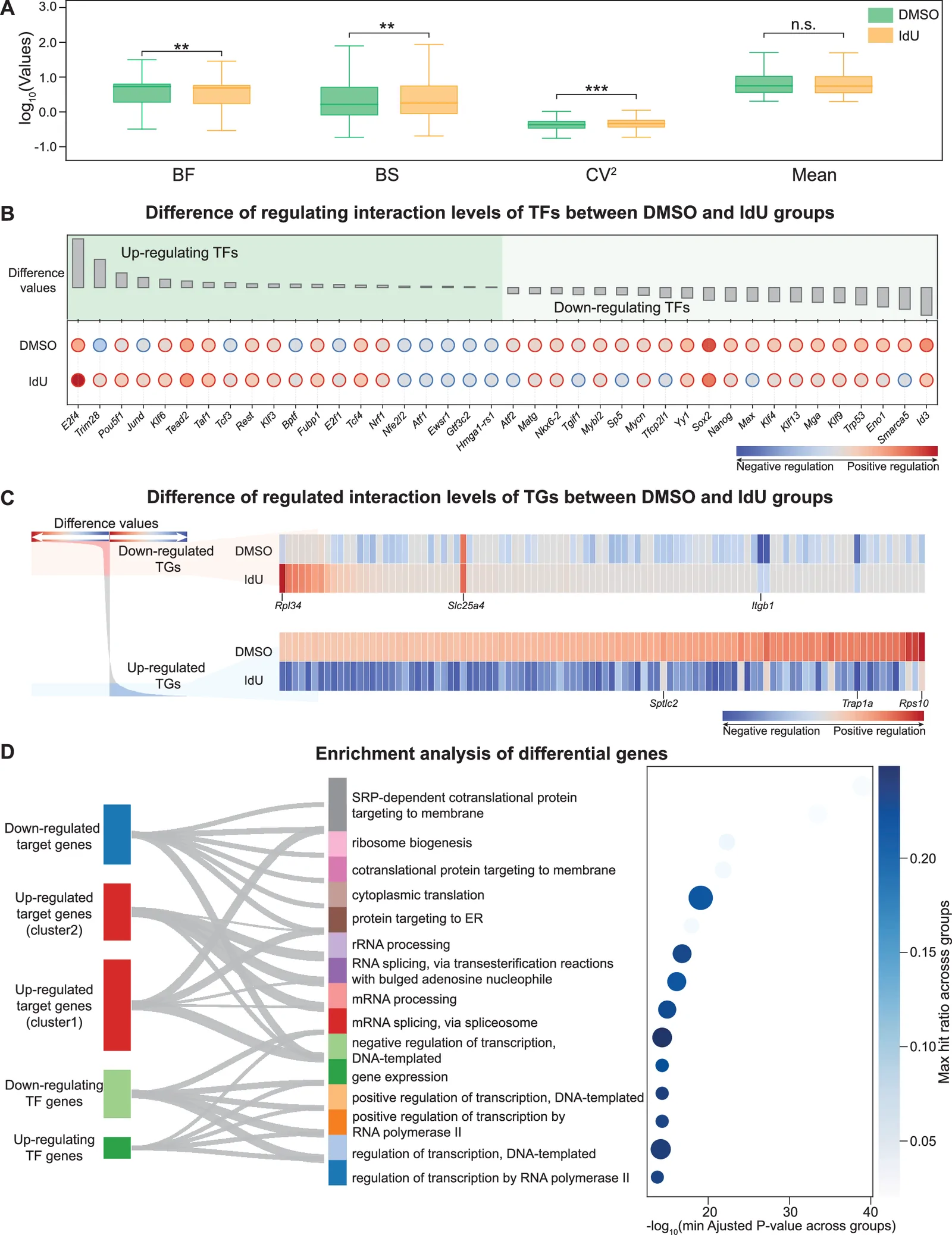
Comparison of genome-wide inference results between DMSO and IdU datasets. (**A**) Box plots for gene bursting kinetics (BF, BS), expression variability, and mean expression between DMSO and IdU groups. Statistical significance was assessed using a two-sided Welch’s *t*-test. Statistical significance is indicated as follows: *P < 0.05, **P < 0.01, and ***P < 0.001. The exact *P* values are: Fig. 6A, *P* (BF) = 0.0034, *P* (BS) = 0.0071, *P* (CV^2^) = 3.46 × 10^−16^, and *P* (MEAN) = 0.2614. The sample size is n = 642. Box plots show the median (center), the 25th and 75th percentiles (box bounds), and whiskers extending to the most extreme data points within 1.5 × the interquartile range. (**B**) Differences in regulating-interaction levels of TFs between DMSO and IdU groups. (**C**) Differences in regulated interaction levels of TGs between DMSO and IdU groups. (**D**) GO enrichment analysis of differential TFs and TGs, including upregulating TFs, downregulating TFs, upregulated TGs, and downregulated TGs. DMSO control group, IDU treatment group, DNA damage induced, BF burst frequency, BS burst size, CV^2^ gene-expression variability, TF transcription factor gene, TG target gene. Source data are available online for this figure.

BurstLink enables comparative analysis across multiple scRNA-seq datasets, distinguishing differences between control and treatment conditions. Here, we implemented BurstLink on the paired scRNA-seq data of mouse ES cells screened using base-GRN, which are treated with DMSO (control group) and 10 mM IdU (treatment group, DNA damage induced) for 24 h, respectively (Data ref: Desai et al,). The inferred gene regulatory interactions network with bursting for both DMSO and IdU groups can be visualized (Appendix Fig. S9). For differential burst kinetics Fig. 6A, IdU treatment leads to lower BF, higher BS and ${{{\mbox{CV}}}}^{2}$ without altering the average gene expression (*p* value =  0.2614), which aligns with the previous research (Desai et al, 2021; Huang et al, 2026; Khetan et al, 2024).

Next, we investigated the changes in gene regulation under IdU treatment. There are differences in regulating-interaction levels of TFs after IdU treatment, which are measured by the overall regulating level of each gene (see “Methods”). Differentially regulating TFs are identified as those whose difference value of regulating-interaction level exceeds the median of all difference values (Fig. 6B). Among these, differential upregulating genes are defined as those with increased regulating-interaction levels, while differential downregulating genes have decreased regulating interaction levels. Similarly, differential target genes are defined in the same manner based on changes in their regulated-interaction levels, with upregulated TGs showing increased regulated-interaction levels and downregulated TGs exhibiting decreased regulated-interaction levels (Fig. 6C). Gene ontology (GO) enrichment analysis on the identified differential genes, including upregulating, downregulating, upregulated, and downregulated genes sets, respectively (Appendix Fig. S10), revealing overlapping pathways linked to transcriptional regulation, particularly DNA template-dependent processes (Fig. 6D). This finding is consistent with previous experimental studies (Riccio et al, 2022; Rosina et al, 2019), indicating that IdU treatment induces DNA damage and influences gene expression through gene regulatory interactions, ultimately reshaping cell differentiation fate.

## Discussion

Complex gene–gene regulatory interactions orchestrate most cellular processes, including transcriptional bursting (Chalancon et al, 2012). In this context, there is a fundamental question arising: how do dynamic gene regulatory interactions shape burst kinetics on a genome-wide scale? To address this issue, we developed the BurstLink framework that incorporates a novel statistical mechanistic model to infer the coupled dynamics of gene regulatory interactions and transcriptional bursting across the whole GRN from single-cell multi-omics data. Whereas forward-modeling studies simulate predefined bursting kinetics and network architectures to explain coordinated expression states (Schuh et al, 2020), BurstLink solves the inverse problem, inferring these burst kinetics and directional interactions directly from single-cell data. Compared to previously developed methods (Van Dijk et al, 2018; Yuan and Bar-Joseph, 2019), BurstLink can simultaneously uncover more detailed dynamic information: (1) bidirectional regulation types (positive, negative, and nonregulation); (2) bidirectional regulation strengths, measured by asymmetric RMI; (3) regulatory interactions visualization; and (4) burst kinetics (CV^2^, BF, and BS). BurstLink has several notable advantages. First, its model is interpretable, as it corresponds to the well-established genetic toggle switch circuit (Gardner et al, 2000; Tian & Burrage, 2006), with numerical validation showing correspondence between parameter and distribution spaces across a wide parameter range. Second, the model is tractable and scalable, leveraging a common statistical distribution to enable rapid, efficient inference for genome-wide analyses. Third, the model explicitly captures the cell size variability and technical noise raised from scRNA-seq data (Tang et al, 2023). Overall, the BurstLink framework lays a solid foundation for exploring the molecular mechanisms of stochastic gene expression using single-cell data.

Applying BurstLink on single-cell multi-omics data from mouse embryonic fibroblasts (Larsson et al, 2019) provided several genome-wide insights into how transcriptional bursting is regulated by gene regulatory interactions. The upstream TFs are found to exhibit lower CV^2^ and BF than the downstream TGs, consistent with previous studies (Trzaskoma et al, 2024). Moreover, BurstLink revealed that transcriptional bursting is influenced by TF binding dynamics. Stronger binding affinity results in lower BF and higher BS (Mondal and Kolomeisky, 2024). Most importantly, positive regulations are associated with higher CV^2^ and BF, but lower BS compared to negative regulations, with these patterns observed both at the population and individual gene levels. These findings extend prior single-gene experiments (Dublanche et al, 2006; Müller-McNicoll et al, 2019) and our previous genome-wide study on autoregulation (Luo et al, 2023a).

Our work paves the way for several avenues of further research. The BurstLink framework is based on the Poisson representation, a well-established approach for modeling steady-state solutions in both single-gene expression dynamics (Vu et al, 2016) and complex stochastic gene expression regulatory networks (Wang et al, 2023b). While our results demonstrate numerical correspondence between our model and the dynamic mechanistic model, we acknowledged that the current framework remains an approximation. Deriving a closed-form Poisson representation from a generalized toggle-switch or GRN model that encompasses positive, negative, and non-regulatory interactions remains a major theoretical challenge. Future work could focus on developing dynamic models for gene pairs or entire regulatory systems that incorporate tractable statistical distributions to directly represent steady-state solutions. Additionally, BurstLink currently uses the Sarmanov–Lee distribution to link marginal distributions of each gene pair within the copula framework (Jaworski et al, 2010; Sarabia and Gomez-Deniz, 2011), forming a GRN with bursting kinetics. Although the linear noise approximation provides analytical tractability (Thomas et al, 2014), it assumes large copy numbers and small fluctuations, making it unsuitable for the discrete and over-dispersed mRNA counts typical of scRNA-seq data. Similarly, the analytical toggle-switch solution applies only to symmetric mutual-repression architectures under strong repression limits, and thus cannot generalize to diverse regulatory types across genome-wide networks (Biancalani and Assaf, 2015). Our approach could be refined by adopting the conditional copula method (Krupskii and Joe, 2013), which would offer a more flexible and precise representation of regulatory relationships. Another limitation arises from the inference relying on statistical distributions under certain assumptions. To overcome the assumption of independence between gene pairs, the inference method could be expanded to a multivariate global model using pseudo-maximum likelihood estimation (Gong and Samaniego, 1981). Correspondingly, the utilization of bivariate RMI to quantify regulatory strengths could be extended to multivariate approaches, leveraging multivariate information entropy to capture more complex multi-gene interactions (Chan et al, 2017; Timme et al, 2014). Relatedly, the directional dependencies BurstLink extracts from the asymmetry of steady-state conditional distributions should be understood as putative regulatory directions rather than interventional causality, since snapshot data alone cannot establish causation without perturbation or temporal measurements. When combined with biologically informed GRN priors (Stock et al, 2025), such as TF-target relationships, chromatin accessibility, motif information, or curated regulatory databases, these directions are further constrained and may be interpreted as biologically plausible causal relationships, though definitive validation ultimately requires perturbation experiments. Moreover, model unidentifiability is a significant challenge in statistical inference. To address this issue, various strategies can be utilized, incorporating regularization, prior knowledge from multi-omics data, and Bayesian methods, thereby enhancing the reliability and accuracy of the model (Cao and Grima, 2018, 2020; Jiang et al, 2021).

Recent advancements in deep learning provide opportunities to address complex stochastic systems with both computational efficiency and accuracy (Fan et al, 2024; Jiang et al, 2021; Li et al, 2023; Michoski et al, 2020; Stuart et al, 2019; Wang et al, 2019a). Nessie approximated solutions to the chemical master equations using neural networks (Sukys et al, 2022), and biVI combined the variational autoencoder framework of scVI with biophysical models to describe the RNA transcription and splicing kinetics (Carilli et al, 2024). In addition, DeepTX solved non-Markovian dynamic systems by utilizing neural networks and inferred genome-wide transcriptional bursting kinetics under DNA damage conditions (Huang et al, 2026). These studies highlight deep learning as a powerful tool for further modeling and inferring more detailed coupled dynamics of gene regulatory interactions and transcriptional bursting.

A key limitation of BurstLink, shared by scRNA-seq-based regulatory inference more generally, is that it accesses only transcript abundance, whereas the regulatory activity it aims to capture is ultimately exerted by transcription factors at the protein level. Because protein half-lives are typically longer than those of mRNA (Schwanhäusser et al, 2011), TF protein levels are buffered relative to, and lag behind, transcript fluctuations, so regulator mRNA provides only an approximate readout of regulatory activity. Nevertheless, simulations incorporating explicit protein dynamics with a realistic mRNA–protein timescale separation show that BurstLink can still recover the qualitative regulatory type and direction, though the inferred coupling strength is attenuated (Appendix Text S1.4; Appendix Fig. S11). Capturing regulatory layers beyond transcript abundance would therefore help close this gap. While BurstLink already utilizes scATAC-seq data to filter scRNA-seq data, advancements in single-cell multi-omics technologies offer additional opportunities to investigate transcriptional bursting kinetics and gene regulatory mechanisms more comprehensively (Stock et al, 2025; Subramanian et al, 2020). For example, scATAC-seq data can be further leveraged to provide deeper insights into chromatin accessibility at a higher resolution (Buenrostro et al, 2015; Schep et al, 2015). Besides, other single-cell data types can be integrated, such as scGRO-seq data, which estimates burst frequency and size by directly quantifying transcribing RNA polymerase dynamics (Mahat et al, 2024), and scHi-C, which captures 3D chromatin conformation (Belton et al, 2012; Wang et al, 2023c). Combining these multi-omics data into a unified framework would allow for regulatory mechanisms and transcriptional dynamics that may not be fully captured by scRNA-seq data alone, ultimately providing a thorough understanding of gene expression regulation (Vandereyken et al, 2023).

Finally, in our current work, we simplified both the transcription process and bursting kinetics (Chalancon et al, 2012; Hebenstreit and Karmakar, 2024), which can be further refined and expanded. Additional biological complexities could be integrated into the model, such as preloaded RNAPII, RNA splicing kinetics (Carilli et al, 2024; Eling et al, 2019), and co-bursting dynamics (Ramsköld et al, 2024). Our inference is currently performed on mature mRNA counts. Jointly modeling unspliced and spliced counts (Eling et al, 2019), which are now routinely recovered from scRNA-seq data (La Manno et al, 2018), could improve the identifiability of burst kinetic parameters, particularly the separation of transcription from degradation rates, as exploited by recent approaches (Carilli et al, 2024; Gorin et al, 2022). Moreover, this research strategy could be extended to other areas, such as investigating the effects of 3D chromatin architecture and enhancer-mediated regulation (Bartman et al, 2016; Fukaya et al, 2016; Mermet et al, 2018; Trzaskoma et al, 2024). In particular, the regulation inferred by BurstLink represents a generalized regulatory effect on target-gene activation, so that enhancer-mediated regulation is implicitly subsumed within this effective term rather than explicitly resolved. Distinguishing promoter- from enhancer-mediated regulatory effects is an important direction for future refinement. The cell cycle offers another promising direction (Sukys and Grima, 2025; Volteras et al, 2024). While cell-size-associated scaling already captures one route of cell-cycle influence in our framework, phase-specific transcriptional programs and cell-cycle-dependent regulatory activity remain to be modeled explicitly (Padovan-Merhar et al, 2015). Our framework already accounts for measurement noise by modeling transcript capture as an independent binomial sampling process, under which the bivariate Poisson–Beta distribution is preserved (Appendix Text S1.5). Real scRNA-seq measurements, however, involve more complex processes, such as the fixed sequencing depth that induces a multinomial sampling structure and additional technical biases, under which this invariance no longer holds exactly. Developing inference frameworks that more fully account for such measurement processes is an important direction for future work. In conclusion, BurstLink serves as a bridge between dynamic gene regulatory interactions and transcriptional bursting, providing deeper insights into biological inquiries.

## Methods

**Table:** Reagents and tools table

Reagent/resource	Reference or source	Identifier or catalog number
**Experimental models**		
**Recombinant DNA**		
**Antibodies**		
**Oligonucleotides and other sequence-based reagents**		
**Chemicals, enzymes and other reagents**		
**Software**		
R-software v4.3.2	https://www.r-project.org/	
Python v3.10	https://www.python.org/	
**Other**		

### BurstLink: a statistical mechanistic model for inferring gene–gene regulatory interactions and transcriptional bursting kinetics

#### Model overview

We proposed a statistical mechanistic model, BurstLink, that allows an easily representable statistical distribution to simultaneously characterize the dynamic properties of gene–gene regulatory interactions and burst kinetics at genome-wide scale, which are often covered by a complex stochastic dynamical gene-expression model. Specifically, we view the GRN as a graph *G=*{Ω, ℇ}, where Ω={*i*,*i*=1,2,…,*n*} denotes the set of genes (nodes) and $\varepsilon=\left\{e,e=\left(i,j\right),\forall i,j\in \Omega \right\}$ denotes the set of gene pairs (edges). For each gene $i\in \Omega$, its observed expression values across cells,${X}_{i}=\left\{{x}_{i,k},\,k=1,\,2,\,\ldots ,{C}\right\}$, are assumed to follow a Poisson–Beta mixture distribution (Sarabia and Gomez-Deniz, 2011):

1 $${x}_{{ik}}| {\eta }_{i}\sim 	 \, {{\mbox{Poisson}}}\left({\phi }_{i}{\tau }_{k}{s}_{k}{\eta }_{i}\right),\\ {\eta }_{i}\sim 	 \, {{\mbox{Beta}}}\left({\alpha }_{i},{\beta }_{i}\right),$$

where each random variable ${X}_{i}$ $(i\in \Omega )$ follows a univariate Poisson–Beta distribution, respectively, describing single-gene transcriptional bursting kinetics from the classic ON-OFF model (Kim and Marioni, 2013). Note that the gene expression kinetic parameters can be directly interpreted via our statistical mechanistic model: the shape parameters ${\alpha }_{i}$ $(i\in \Omega )$ and scale parameters ${\beta }_{i}$ $(i\in \Omega )$ of the Beta distribution correspond to switching rates between inactive and active states, and the parameters ${\phi }_{i}$ $(i\in \Omega )$ of the Poisson distribution correspond to the mRNA transcriptional rates. Moreover, cell-specific effects are incorporated in the model, with ${\tau }_{k}$ denoting the capture efficiency and ${s}_{k}$ denoting the cell size (Tang et al, 2023). The random variable ${\eta }_{i}$ $(i\in \Omega )$ links the Poisson distribution and Beta distribution, the mean of which represents the percentage of time gene’s state that genes are in an active state.

Importantly, $L({\eta }_{i},{\eta }_{j})$ serves as a linking function to describe potential dependencies between any two variables ${X}_{i}$ and ${X}_{j}$, which represents a multivariate distribution with Beta marginals ${{\mbox{Beta}}}({\alpha }_{i},{\beta }_{i})$,

2 $$\forall \left({\eta }_{i},{\eta }_{j}\right)\sim L\left({\eta }_{i},{\eta }_{j}\right),i,j\in \Omega .$$

Accordingly, any two binary variables follow a bivariate Poisson–Beta mixture distribution. Here, we utilize the Sarmanov–Lee distribution as a specific distribution form

3 $$L\left({\eta }_{i},{\eta }_{j}\right)={f}_{i}\left({\eta }_{i}\right){f}_{j}\left({\eta }_{j}\right)\left[1+w{h}_{i}\left({\eta }_{i}\right){h}_{j}\left({\eta }_{j}\right)\right],$$

where ${f}_{i}({\eta }_{i})$ is the probability density function of the marginal Beta distribution ${{\mbox{Beta}}}({\alpha }_{i},{\beta }_{i})$. ${h}_{i}$ is a bounded non-constant function such that

4 $$\int {f}_{i}\left(z\right){h}_{i}\left(z\right){dz}=0,i\in \Omega .$$

We set ${h}_{i}({\eta }_{i})={\eta }_{i}-{\alpha }_{i}/({\alpha }_{i}+{\beta }_{i})$ for $i\in \Omega$. The parameter ${w}_{e}$ ($e=(i,j)\,{\in}\,{E}$) controls the correlation between two variables (see details in Supplementary Text).

5 $$-\frac{\left({\alpha }_{i}+{\beta }_{i}\right)\left({\alpha }_{j}+{\beta }_{j}\right)}{\max \left\{{\alpha }_{i}{\alpha }_{j},{\beta }_{i}{\beta }_{j}\right\}}\le {w}_{e}\le \frac{\left({\alpha }_{i}+{\beta }_{i}\right)\left({\alpha }_{j}+{\beta }_{j}\right)}{\max \left\{{\alpha }_{i}{\beta }_{j},{\alpha }_{j}{\beta }_{i}\right\}}.$$

Specifically, the symbol of w represents the regulation loop type (positive regulation loop for w>0, negative regulation loop for w<0, and nonregulation for w=0). Besides, RMI is a function of w to quantify the directional regulation (Appendix Text).

Here, we highlight that BurstLink can simultaneously capture expression variability and transcriptional burst kinetics for each gene, as well as regulatory interaction between any two genes:

1. Expression variability: Gene expression variability is introduced to quantify stochastic fluctuation in gene expression, which is mathematically defined as the square of the coefficient of variation, i.e., the ratio of the variance over the square of the mean. For a gene-expression counts variable ${X}_{g}$ of gene g with the parameters ${\theta }_{g}$ from the generative mechanism, the theoretical gene-expression variability is
  6 $${{{\rm{CV}}}}_{g}^{2}=\frac{{{{\rm{Var}}}}\left[{X}_{g};{\theta }_{g}\right]}{{\mathbb{E}}{[{X}_{g};{\theta }_{g}]}^{2}}.$$
2. Burst frequency: the number of bursts occurring within a given period, which is expressed as
  7 $$\begin{array}{cc}{{{{\rm{BF}}}}}_{i}=\frac{1}{1/{\alpha }_{i}+1/{\beta }_{i}}, & i=1,2,\ldots ,n.\end{array}$$
3. Burst size: the mean number of mRNA products generated per burst, which is expressed as
  8 $$\begin{array}{cc}{{{{\rm{BS}}}}}_{i}=\frac{{\phi }_{i}}{{\beta }_{i}}, & i=1,2,\ldots ,n.\end{array}$$
4. Regulation interactions: Two complementary statistics are inferred to quantify gene–gene regulatory interactions, corresponding to regulatory type and regulatory strength, respectively. Regulatory type is determined from systematic trends in the rescaled conditional probabilities, which highlight directional shifts in the conditional response of one gene to another. Regulatory strength is quantified using RMI, computed on a reconstructed probability space to measure the magnitude of statistical dependence between gene pairs,

9 $$\begin{array}{c}{{{{\rm{RMI}}}}}_{{X}_{i}|{X}_{j}}= {\sum}_{n}{\sum} _{m}Pr \left({X}_{i}=n,|,{X}_{j}=m\right)\log \left[\frac{Pr \left({X}_{i}=n,{X}_{j}=m\right)}{Pr \left({X}_{i}=n\right)Pr \left({X}_{j}=m\right)}\right],\\ {{{{\rm{RMI}}}}}_{{X}_{j}|{X}_{i}}= {\sum} _{m}{\sum} _{n}Pr \left({X}_{j}=m,|,{X}_{i}=n\right)\log \left[\frac{Pr \left({X}_{j}=m,{X}_{i}=n\right)}{Pr \left({X}_{j}=m\right)Pr \left({X}_{i}=n\right)}\right],\end{array}$$

where Pr(⋅) denotes probabilities defined on the reconstructed joint distribution, and $Pr (\cdot {{{\rm{| }}}}\cdot )$ denotes the corresponding conditional probabilities (see details in the Inference section and Appendix Fig. S14B). We have added theoretical analyses to explain how BurstLink infers regulatory directionality from snapshot data by using asymmetric information encoded in steady-state conditional distributions (Appendix Text S1.3). We also included a simple gene-network example, together with a schematic illustration (Appendix Fig. S12).

#### Model solution

Based on the formula of total probability, the joint probability mass function of the any two gene products ${{{\boldsymbol{X}}}}=({X}_{i},{X}_{j})=\left\{({X}_{{ik}},{X}_{{jk}}),k=1,2,...{{\mbox{C}}}\right\}$ can be expressed as follows (see details in Appendix Text S1.1):

10 $$Pr 	 \left( {X}_{{ik}}={x}_{{ik}},{X}_{{jk}}={x}_{{jk}}\right) \\ = 	 {\int }_{{[0,1]}^{2}}{\prod} _{l=\left(i,j\right)}\frac{{e}^{-{\phi }_{l}{\tau }_{k}{s}_{k}{\eta }_{l}}{({\phi }_{l}{\tau }_{k}{s}_{k}{\eta }_{l})}^{{x}_{{lk}}}}{{x}_{{lk}}!}{f}_{i}\left({\eta }_{i}\right){f}_{j}\left({\eta }_{j}\right)\left[1+{w}_{\left(i,j\right)}{h}_{i}\left({\eta }_{i}\right){h}_{j}\left({\eta }_{j}\right)\right]d{\eta }_{i}d{\eta }_{j}\\ = 	 {Q}_{{\alpha }_{i},{\beta }_{i},{\phi }_{i}}\left({x}_{{ik}}\right){Q}_{{\alpha }_{j},{\beta }_{j},{\phi }_{j}}\left({x}_{{jk}}\right)+{w}_{\left(i,j\right)}\times {\mu }_{i}{\mu }_{j}\times \left[{Q}_{{\alpha }_{i}+1,{\beta }_{i},{\phi }_{i}}\left({x}_{{ik}}\right)-{Q}_{{\alpha }_{i},{\beta }_{i},{\phi }_{i}}\left({x}_{{ik}}\right)\right] \\ 	 \times \left[{Q}_{{\alpha }_{j}+1,{\beta }_{j},{\phi }_{j}}\left({x}_{{jk}}\right)-{Q}_{{\alpha }_{j},{\beta }_{j},{\phi }_{j}}\left({x}_{{jk}}\right)\right],$$

where ${\mu }_{i}={\alpha }_{i}/({\alpha }_{i}+{\beta }_{i})$ $(i\in \Omega )$ represents the mean of the marginal Beta distribution, and ${Q}_{{\alpha }_{i},{\beta }_{i},{\phi }_{i}}({x}_{{ik}})$ $(i\in \Omega )$ denotes the probability mass function of the univariate Poisson–Beta distribution, which can be expressed as (Larsson et al, 2019)

11 $${Q}_{{\alpha }_{i},{\beta }_{i},{\phi }_{i}}\left({x}_{{ik}}\right)\,\triangleq \,Pr \left({X}_{{ik}}={x}_{{ik}}\right)=\frac{1}{B\left({\alpha }_{i},{\beta }_{i}\right)}{\int }_{\!\!\!\!0}^{1}\frac{{e}^{-{\phi }_{i}{\tau }_{k}{s}_{k}{\eta }_{i}}{({\phi }_{i}{\tau }_{k}{s}_{k}{\eta }_{i})}^{{x}_{{ik}}}}{{x}_{{ik}}!}{\eta }_{i}^{{\alpha }_{i}-1}{(1-{\eta }_{i})}^{{\beta }_{i}-1}{{{\rm{d}}}}{\eta }_{i},i\in \Omega ,$$

where $B({\alpha }_{i},{\beta }_{i})={\int }_{\!\!0}^{1}{t}^{{\alpha }_{i}-1}{(1-t)}^{{\beta }_{i}-1}$ ${{{\rm{d}}}}t$ is the Beta function. Hence, the bivariate joint probability can be transformed into several univariate probability functions, facilitating subsequent calculations. The conditional probabilities of ${X}_{{ik}}$ and ${X}_{{jk}}$ are given by

$$	 Pr \left({X}_{{ik}}={x}_{{ik}}|{X}_{{jk}}={x}_{{jk}}\right)={Q}_{{\alpha }_{i},{\beta }_{i},{\phi }_{i}}\left({x}_{{ik}}\right)+{w}_{\left(i,j\right)}{\mu }_{i}{\mu }_{j} \\ 	 \times \left[{Q}_{{\alpha }_{i}+1,{\beta }_{i},{\phi }_{i}}\left({x}_{{ik}}\right)-{Q}_{{\alpha }_{i},{\beta }_{i},{\phi }_{i}}\left({x}_{{ik}}\right)\right]\times \left[{Q}_{{\alpha }_{j}+1,{\beta }_{j},{\phi }_{j}}\left({x}_{{jk}}\right)/{Q}_{{\alpha }_{j},{\beta }_{j},{\phi }_{j}}\left({x}_{{jk}}\right)-1\right],$$

12 $$	 Pr \left({X}_{{jk}}={x}_{{jk}}|{X}_{{ik}}={x}_{{ik}}\right)={Q}_{{\alpha }_{j},{\beta }_{j},{\phi }_{j}}\left({x}_{{jk}}\right)+{w}_{\left(i,j\right)}{\mu }_{i}{\mu }_{j} \\ 	 \times \left[{Q}_{{\alpha }_{j}+1,{\beta }_{j},{\phi }_{j}}\left({x}_{{jk}}\right)-{Q}_{{\alpha }_{j},{\beta }_{j},{\phi }_{j}}\left({x}_{{jk}}\right)\right]\times \left[{Q}_{{\alpha }_{i}+1,{\beta }_{i},{\phi }_{i}}\left({x}_{{ik}}\right)/{Q}_{{\alpha }_{i},{\beta }_{i},{\phi }_{i}}\left({x}_{{ik}}\right)-1\right].$$

Here, we emphasize the significance of the above conditional probabilities, as both the calculation of RMI as regulation strengths and the Gibbs sampling (see details in Appendix Text S1.1) for generating the synthetic scRNA-seq data are based on Eq. 12.

### Equivalence between the statistical mechanistic model and the dynamic mechanistic model

#### A dynamic mechanistic model for describing regulatory interactions and transcriptional bursting

To demonstrate the ability of our statistical mechanistic model to infer these dynamic properties of regulatory interactions and transcriptional bursting, we first proposed a discrete, stochastic model of gene–gene interaction with transcriptional burst, which is generally known as the genetic toggle switch circuit (Tian and Burrage, 2006). There are two fundamental biological viewpoints to be satisfied for this dynamic mechanistic model: (1) the transcription of genes to produce mRNA is intermittent; (2) proteins are synthesized through the processes of gene translation, and further act as transcription factors to potentially regulate the expression of target genes. We assumed in our model that (1) genes toggle between inactive (OFF) and active (ON) states with switching rates; (2) the binding of transcription factors to the other genes will alter the rate of mRNA synthesis (promoting or inhibiting), thus resulting in a gene–gene regulatory interactions loop. Therefore, the biochemical reactions describing the mechanism of gene regulatory interactions and transcriptional bursting can be written as follows

13 $$	 {{\mathrm{OFF}}}_{X}{\to }^{{k}_{{on},{reg}}^{X}(m)}{{\mathrm{ON}}}_{X},\,{{\mathrm{ON}}}_{X}{\to }^{{k}_{{off}}^{X}}{{\mathrm{OFF}}}_{X},\,{{\mathrm{ON}}}_{{{\rm{X}}}}{\to }^{{k}_{{syn}}^{X}}{{\mathrm{ON}}}_{X}+X,\\ 	 {{\mathrm{OFF}}}_{Y}{\to }^{{k}_{{on},{reg}}^{Y}(n)}{{\mathrm{ON}}}_{Y},\,{{\mathrm{ON}}}_{Y}{\to }^{{k}_{{off}}^{Y}}{{\mathrm{OFF}}}_{Y},\,{{\mathrm{ON}}}_{{{\rm{Y}}}}{\to }^{{k}_{{syn}}^{Y}}{{\mathrm{ON}}}_{Y}+Y,\\ 	 X{\to }^{{k}_{{deg}}^{X}}\varnothing ,\,Y{\to }^{{k}_{{deg}}^{Y}} \varnothing ,$$

where X and Y denote mRNA transcripts of two genes, respectively. ${k}_{{{{\rm{on}}}},{{{\rm{reg}}}}}^{{{{\rm{X}}}}}(\cdot )$ and ${k}_{{{{\rm{on}}}},{{{\rm{reg}}}}}^{{{{\rm{Y}}}}}(\cdot )$ are the switching rates between ${{{\rm{OF}}}}{{{{\rm{F}}}}}_{{{{\rm{c}}}}}$ and ${{{\rm{O}}}}{{{{\rm{N}}}}}_{{{{\rm{c}}}}}$ states, respectively. The parameters ${k}_{{{{\rm{syn}}}}}^{{{{\rm{c}}}}}$ and ${k}_{{{\mbox{deg}}}}^{{{{\rm{c}}}}}$ are the mRNA synthesis and degradation rates. Here, ${{{\rm{c}}}}={{{\rm{X}}}},{{{\rm{Y}}}}$.

Note that the activation rate ${k}_{{{{\rm{on}}}},{{{\rm{reg}}}}}^{{{{\rm{c}}}}}(\cdot )$ depends on the account of proteins (TFs) translated from mRNA transcripts. Let n and m denote the number of the mRNA transcripts X and Y, respectively. We assume that the effective regulatory input is a monotonic function of regulator mRNA abundance and use the simplest first-order approximation, ${r}_{{{{\rm{X}}}}}\cdot X$ and ${r}_{{{{\rm{Y}}}}}\cdot Y$, where ${r}_{{{{\rm{X}}}}}$ and ${r}_{{{{\rm{Y}}}}}$ are scaling coefficients (Liu et al, 2016). These terms should be interpreted as coarse-grained, phenomenological representations of regulatory activity that enter the Hill functions. We confirmed that the inferred regulatory interactions are robust to the precise values of these scaling coefficients through a sensitivity analysis in which r are varied across a wide range (Appendix Text S1.4; Appendix Fig. S13). Regulatory effects on gene activation are then modeled using the following Hill functions

14 $${k}_{{{{\rm{on}}}},{{{\rm{reg}}}}}^{{{{\rm{X}}}}}\left(m\right)={k}_{{{{\rm{on}}}}}^{{{{\rm{X}}}}}\left[\frac{{({K}_{{{{\rm{Y}}}}})}^{{h}_{{{{\rm{Y}}}}}}}{{({K}_{{{{\rm{Y}}}}})}^{{h}_{{{{\rm{Y}}}}}}+{({r}_{{{{\rm{Y}}}}}m)}^{{h}_{{{{\rm{Y}}}}}}}+{\epsilon }_{{{{\rm{X}}}}}\right], \\ {k}_{{{{\rm{on}}}},{{{\rm{reg}}}}}^{{{{\rm{Y}}}}}\left(n\right)={k}_{{{{\rm{on}}}}}^{{{{\rm{Y}}}}}\left[\frac{{({K}_{{{{\rm{X}}}}})}^{{h}_{{{{\rm{X}}}}}}}{{({K}_{{{{\rm{X}}}}})}^{{h}_{{{{\rm{X}}}}}}+{({r}_{{{{\rm{X}}}}}n)}^{{h}_{{{{\rm{X}}}}}}}+{\epsilon }_{{{{\rm{Y}}}}}\right],$$

where ${k}_{{{{\rm{bas}}}}}^{{{{\rm{c}}}}}={k}_{{{{\rm{on}}}}}^{{{{\rm{c}}}}}\cdot {\epsilon }_{{{{\rm{c}}}}}$ (${{{\rm{c}}}}={{{\rm{X}}}},{{{\rm{Y}}}}$) are the basic activation switching rates, ${K}_{{{{\rm{c}}}}}$ (${{{\rm{c}}}}={{{\rm{X}}}},{{{\rm{Y}}}}$) denote the dissociation constants between regulatory region (such as promoter and enhancer) and TFs, and ${h}_{{{{\rm{c}}}}}$ (${{{\rm{c}}}}={{{\rm{X}}}},{{{\rm{Y}}}}$) are the Hill coefficients corresponding to negative regulation when ${h}_{{{{\rm{c}}}}} > 0$, positive regulation when ${h}_{{{{\rm{c}}}}} < 0$, and nonregulation when ${h}_{{{{\rm{c}}}}}=0$.

Let P(n,m,t) be the probability vector that there are n and m mRNA transcripts at time t for two genes, respectively. The evolution of the above mechanism model is governed by the following set of chemical master equations (CMEs)

15 $$\frac{\partial {{{\boldsymbol{P}}}}\left(n,m,t\right)}{\partial t}={{{\boldsymbol{MP}}}}\left(n,m,t\right),$$

where ${{{\boldsymbol{M}}}}$ represents the state-transition matrix (Appendix Text).

To obtain the synthetic data and mRNA stationary distribution from the genetic switch circuit model, we used the stochastic simulation algorithm (Gillespie, 1976) that allows simulating gene expression counting tracks from dynamics systems described by CMEs. The synthetic scRNA-seq data generated via SSA is utilized to numerically prove the equivalence of parameters and landscapes between the statistical mechanistic model and the dynamic mechanistic model in the next section.

#### Mapping the parameters and landscapes from the statistical mechanistic model to the dynamic mechanistic model

To prove that our statistical model can describe the steady-state solution of the toggle switch mechanistic model, we numerically demonstrated that the parameters and solution landscapes of these two models can correspond to each other. We generated the synthetic scRNA-seq data given the parameters ${\theta }_{{\mbox{mech}}}=\left({k}_{{\mbox{on}},}^{X},{k}_{{\mbox{off}}}^{X},{k}_{{\mbox{on}}}^{Y},{k}_{{\mbox{off}}}^{Y},{k}_{{\mbox{syn}}}^{X},{k}_{{\mbox{syn}}}^{Y},{k}_{{\mbox{deg}}}^{X},{k}_{{\mbox{deg}}}^{Y},{h}_{X},{h}_{Y},{r}_{X},{r}_{Y},{K}_{X},{K}_{Y},{\epsilon }_{X},{\epsilon }_{Y}\right)$ of the genetic toggle switch mechanistic model via SSA. Then we performed the inference procedure on the statistical mechanistic model to obtain the inferred parameters ${\hat{\theta }}_{{{\rm{stat}}}}=\left({\hat{\alpha }}_{1},{\hat{\alpha }}_{2},{\hat{\beta }}_{1},{\hat{\beta }}_{2},{\hat{\phi }}_{1},{\hat{\phi }}_{2},\hat{w}\right)$.

For the mapping of the landscapes between the statistical mechanistic model and the dynamic mechanistic model, we employed the generalized Kolmogorov–Smirnov (KS) test to evaluate whether the Gibbs samples distribution with the inferred parameters ${\hat{\theta }}_{{{{\rm{stat}}}}}$ is consistent with the synthetic data distribution generated under the parameters ${\theta }_{{{{\rm{mech}}}}}$. The generalized KS distance is computed as

16 $${D}_{n}=\max \left\{{\sup }_{x{{\in }}{{{\mathscr{X}}}}}\left[{F}_{n}\left(x\right)-F\left(x\right)\right],\,{\sup }_{x{{{\mathscr{\in }}}}{{{\mathscr{X}}}}}\left[F\left(x\right)-{F}_{n}\left(x\right)\right]\right\},$$

where ${F}_{n}$ is the empirical cumulative distribution function (eCDF), and F is the distribution generated via the inferred parameters. The generalized KS distance between the eCDF of the simulated data via SSA and the expected distribution by a Gibbs sampler with the inferred parameters can be calculated according to Eq. 16. The inferred parameters are considered to have a good fit if the generalized KS distance is smaller than at least 5% of the simulated cases (Fig. 2B).

For the mapping of the parameters between the statistical mechanistic model and the dynamic mechanistic model, we extensively generated simulated scRNA-seq data under different OFF–ON switch rates $\left({\left\langle {k}_{{{{\rm{on}}}},{{{\rm{reg}}}}}^{X}(m)\right\rangle }_{m},{k}_{{{{\rm{off}}}}}^{X}\right)$ and synthetic rate ${k}_{{{{\rm{syn}}}}}^{X}$, respectively, and then obtained the corresponding inferred parameters $({\hat{\alpha }}_{1},{\hat{\beta }}_{1},{\hat{\phi }}_{1})$. We performed correlation regression analysis on these corresponding parameter pairs $\left({\left\langle {k}_{{{{\rm{on}}}},{{{\rm{reg}}}}}^{X}(m)\right\rangle }_{m},{\hat{\alpha }}_{1};{k}_{{{{\rm{off}}}}}^{X},{\hat{\beta }}_{1};{k}_{{{{\rm{syn}}}}}^{X},{\hat{\phi }}_{1}\right)$ (Fig. 2C). Note that we performed correlation analysis on a single variable while keeping other parameters as constants. For detailed parameter settings, see Appendix Table S2.

### Inference for the BurstLink framework

#### Network-wide inference

Building on our statistical mechanistic model, the network-wide inference for gene regulatory interactions and gene-specific bursting kinetics can be formulated as the following optimization problem

17 $${\min }_{\left\{{\theta }_{i}\right\},\left\{{w}_{e}\right\}}{\sum}_{e{{{\mathcal{\in }}}}{{{\mathcal{E}}}}}-{{{\mathrm{ln}}}}\left[{Pr }_{e}\left({\theta }_{i},{\theta }_{j},{w}_{e};{{{\boldsymbol{x}}}}\right)\right],\forall i \in \Omega ,e=\left(i,j\right){\,{\in }}\,{{{\mathcal{E}}}},$$

where ${Pr }_{e}(\cdot )$ represents the likelihood associated with the bivariate Poisson–Beta distribution (Eq. 10). The entire prescreened gene regulatory network is represented as a graph $G=\left\{\Omega ,{{{\mathcal{E}}}}\right\}$, in which every gene pair corresponding to an edge is modeled based on the bivariate Poisson–Beta distribution. For any edge $e=(i,j){\in}{E}$ linking nodes i and j, the inferred parameters ${\theta }_{i}=({\alpha }_{i},{\beta }_{i},{\phi }_{i})\in {{\mathbb{R}}}^{3}$ ($i\in \Omega$) correspond to the expression-related parameters of gene i, and ${w}_{e}$ encodes the regulatory interactions between two genes, assigning the parameter to each edge.

Note that each node $i\in \Omega$ is associated with a unique three-dimensional parameter vector, which is shared across all edges connected to i, and each potential edge $e{{{\mathscr{\in }}}}{{{\mathcal{E}}}}$ carries an additional scalar parameter ${w}_{e}$. Consequently, the parameters of gene i enter the loss for every gene pair e=(i,j), so updating them simultaneously changes many edge-specific likelihoods. This strong coupling, together with the large number of genes and edges, makes inferring all parameters from high-dimensional scRNA-seq data in large gene regulatory networks a substantially challenging optimization problem. Inspired by ADMM (Neal et al, 2011), a decoupled and fully parallel treatment to address this challenge is introducing multiple edge-specific local variables ${\lambda }_{i,e}=({\alpha }_{i,e},{\beta }_{i,e},{\phi }_{i,e})\in {{\mathbb{R}}}^{3}$ $(i \in \Omega )$ together with node-specific consensus variables ${\theta }_{i}$ $(i \in \Omega )$ to decouple the problem while preserving global consistency. Accordingly, the original optimization can be equivalently reformulated as follows:

18 $${\min }_{\left\{{\theta }_{i}\right\},\left\{{\lambda }_{i,e}\right\},\left\{{w}_{e}\right\}}{\sum}_{e{{{\mathscr{\in }}}}{{{\mathscr{E}}}}}-{{{\mathrm{ln}}}}\left[{\Pr }_{e}\left({\lambda }_{i,e},{\lambda }_{j,e},{w}_{e};{{{\boldsymbol{x}}}}\right)\right],{{\mbox{s.t.}}}{\lambda }_{i,e}={\theta }_{i},\forall i\in \Omega ,e{{{\mathscr{\in }}}}{{{\mathscr{E}}}},$$

where the constraints ensure that the edge-specific local parameters associated with each gene converge toward the gene’s shared consensus parameters, thereby maintaining consistency across the network.

In practical numerical implementation, the above problem is transformed into the augmented Lagrange function

19 $$\ell ={\sum}_{e\in {\mathcal E} }\left[{\ell }_{e}({\lambda }_{i,e},{\lambda }_{j,e},{w}_{e})+{\xi }_{i,e},{\lambda }_{i,e}-{\theta }_{i}+{\xi }_{j,e},{\lambda }_{j,e}-{\theta }_{j} +\frac{\rho }{2}{\Vert {\lambda }_{i,e}-{\theta }_{i}\Vert }^{2}+\frac{\rho }{2}{\Vert {\lambda }_{j,e}-{\theta }_{j}\Vert }^{2}\right],$$

where ${{{{\mathcal{l}}}}}_{e}({\lambda }_{i,e},{\lambda }_{j,e},{w}_{e})=-{{{\mathrm{ln}}}}\left[{{{\mathrm{Pr }}}}_{e}\left({\lambda }_{i,e},{\lambda }_{j,e},{{{\mathrm{w}}}}_{e};{{{\boldsymbol{x}}}}\right)\right]$, and ${\xi }_{i,e}$ is the Lagrange multiplier. Here, the first three equations form the Standard Lagrange function. For better convergence, we added a quadratic penalty parameter ρ to get the latter quadratic penalty terms. The scaled dual variable is used commonly ${u}_{i,e}={\xi }_{i,e}/\rho$, which is the scaled version of the Lagrange multiplier, providing numerical stability and convenience for computation. Therefore, we can get the scaled form of augmented Lagrange function

20 $$\,{\ell }_{s}={\sum}_{{{{\rm{e}}}}\in {\mathcal E} }\left[{\ell }_{e}({\lambda }_{i,e},{\lambda }_{j,e},{w}_{e})+\frac{\rho }{2}{\Vert {\lambda }_{i,e}-{\theta }_{i}+{u}_{i,e}\Vert }^{2}+\frac{\rho }{2}{\Vert {\lambda }_{j,e}-{\theta }_{j}+{u}_{j,e}\Vert }^{2}\right]+{{{\rm{const}}}}.$$

#### Initial values setting

Selecting appropriate initial values is essential for ensuring both the efficiency and stability of the estimation procedure. Here, we detail the initialization strategy below. The three groups of parameters involved in the model are initialized sequentially.

1. The node-level global consensus parameters ${\theta }_{i}=({\alpha }_{i},{\beta }_{i},{\phi }_{i})\in {{\mathbb{R}}}^{3},i\in \Omega$ and ${w}_{e}$. We use the single-gene parameters inferred from the classical ON-OFF model as the initial values ${\theta }_{i}^{{{{\rm{ini}}}}}=({\alpha }_{i}^{{{{\rm{ini}}}}},{\beta }_{i}^{{{{\rm{ini}}}}},{\phi }_{i}^{{{{\rm{ini}}}}})$ $(i\in \Omega )$. Given the observed counts ${X}_{i}=\left\{{x}_{{ik}},k=1,2,...{{\mbox{C}}}\right\}$, we solve the MLE optimization problem for each gene, respectively:
  21 $$\begin{array}{c}{\min }_{{\theta }_{i}^{{{{\rm{ini}}}}}}\left(-L\left({\theta }_{i}^{{{{\rm{ini}}}}}\right)\right)={\min }_{{\theta }_{i}^{{{{\rm{ini}}}}}}{\sum }_{k=1}^{C}-{{{\mathrm{ln}}}}Pr \left({X}_{{ik}}={x}_{{ik}};{\theta }_{i}^{{{{\rm{ini}}}}}\right),\end{array}$$
  where ${{{\mbox{Pr}}}}({X}_{{ik}}={x}_{{ik}};{\theta }_{i}^{{{{\rm{ini}}}}})$ obeys univariate Poisson–Beta distribution (Eq. 11). The negative log-likelihood is minimized using the scipy package in Python with the L-BFGS-B method with the initial values obtained by the moment estimators. Let ${{{\rm{E}}}}({X}_{i})$, ${{{\rm{E}}}}({X}_{i}^{2})$, ${{{\rm{E}}}}({X}_{i}^{3})$ $(i\in \Omega )$ be the mean, second-order raw moment, and third-order raw moment of gene-expression observed counts. The initial points ${\theta }_{i}^{{{{\rm{ini}}}}}=({\alpha }_{i}^{{{{\rm{ini}}}}},{\beta }_{i}^{{{{\rm{ini}}}}},{\phi }_{i}^{{{{\rm{ini}}}}})$ are obtained as the following results:
  22 $${\alpha }_{i}= 	 \frac{2{r}_{i1}\left({r}_{i3}-{r}_{i2}\right)}{{r}_{i1}{r}_{i2}-2{r}_{i1}{r}_{i3}+{r}_{i2}{r}_{i3}}, \\ {\beta }_{i}= 	 \frac{\left({r}_{i3}-{r}_{i2}\right)\left({r}_{i1}-{r}_{i3}\right)\left({r}_{i2}-{r}_{i1}\right)}{\left({r}_{i1}{r}_{i2}-2{r}_{i1}{r}_{i3}+{r}_{i2}{r}_{i3}\right)\left({r}_{i1}-2{r}_{i2}+{r}_{i3}\right)}, \\ {\phi }_{i}= 	 \frac{2{r}_{i1}{r}_{i3}-{r}_{i1}{r}_{i2}-{r}_{i2}{r}_{i3}}{{r}_{i1}-2{r}_{i2}+{r}_{i3}},$$
  where ${r}_{i1}={{{\rm{E}}}}({X}_{i})$, ${r}_{i2}=({{{\rm{E}}}}({X}_{i}^{2})-{{{\rm{E}}}}({X}_{i}))/{{{\rm{E}}}}({X}_{i})$, ${r}_{i3}=({{{\rm{E}}}}({X}_{i}^{3})-3{{{\rm{E}}}}({X}_{i}^{2})-2{{{\rm{E}}}}({X}_{i}))/({{{\rm{E}}}}({X}_{i}^{2})-{{{\rm{E}}}}({X}_{i}))$. To take variations in cell-specific factors $({\hat{c}}_{k}={\hat{\tau }}_{k}{\hat{s}}_{k})$ into consideration, we proposed a heuristic approach that uses ${x}_{{ik}}/{\hat{c}}_{k}$ instead of the raw ${x}_{{ik}}$ in the estimation of the moments. We set ${w}_{e}^{{{{\rm{ini}}}}}=0$ $(e{\in}{E})$.
2. The edge-specific local parameters ${\lambda }_{i,e}=({\alpha }_{i,e},{\beta }_{i,e},{\phi }_{i,e})\in {{\mathbb{R}}}^{3},i\in \Omega ,e\,{{{\mathscr{\in }}}}\,{{{\mathcal{E}}}}$. Here, the local variables are similarly initialized with the parameter values obtained from the single-gene inference, i.e., ${\lambda }_{i,e}^{{{{\rm{ini}}}}}={\theta }_{i}^{{{{\rm{ini}}}}}$.
3. The dual variables, ${u}_{i,e}={\lambda }_{i,e}-{\theta }_{i}\in {R}^{3},i\in \Omega ,e\,{{{\mathscr{\in }}}}\,{{{\mathcal{E}}}}$. All dual variables are initialized to zero vectors with the same dimensionality as ${\lambda }_{i,e}$.

#### Algorithmic optimization and iterative updates

The procedure for iteratively updating and optimizing the three primary variables is elaborated in the following section (Appendix Fig. S14A) (Hong et al, 2016; Mateos et al, 2010).

1. To update the local variables ${\lambda }_{i,e}$, $i\in \Omega$, $e{{{\mathscr{\in }}}}{{{\mathcal{E}}}}$, we perform edge-wise optimization in parallel. For each edge $e=(i,j){\in}{E}$, the local parameters ${\lambda }_{i,e}^{(k+1)}$, ${\lambda }_{j,e}^{(k+1)}$, and ${w}_{e}^{(k+1)}$ are obtained by solving the following minimization problem:
  23 $$\left({\lambda }_{i,e}^{(k+1)},{\lambda }_{j,e}^{(k+1)},{\omega }_{e}^{(k+1)}\right)= {{{{\rm{argmin}}}}}_{{\lambda }_{i,e},\,{\lambda }_{j,e},\,{\omega }_{e}}\left[ {\ell }_{e}({\lambda }_{i,e},{\lambda }_{j,e},{\omega }_{e})+\frac{\rho }{2}\Vert {\tilde{\lambda }}_{i,e}-{\tilde{z}}_{i}^{(k)}+{\tilde{u}}_{i,e}^{(k)}\Vert +\frac{\rho }{2}\Vert {\tilde{\lambda }}_{j,e}-{\tilde{z}}_{j}^{(k)}+{\tilde{u}}_{j,e}^{(k)}\Vert \right],$$
  where ${{{{\mathcal{l}}}}}_{e}(\cdot )$ denotes the edge-specific negative log-likelihood term. To improve stability, all parameters are scaled using the 5th-95th percentile range $({\widetilde{x}}_{{{{\rm{scaled}}}}}=x/({P}_{95}(x)-{P}_{05}(x)))$, which reduces the impact of extreme outliers. Since each edge can be updated independently, we employ joblib.Parallel in Python to compute all edge-wise updates concurrently, substantially improving computational efficiency (Friedman et al, 2008). Specifically, we employed MLE for each parallel optimization task. Noting that the core of the optimization in Eq. 23 involves evaluating the bivariate Poisson–Beta likelihood, whose computation is highly time-consuming. Since the solution of the univariate Poisson–Beta distribution ${Q}_{{\alpha }_{i},{\beta }_{i},{\phi }_{i}}({x}_{i})$ expressed as a form of hypergeometric function, we utilize the Gauss–Jacobi quadrature to obtain the approximated numerical solution of Poisson–Beta distribution:
  24 $${Q}_{{\alpha }_{i},{\beta }_{i},{\phi }_{i}}\left({x}_{i}\right)\approx {\sum }_{k=1}^{n}{a}_{k}^{\left(i\right)}{J}_{i}\left({\xi }_{k}^{\left(i\right)}\right),i=1,2,\ldots ,n,$$
  where ${a}_{k}^{(i)}$ and ${\xi }_{k}^{(i)}$ can be determined by the Jacobi polynomial J(⋅) with a certain degree.
2. To update the consensus variables ${\theta }_{i}$, $i\in \Omega$, we aggregate the edge-specific local parameters associated with each gene and apply a damped averaging scheme. For each node $i\in \Omega$, we first compute the provisional consensus value
  25 $${\theta }_{i,* }^{\left(k+1\right)}=\frac{1}{\left|{{{{\mathcal{E}}}}}_{i}\right|}{\sum}_{e\in {{{{\mathcal{E}}}}}_{i}}{\lambda }_{i,e}^{\left(k+1\right)},$$
  where ${{{{\mathcal{E}}}}}_{i}$ denotes the set of edges incident to node i. The consensus variable is then updated using a damping factor $\gamma \in (0,1]$:
  26 $${\theta }_{i}^{\left(k+1\right)}=\left(1-\gamma \right){\theta }_{i}^{\left(k\right)}+\gamma {\theta }_{i,* }^{\left(k+1\right)}.$$
  Here, ${\theta }_{i,* }^{(k+1)}$ represents the node-level consensus suggested by the current local updates, while the damping coefficient γ makes the update more conservative, enhances stability, and yields smoother convergence.
3. The dual variables ${u}_{i,e}$, $i\in \Omega$ $(e{\in}{E})$ are updated to record the discrepancy between the local variables and the consensus variables and to drive the algorithm toward consensus. For each edge $e=(i,j){\in}{E}$ and each associated node $i\in \Omega$, the scaled dual variable is updated as
  27 $${u}_{i,e}^{\left(k+1\right)}={u}_{i,e}^{\left(k\right)}+{\lambda }_{i,e}^{\left(k+1\right)}-{\theta }_{i}^{\left(k+1\right)}.$$
  This update increases the dual variable whenever the local variable ${\lambda }_{i,e}^{(k+1)}$ deviates from the node-level consensus ${\theta }_{i}^{(k+1)}$. As iterations proceed, the discrepancy is progressively reduced, promoting consistency between edge-specific parameters and their shared node-level values, and thereby facilitating the convergence of the ADMM algorithm.
4. To assess whether the algorithm has reached convergence, we monitor both the primal and dual residuals (Wang et al, 2019b). The primal residual ${r}^{(k)}={\lambda }^{(k)}-{\theta }^{(k)}$ measures the violation of the consensus constraint, quantifying how far the edge-specific local variables deviate from the node-level consensus. The dual residual ${s}^{(k)}=\rho \left({z}^{(k)}-{z}^{(k-1)}\right)$ measures the magnitude of change in the consensus variables between successive iterations, scaled by the penalty parameter ρ. ADMM is considered to have converged once both residual norms fall below their respective tolerances:

28 $${\Vert {r}^{(k)}\Vert }_{2}\le {\varepsilon }_{{{{\rm{pri}}}}},{\Vert {s}^{(k)}\Vert }_{2}\le {\varepsilon }_{{{{\rm{dual}}}}},$$

where ${\varepsilon }_{{{{\rm{abs}}}}}$ is the absolute tolerance (typically ${10}^{-4}$), ${\varepsilon }_{{{{\rm{rel}}}}}$ is the relative tolerance (typically ${10}^{-3}$), and n is the dimension of the consensus variable. Following standard ADMM practice, the tolerances are defined as

29 $${\varepsilon }_{{{{\rm{pri}}}}}= 	 \sqrt{n}{\varepsilon }_{{{{\rm{abs}}}}}+{\varepsilon }_{{{{\rm{rel}}}}}\cdot \max\{{\Vert A{x}^{(k)}\Vert }_{2},{\Vert B{z}^{(k)}\Vert }_{2},{\Vert c\Vert }_{2}\},\\ {\varepsilon }_{{{{\rm{dual}}}}}= 	 \sqrt{n}{\varepsilon }_{{{{\rm{abs}}}}}+{\varepsilon }_{{{{\rm{rel}}}}}\cdot {\Vert {A}^{T}{y}^{(k)}\Vert }_{2}.$$

Convergence is declared once both conditions are satisfied.

Finally, we performed a series of validation analyses to assess the contribution of the ADMM-based joint inference framework (Appendix Fig. S15). The results demonstrate that joint inference not only refines burst kinetic parameter estimation when regulatory coupling is present, but also recovers the joint and conditional expression structures required for regulatory interaction inference, which cannot be obtained from single-gene inference alone. By contrast, estimating burst kinetics and regulatory interactions sequentially risks absorbing regulatory coupling into intrinsic burst parameters and confounding regulatory inference with bursting-driven variability, both of which joint inference avoids by modeling both components simultaneously within a shared statistical framework.

#### Estimation of the cell-specific parameters

BurstLink explicitly incorporates the technical noise associated with scRNA-seq data, including capture efficiency ${\tau }_{k}$ and cell size ${s}_{k}$ for cell k (k=1,2,...,C). We introduce ${\hat{c}}_{k}={\hat{\tau }}_{k}{\hat{s}}_{k}$, which can simply be estimated using an appropriate cell-specific scaling factor from the raw data $({x}_{{ik}})$ (Eling et al, 2019). We posit that

30 $${\hat{c}}_{k}=\bar{c}\frac{{T}_{k}}{\bar{T}},$$

where ${T}_{k}={\sum}_{i}{x}_{{ik}}$ is the total number of counts, $\bar{T}={\sum}_{k}{\sum}_{i}{x}_{{ik}}$ is the average total count across cells. $\bar{c}$ is the average capture efficiency in the scRNA-seq protocol, which is set to specific values depending on the sequencing technology used (Klein et al, 2015). The single-cell dataset analyzed in this study was generated using the Smart-seq2 protocol, which exhibits a capture efficiency of around 30%. Accordingly, we set $\bar{c}$ to 0.3 (Svensson et al, 2017; Ziegenhain et al, 2017). We further performed sensitivity analyses on capture efficiency and cell size (Appendix Texts S1.5 and 1.6; Appendix Figs. S16 and 17).

#### Quantify gene–gene regulatory interactions based on RMI

Much effort has been devoted to inferring gene–gene interactions by using Pearson correlation or mutual information (Song et al, 2012). However, these methods can only reveal co-expression relationships, rather than directional regulatory relationships. Besides, it is challenging to yield accurate regulatory information using scRNA-seq data directly since the noise from raw experimental data (Harris et al, 2021). To address these challenges, we quantify regulatory interactions between two genes based on information theory (Krishnaswamy et al, 2014). Given the inferred analytical bivariate Poisson-Beta distribution $\hat{P}({x}_{i},{x}_{j})$ with inferred parameters, BurstLink infers regulatory interactions information from three perspectives (Appendix Fig. S14B).

1. Regulation visualization
  (i) Compute the inferred conditional probability $\hat{P}({x}_{i}| {x}_{j})$ and $\hat{P}({x}_{j}| {x}_{i})$ based on Eq. 12.
  (ii) Normalize $\hat{P}({x}_{i}{{{\rm{| }}}}{x}_{j})$ and $\hat{P}({x}_{j}{{{\rm{| }}}}{x}_{i})$ by rescaling the values of each column window
    31 $${\hat{P}}^{\ast}({x}_{i}=n\,|\,{x}_{j}=m)={\hat{P}}({x}_{i}=n\,|\,{x}_{j}=m)/{\max }_{k}\left\{{\hat{P}}({x}_{i}=k\,|\,{x}_{j}=m)\right\},\\ {\hat{P}}^{\ast }({x}_{j}=m\,|\,{x}_{i}=n)={\hat{P}}({x}_{j}=m\,|\,{x}_{i}=n)/{\max }_{k}\left\{{\hat{P}}({x}_{j}=k\,|\,{x}_{i}=n)\right\},$$
    and obtain the rescaled conditional probability ${\hat{P}}^{\ast }({x}_{i}{{{\rm{| }}}}{x}_{j})$ and ${\hat{P}}^{\ast }({x}_{j}{{{\rm{| }}}}{x}_{i})$.
  (iii) Visualize ${\hat{P}}^{\ast }$ as heatmaps.
2. Regulation types

(i) Compute the mass center of ${x}_{i}$ based on the rescaled conditional probability for each expression level ${x}_{j}$
  32 $$m({x}_{i})= 	 \, \frac{{\sum}_{{x}_{j}}{x}_{j}\tilde{P}\left({x}_{j}|{x}_{i}\right)}{{\sum}_{{x}_{j}}\tilde{P}\left({x}_{j}|{x}_{i}\right)},\\ \widetilde{P}({x}_{j}{{{\rm{| }}}}{x}_{i})\,\triangleq 	 \,\left\{\begin{array}{cc}\frac{{\hat{P}}^{* }\left({x}_{j}|{x}_{i}\right)}{\sum {\hat{P}}^{* }\left({x}_{j}|{x}_{i}\right)}, & {\hat{P}}^{* }\ge \varepsilon ,\\ 0, & {\hat{P}}^{* } < \,\varepsilon ,\end{array}\right.$$
  which serves as a summary statistic capturing monotonic shifts in the conditional distribution. Here, $\widetilde{P}({x}_{j}{{{\rm{| }}}}{x}_{i})\propto {\hat{P}}^{* }({x}_{j}{{{\rm{| }}}}{x}_{i})$ is the renormalized and denoised conditional probability.
(ii) Compute the Spearman rank correlation ρ and *p* value between the remaining expression levels ${x}_{i}$ and their conditional expectations $m({x}_{i})$. The sign and magnitude of ρ and *p* value are used to characterize the regulatory relationship (negative regulation if ρ>0 and *p* value <0.05, positive regulation if ρ<0 and *p* value <0.05, and nonregulation if *p* value >0.05).

(3) Regulation strengths

(i) Compute the reweighted joint distribution $\widetilde{P}({x}_{i},{x}_{j})$ based on the renormalized and denoised conditional probability $\widetilde{P}({x}_{j}{{{\rm{| }}}}{x}_{i})$,
  33 $$\widetilde{P}\left({x}_{i},{x}_{j}\right)=\widetilde{P}\left({x}_{j}|{x}_{i}\right)\times \widetilde{P}\left({x}_{i}\right).$$
  To ensure that all expression ranges of ${X}_{i}$ contribute equally, we impose a uniform marginal distribution over ${X}_{i}$, $\widetilde{P}({x}_{i})=1/K$, where K denotes the number of discrete expression states (bins) of ${X}_{i}$. Then the marginal probability $\widetilde{P}({x}_{j})$ can be obtained.
(ii) Calculate RMI based on the reweighted joint, marginal and conditional probabilities,

34 $$\begin{array}{c}{{{{\rm{RMI}}}}}_{{X}_{j}|{X}_{i}}={\sum} _{m}{\sum} _{n}\widetilde{P}\left({X}_{j}=m|{X}_{i}=n\right)\log \left[\frac{\tilde{P}\left({X}_{i}=n,{X}_{j}=m\right)}{\tilde{P}\left({X}_{i}=n\right)\tilde{P}\left({X}_{j}=m\right)}\right].\end{array}$$

By probability construction, RMI quantifies the intrinsic strength of statistical dependence between genes.

The same pipeline applies to compute ${{{{\rm{RMI}}}}}_{{X}_{i}|{X}_{j}}$ based on the rescaled conditional probability ${\hat{P}}^{* }({x}_{i}| {x}_{j})$, ensuring a bidirectional asymmetric assessment of regulatory strength.

Here, we provide a detailed explanation of the proposed framework. First, the regulation inference procedure is performed directly on the inferred analytical joint distribution, rather than on raw scRNA-seq observations, thereby avoiding the substantial noise and sampling variability inherent in single-cell data. Second, by visualizing the rescaled conditional probabilities ${\hat{P}}^{* }$, the resulting heatmaps represent an averaged regulatory response curve, characterizing how the expression level of one gene systematically changes in response to variations in another gene and revealing the underlying regulatory influence between gene pairs. Third, this approach captures the dominant directional trend of the conditional response curve, providing a robust and computationally efficient estimator of regulation type. Finally, regulatory strength is quantified using a reweighted information-entropy-based measure computed on a reconstructed probability space, which effectively captures the intrinsic asymmetry of bidirectional gene–gene interactions.

### Inference validation using synthetic scRNA-seq data

In this section, we showed the precision and robustness of inferred results (burst kinetics, regulation types, and strengths) for BurstLink. Given synthetic scRNA-seq data generated by known parameters ${\theta }_{{{{\rm{mech}}}}}=({k}_{{{{\rm{on}}}}}^{{{{\rm{X}}}}},{k}_{{{{\rm{on}}}}}^{{{{\rm{Y}}}}},{k}_{{{{\rm{off}}}}}^{{{{\rm{X}}}}},{k}_{{{{\rm{off}}}}}^{{{{\rm{Y}}}}},{k}_{{{{\rm{syn}}}}}^{{{{\rm{X}}}}},{k}_{{{{\rm{syn}}}}}^{{{{\rm{Y}}}}},{h}_{{{{\rm{X}}}}},{h}_{{{{\rm{Y}}}}},{r}_{{{{\rm{X}}}}},{r}_{{{{\rm{Y}}}}},{K}_{{{{\rm{X}}}}},{K}_{{{{\rm{Y}}}}},{\epsilon }_{{{{\rm{X}}}}},{\epsilon }_{{{{\rm{Y}}}}})$ via SSA, the corresponding estimated parameters ${\hat{\theta }}_{{{{\rm{stat}}}}}=({\hat{\alpha }}_{1},{\hat{\alpha }}_{2},{\hat{\beta }}_{1},{\hat{\beta }}_{2},{\hat{\phi }}_{1},{\hat{\phi }}_{2},\hat{w})$, the estimated burst kinetics parameters value (${{{\rm{BF}}}}_{1}$, ${{{{\rm{BF}}}}}_{2}$, ${{{{\rm{BS}}}}}_{1}$, ${{{{\rm{BS}}}}}_{2}$) and regulation types as well as strengths can be obtained through our inference framework. Firstly, we compared the distribution consistency between the synthetic scRNA-seq data under the settled mechanistic model parameters ${\theta }_{{{{\rm{mech}}}}}$ and the synthetic scRNA-seq data under the corresponding estimated parameters ${\hat{\theta }}_{{{{\rm{stat}}}}}$, which is assessed by the KS-test (Eq. 16). The results are illustrated in Fig. 2B. Secondly, to evaluate the precision of burst kinetics, we use the standard errors between the inferred burst kinetics and true burst kinetics for two genes as the evaluation metric:

35 $${{{\rm{Error}}}}\left({\theta}^{{{\rm{true}}}},{\theta }^{{{\rm{est}}}}\right)={\sum }_{i=1}^{2}\left({\log \left({{{\rm{BF}}}}_{i}^{{{\rm{true}}}}\right)-\log \left({{{\rm{BF}}}}_{i}^{{{\rm{est}}}}\right)}\right)^{2}+\left({\log \left({{{\rm{BS}}}}_{i}^{{{\rm{true}}}}\right)-\log \left({{{\rm{BS}}}}_{i}^{{{\rm{est}}}}\right)}\right)^{2},$$

where ${\theta }^{{{{\rm{true}}}}}=\left\{({{{{\rm{BF}}}}}_{i}^{{{{\rm{true}}}}},{{{{\rm{BS}}}}}_{i}^{{{{\rm{true}}}}}),i=1,2\right\}$ and ${\theta }^{{{{\rm{est}}}}}=\left\{({{{{\rm{BF}}}}}_{i}^{{{{\rm{est}}}}},{{{{\rm{BS}}}}}_{i}^{{{{\rm{est}}}}}),i=1,2\right\}$ denote the true and estimated burst kinetics (BF and BS), respectively (Fig. 3A). The chosen error metric uses logarithmic differences to normalize across scales, ensuring comparability between parameters with different magnitudes. Thirdly, we performed validation for the inferred regulation types and strengths. Since ${h}_{{{\mbox{X}}}}$ and ${h}_{{{\mbox{Y}}}}$ are the parameters related to feedback regulation in the mechanistic model (Eq. 14), the synthetic scRNA-seq data were generated by SSA under different ${h}_{{{\mbox{X}}}}$, ${h}_{{{\mbox{Y}}}}$ in terms of signs and sizes, implying that the two genes are regulated in various types and strengths. Then, we infer from such simulated data and check the inferred regulation types and strengths with the settled Hill coefficients ${h}_{{{\mbox{X}}}}$ and ${h}_{{{\mbox{Y}}}}$.

Furthermore, we explored the robustness of our statistical inference framework. For robustness of the cell numbers to the inference, we generate the synthetic scRNA-seq data with different numbers of cells (200, 300, 500, 1000, and 5000) for fixed parameter values in the cases of positive, negative feedback, and non-feedback regulation forms, respectively. Similarly, for the robustness of the missing data to the inference (sensitivity), we obtain the missing dataset by missing the complete data with a certain probability (0.1, 0.3, 0.5, 0.7, and 0.9). For different cell numbers and different levels of missing data, we run the inference process 50 times respectively and evaluate the validation of inference in terms of burst kinetics parameters (BF and BS) and parameters related to the regulation (w). The results are illustrated in Appendix Fig. S19.

### Benchmarking against existing GRNs inference methods

We also performed systematic benchmarking against current methods in terms of accuracy, robustness, scalability and unique methodological properties. Specifically, we compared BurstLink with several representative single-cell GRN inference methods, including PIDC (Chan et al, 2017), Phixer (Singh et al, 2019), GENIE3(Van Dijk et al, 2018), GRNBoost2 (Moerman et al, 2019), and SCENIC (Aibar et al, 2017). For benchmarking on biological data, we used the Smart-seq2 scRNA-seq dataset from mouse embryonic fibroblasts (C57 × CAST) (Larsson et al, 2019). In each benchmark realization, 30 genes were randomly sampled to construct a fully connected candidate regulatory subnetwork. To set up the ground truth through biologically plausible regulatory interactions, we employed scATAC-seq-derived TF–target priors (Kamimoto et al, 2023) as an ATAC-supported reference network for performance evaluation. To ensure robust evaluation, the entire benchmarking procedure was independently repeated 100 times using different randomly sampled gene subsets. The benchmarking results are summarized in Appendix Fig. S3. Moreover, we performed runtime scalability tests across different network sizes and cell numbers. The results showed that BurstLink remained computationally feasible for large subnetworks with favorable scalability, and that computational cost was determined primarily by the number of candidate gene pairs rather than the number of cells (Appendix Fig. S21).

### Data analysis

#### scRNA-seq data preprocessing

BurstLink is compatible with a broad range of scRNA-seq technologies and experimental designs, including plate-based Smart-seq2 and droplet-based 10x Genomics data. Here, we applied three scRNA-seq datasets to the BurstLink framework:

1. The processed scRNA-seq data from mouse embryonic fibroblasts for each allele (C57 × CAST) (Larsson et al, 2019). This scRNA-seq data is sequenced based on smart-seq2 technology (unique molecular identifier counts were used) and contains 10,727 genes and 224 individual cells on each allele, which has been widely used to investigate genome-wide transcriptional burst kinetics (Luo et al, 2023b). For quality control, we filtered out genes expressed in less than 50 cells and cells expressed in less than 2000 genes. Also, we removed genes whose overall average expression levels are less than 1. Under the assumption that the expression distributions of almost all genes for the CAST and C57 alleles follow similar shapes and the transcriptional dynamic behavior is consistent between alleles for most genes (Deng et al, 2014; Larsson et al, 2019), we merged the two allele expressions eventually to form a single-cell matrix consisting of 4311 genes and 448 cells.
  In this study, CAST and C57 allele-specific counts were concatenated to increase the effective sample size for downstream inference. This pooling strategy was based on the assumption that allele-specific expression distributions are largely conserved between the two alleles for most genes. To evaluate this assumption, we compared the expression distributions of CAST and C57 alleles using the Kolmogorov–Smirnov (KS) test. Among the genes retained for downstream analysis, 88.7% showed no significant difference between the two allele-specific distributions (Appendix Fig. S18A), supporting the validity of allele-level pooling. Importantly, this procedure increases the number of observations available for inference without directly altering gene expression levels. To further assess the reliability of BurstLink-inferred bursting kinetics, we compared the inferred BF and BS with the published allele-specific measurements reported by Larsson et al The inferred kinetic parameters correlate linearly with the published estimates (Appendix Fig. S18B–E), supporting the validity of the inferred bursting dynamics while enabling simultaneous inference of gene regulatory interactions within the same framework.
2. The processed scRNA-seq data from mouse ES cells for each allele (C57BL/6 × CAST/EiJ) (Larsson et al, 2019). This scRNA-seq dataset, generated using Smart-seq2 technology with unique molecular identifier counts, includes 10927 genes and 283 individual cells per allele. The preprocessing procedure is primarily the same as (1).
3. DMSO and IdU dataset. Desai et al conducted scRNA-seq data on mouse embryonic stem cells, treated with DMSO or 10 mM IdU for 24 h (Desai et al, 2021). Sequenced based on Illumina NovaSeq 6000, 12481 genes of transcriptomes from 812 cells and 13,780 genes of transcriptomes from 744 cells, respectively, are analyzed in this paper.

#### scATAC-seq data preprocessing

To narrow the scope of possible regulatory candidate genes in scRNA-seq data, we utilized the prior gene regulation information from two base-GRNs constructed by scATAC-seq data and motif scanning, respectively (Kamimoto et al, 2023). In that paper, both base-GRNs are presented as binary tables, including all the potential connections between TFs and their target genes, which are complementary to each other in case one of the databases is unavailable. Further, we extracted respectively bi-direction information for gene-pair interactions from pre-built base-GRNs of mouse (Cusanovich et al, 2015), and took the union of both, obtaining an integrated gene-wise interactions matrix with 8467306 pairs of genes (Appendix Fig. S4B).

We emphasize that scATAC-seq is used only to predefine a biologically plausible candidate regulatory space (Appendix Fig. S20) as a preprocessing step, with the gene-pair inference model itself relying solely on scRNA-seq count distributions. While BurstLink does not necessarily require scATAC-seq data, incorporating chromatin accessibility information, even from cell-type-matched but independently profiled datasets, improves the biological plausibility of the candidate TF–target regulatory space. Matched single-cell multi-omics data, when available, would provide additional benefit by reducing confounding from differences in cell state, batch effects, or sample composition, further enhancing the specificity of the inferred regulatory relationships.

#### Integration of selected base-GRN and filtered scRNA-seq data

Based on the selected gene–gene interactions matrix, we screened the gene-pair expression counts matrix from preprocessed scRNA-seq data, which ensures that inferred feedback regulation results can be benchmarked against the selected base-GRN (Appendix Fig. S4A,B,E). Finally, we obtained 291,228 gene pairs, of which 1225 pairs are bidirectional interactions, 290,003 pairs are unidirectional interactions.

### Downstream analysis

#### Visualization for UMAP embedding of the inferred gene regulatory interactions network with bursting

Using scRNA-seq data, genes are reduced to two dimensions with UMAP, where the 2D coordinates represent the positions within the inferred network. By integrating these coordinates with the inferred burst kinetics and regulatory information, we can visualize the UMAP embedding of the inferred gene regulatory interaction network, incorporating bursting dynamics, using the ggraph package in R, which is a weighted-directed network graph with weights representing the computed information flow.

#### Identification of signaling roles for genes using network centrality analysis

To discover the roles of genes (TF and TG), we quantitatively analyzed gene–gene regulatory interaction networks through graph theory approaches. According to graph theory, several centrality measures can be computed within a given signaling network, such as in-degree and out-degree. Having inferred gene–gene regulatory interaction types and strengths on a genome-wide scale, the gene–gene regulatory interaction network can be visualized.

In this network graph, we introduced two statistics: the regulated-interaction level and the regulating-interaction level. The regulated-interaction level, similar to in-degree, can be computed as the sum of the information flow of the incoming signaling from the overall gene group, indicating the extent to which the gene as a TG is regulated by other genes. The regulating-interaction level, similar to out-degree, can be computed as the sum of the information flow of the outgoing signaling to the overall gene group. By comparing the regulated-interaction and regulating-interaction levels for each gene, we can identify the gene roles. For a specific gene, if the regulated-interaction level is lower than the regulating-interaction level, this gene is regarded as a TF gene. Conversely, this gene is regarded as a TG gene.

#### Genome-wide impacts of regulatory interactions on burst kinetics

For the macro-level perspective, after inferring the burst kinetics (BF and BS) of each gene and the regulation types and strengths between them and their candidate TF genes in base-GRN, a regularized linear machine-learning model is built to assess how regulatory interactions modulate transcriptional burst kinetics under different gene regulation types (positive, negative, and nonregulation). Specifically, a multivariate linear regression is performed in low and high-expression level groups, respectively:

36 $${{{\rm{M}}}}={B}^{{{{\rm{M}}}}}\cdot X+{c}^{{{{\rm{M}}}}},$$

where the inputs ${{{\rm{M}}}}={({{{{\rm{M}}}}}_{1},{{{{\rm{M}}}}}_{2},\ldots ,{{{{\rm{M}}}}}_{N})}^{T}$ $({{\mbox{M}}}={{\mbox{BF}}},{{\mbox{BS}}},{{{\mbox{CV}}}}^{2})$ are bursting kinetics and expression variability vectors of all target genes, and $X={({x}_{1},{x}_{2},\ldots ,{x}_{N})}^{T}$ (${x}_{j}={{{{\rm{Regulation\; strength}}}}}_{j}\times {{{{\rm{Regulation\; type}}}}}_{j}$) is the independent variable matrix that includes inferred regulatory information from potential TF genes for each target gene. The outputs in this regression are coefficient matrices ${B}^{v}=({b}_{ij}^{v})_{N \times N}$ ($v={{{\rm{BF}}}},{{{\rm{BS}}}}$), where ${b}_{{ij}}^{v}=0$ if j=i, and the intercept matrices ${c}^{v}={({c}_{1}^{v},{c}_{2}^{v},\ldots ,{c}_{N}^{v})}^{T}$.

Here, the Bayesian ridge model provides the coefficient value as a posterior distribution, thus burst frequencies and sizes are regarded to follow a certain Normal distribution

37 $${{{\rm{M}}}}\sim {{{\rm{Normal}}}}\left({B}^{{{{\rm{M}}}}}\cdot X+{c}^{{{{\rm{M}}}}},{\sigma }^{{{{\rm{M}}}}}\right),$$

where ${B}^{{{{\rm{M}}}}}\sim {{{\rm{Normal}}}}({\mu }^{{{{\rm{M}}}}},{\sigma }^{{{{\rm{M}}}}})$, ${{{\rm{M}}}}=({{{\rm{BF}}}},{{{\rm{BS}}}},{{{{\rm{CV}}}}}^{2})$. For computational implementation, we use the Ridge class in the sklearn.linear_model with default parameters. The number of iterative calculations can be adjusted depending on the computational resources and available time.

Next, we make regression predictions for burst kinetics (BF and BS) and ${{{\mbox{CV}}}}^{2}$ based on the above model under different gene regulatory loops. Specifically, for a single target gene regulated by certain regulatory genes, these genes are all split into high or low-expression groups based on their genome-wide regulation strengths. The prediction results of burst kinetics and expression variability parameters under the above different regulation scenarios are presented in the form of a certain distribution. Here, we kept the regulation strength fixed at the inferred mean regulation strength for each gene and systematically increased the proportion of negative regulation (0.1, 0.3, 0.5, 0.7, and 0.9) among target genes. For each proportion, we performed 20 repeated experiments to generate the prediction results. Similarly, we increased the proportion of positive regulation (0.1, 0.3, 0.5, 0.7, and 0.9) and again conducted 20 repeated experiments for each proportion to obtain the corresponding predictions.

For the micro-level perspective, the overall regulation level can be defined as

38 $${{{\rm{OverallRegulationLevel}}}}={\sum}_{j}{{{{\rm{RegulationType}}}}}_{j}\times {{{{\rm{RMI}}}}}_{j}.$$

Based on mean-field theory, we consider all regulatory interactions of a specific gene within its regulatory loop as a unified effect. We then classify genes into positively or negatively regulated groups according to the sign of the overall regulation level. Finally, we perform *t*-tests between the two groups to assess how regulation type influences the bursting parameters and transcriptional noise.

## Supplementary information

Appendix

Peer Review File

Source data Fig. 2

Source data Fig. 3

Source data Fig. 4

Source data Fig. 5

Source data Fig. 6

## Data Availability

BurstLink code is open source and available on GitHub (https://github.com/LiyingZhou12/BurstLink/), along with detailed tutorials (https://burstlink.readthedocs.io/en/latest/). The source data of this paper are collected in the following database record: biostudies:S-SCDT-10_1038-S44320-026-00235-4.
