# Supplementary material for: Deciphering global transcriptional dynamics coordinated by gene-gene regulatory interactions using single-cell data
Source: Mol Syst Biol. 2026 Aug 3;22(11):1747–70. doi: 10.1038/s44320-026-00235-4 (PMC13728105; doi:10.1038/s44320-026-00235-4)
Supplement: Supplementary file 1 [file 44320_2026_235_MOESM1_ESM.pdf]

Appendix for:
Deciphering global transcriptional dynamics coordinated by gene-gene regulatory interactions using single-cell data

Table of Contents

S1 Appendix Text 3

S1.1 Gene-gene interaction with bursting statistical mechanistic model 3

S1.2 Genetic toggle switch model 4

S1.3 Regulatory interactions inferred from snapshot data 5

S1.4 Robustness of BurstLink to transcript-to-regulatory-input scaling and protein-mediated regulation 7

S1.5 Sensitivity analysis on capture efficiency 7

S1.6 Sensitivity analysis on cell size 9

S1.7 Computational scalability analysis of BurstLink 9

S2 Appendix Figures 10

Appendix Figure S1. 10

Appendix Figure S2. 10

Appendix Figure S3. 11

Appendix Figure S4. 12

Appendix Figure S5. 13

Appendix Figure S6. 14

Appendix Figure S7. 15

Appendix Figure S8. 16

Appendix Figure S9. 16

Appendix Figure S10. 17

Appendix Figure S11. 18

Appendix Figure S12. 19

Appendix Figure S13. 19

Appendix Figure S14. 20

Appendix Figure S15. 21

Appendix Figure S16. 23

Appendix Figure S17. 23
Appendix Figure S18. 24
Appendix Figure S19. 25
Appendix Figure S20. 26
Appendix Figure S21. 26
S3 Appendix Tables..27
Appendix Table S1. 27
Appendix Table S2. 27
Appendix Table S3. 27
Appendix Table S4. 29
References..30

S1 Appendix Text

S1.1 Gene-gene interaction with bursting statistical mechanistic model

Bivariate Poisson–Beta distribution

In our statistical mechanistic framework for inferring coupled dynamics of regulatory interactions and transcriptional bursting, the bivariate Poisson-Beta distribution serves as a crucial core to establish a sensible bridge between the expressions of two individual genes, thus capturing the regulatory interactions and burst kinetics simultaneously. Here we offer a more detailed interpretation of the Poisson-Beta distribution based on the Sarmanov-Lee distribution (Sarabia & Gomez-Deniz, 2011). Under the assumption that the abundances of mRNA and protein are highly dependent (Liu et al, 2016), any two steady-state mRNA numbers $\mathrm{X} = (X_i, X_j) = \{(X_{ik}, X_{jk}), k = 1,2,\ldots,\mathrm{C}\}$ follow a bivariate Poisson-Beta distribution and the joint probability mass function can be written as

$$\Pr(X_{ik} = x_{ik}, X_{jk} = x_{jk}) = \int_{[0,1]^2} \prod_{l=(i,j)} \frac{e^{-\phi_l \tau_k s_k \eta_l} (\phi_l \tau_k s_k \eta_l)^{x_{lk}}}{x_{lk}!} L(\eta_i, \eta_j)\, d\eta_i d\eta_j, \tag{S1}$$

where $L(\eta_i, \eta_j) = f_i(\eta_i) f_j(\eta_j)\left[1 + w\, h_i(\eta_i) h_j(\eta_j)\right]$ is the Sarmanov–Lee distribution used to construct a flexible two-dimensional distribution with Beta marginals (Hernández-Bastida & Fernández-Sánchez, 2012). In our case, we considered the above Sarmanov-Lee distribution

$$\begin{aligned} f_i &\sim \mathrm{Beta}(\alpha_i, \beta_i), \\ h_i(\eta_i) &= \eta_i - \left[\frac{\alpha_i}{\alpha_i + \beta_i}\right], \quad \forall i \in \Omega, \end{aligned} \tag{S2}$$

where w is a real number such that $1 + w\, h_i(\eta_i) h_j(\eta_j) \geq 0\ \forall (\eta_i, \eta_j) \in [0,1]^2$, with satisfying

$$-\frac{(\alpha_i + \beta_i)(\alpha_j + \beta_j)}{\max\{\alpha_i \alpha_j, \beta_i \beta_j\}} \leq w \leq \frac{(\alpha_i + \beta_i)(\alpha_j + \beta_j)}{\max\{\alpha_i \beta_j, \alpha_j \beta_i\}}. \tag{S3}$$

Calculation for the related statistics

(1) Correlation coefficient

$$\mathrm{Corr}(X_i, X_j) = w \times \prod_{l=(i,j)} \frac{\phi_l \alpha_l \beta_l}{(\alpha_l + \beta_l)\sqrt{\alpha_l + \beta_l + 1}\sqrt{\alpha_l \phi_l (\alpha_l + \beta_l)(\alpha_l + \beta_l + 1) + \alpha_l \beta_l \phi_l^2}}, \tag{S4}$$

(2) Mutual information

$$\mathrm{MI} = \sum_m \sum_n \Pr(X_i = n, X_j = m) \log\left[\frac{\Pr(X_i = n, X_j = m)}{Q_{\alpha_i,\beta_i,\phi_i}(n)\, Q_{\alpha_j,\beta_j,\phi_j}(m)}\right], \tag{S5}$$

where $Q_{\alpha_i,\beta_i,\phi_i}(\cdot)$ is the univariate Poisson–Beta probability mass function in Eq. 11 of the main text.

Summary for statistical mechanistic model

Here, we summarized the statistical mechanistic model in two aspects. On the one hand, this model enables the depiction of the transcriptional burst kinetics for a gene-pair loop, i.e., the model can describe the switching of the gene promoter states (inactive and active states). Considering that the classical telegraph model offers a rational mechanistic interpretation of single-gene transcriptional burst (Cao & Grima, 2020; Peccoud & Ycart, 1995) and Poisson-Beta distribution has been proved to be the steady-state expression distribution (Kim & Marioni, 2013; Vu et al, 2016), the marginal distribution of gene-pair expression distribution is configured as Poisson-Bata distribution. On the other hand, the interactions between two

genes can be embedded in this model. Thus, we established the statistical dependence relationship between the gene pair by introducing the Sarmanov-Lee distribution given the marginal Poisson-Beta distribution. Consequently, the bivariate Poisson-Beta statistical model we proposed can interpret gene-gene interaction in a transcriptional bursting manner.

Gibbs sample algorithm

In this section, the Gibbs sample algorithm based on the bivariate Poisson–Beta distribution to generate synthetic data is presented as follows:

(i) Input model parameters $\theta_{\text{stat}} = (\alpha_1, \alpha_2, \beta_1, \beta_2, \phi_1, \phi_2, w)$, the sampling threshold N, and the number of samples M,

(ii) Inverse sampling based on conditional probabilities,

(iii) Initialize the state values $(x_1^{(1)}, x_2^{(1)})$ randomly, and sample by step (ii). For an iteration step from j to $j+1$, sample from conditional probabilities $\Pr(x_2 \mid x_1^{(j)})$ and yield $x_2^{(j+1)}$, then sample from conditional probabilities $\Pr(x_1 \mid x_2^{(j+1)})$ and yield $x_1^{(j+1)}$,

(iv) Output the steady sample $\left\{(x_1^{(N+1)}, x_2^{(N+1)}), (x_1^{(N+2)}, x_2^{(N+2)}), \ldots, (x_1^{(N+M-1)}, x_2^{(N+M-1)})\right\}$ obtained in step (iii).

Here we note that if generating two genetically independent data, the library function of Python scipy can be utilized to generate two independent sets of Poisson–Beta random numbers without going through the Gibbs sampling method.

S1.2 Genetic toggle switch model

Given the statistical inference framework, there is an important issue raising: whether our statistical model can be mutually accountable with a mechanistic model in the biochemical reaction process. Therefore, we discussed a common gene-expression model, the genetic toggle switch model (Tian & Burrage, 2006) that can simultaneously characterize feedback regulations and transcriptional burst kinetics in a gene-pair loop.

$$\begin{gathered}
\mathrm{OFF}_X \xrightarrow{k_{on,reg}^X(m)} \mathrm{O}N_X, \mathrm{ON}_X \xrightarrow{k_{off}^X} OFF_X, \mathrm{ON_X} \xrightarrow{k_{syn}^X} \mathrm{O}N_X + X, \\
\mathrm{OFF}_Y \xrightarrow{k_{on,reg}^Y(n)} \mathrm{O}N_Y, \mathrm{ON}_Y \xrightarrow{k_{off}^Y} OFF_Y, \mathrm{ON_Y} \xrightarrow{k_{syn}^Y} \mathrm{O}N_Y + Y, \\
X \xrightarrow{k_{deg}^X} \emptyset, Y \xrightarrow{k_{deg}^Y} \emptyset,
\end{gathered} \tag{S6}$$

where the relevant parameters have been clarified in the main text. Let $P(n,m,t) = [P_{(\text{off,off})}^{X,Y}(n,m,t),\ P_{(\text{off,on})}^{X,Y}(n,m,t),\ P_{(\text{on,off})}^{X,Y}(n,m,t),\ P_{(\text{on,on})}^{X,Y}(n,m,t)]^{\mathrm{T}}$ be the probability vector that there are n mRNA transcripts for X and m mRNA transcripts for Y at time t for different promoter states, respectively. For example, $P_{(\text{on,off})}^{X,Y}(n,m,t)$ represents the probability when the corresponding encoding gene promoter for X stays at ON state and that for Y stays at OFF state at time t, respectively. The evolution of the above mechanism model is governed by the following set of chemical master equations (CMEs)

$$\begin{aligned}
\frac{\partial P_{(\text{off,off})}^{X,Y}(n,m,t)}{\partial t} &= k_{\text{off}}^X P_{(\text{on,off})}^{X,Y}(n,m,t) + k_{off}^Y P_{(\text{off,on})}^{X,Y}(n,m,t) \\
&\quad + k_{\text{syn}}^X\left[(n+1)P_{(\text{off,off})}^{X,Y}(n+1,m,t) - nP_{(\text{off,off})}^{X,Y}(n,m,t)\right]
\end{aligned}$$

$$
\begin{aligned}
&+k_{\mathrm{syn}}^{Y}\left[(m+1)P_{(\mathrm{off,off})}^{X,Y}(n,m+1,t)-mP_{(\mathrm{off,off})}^{X,Y}(n,m,t)\right]\\
&-\left(\frac{k_{\mathrm{on}}^{X}}{(K_s^X)^h+(r_c m)^h}+\varepsilon_X\right)P_{(\mathrm{off,off})}^{X,Y}(n,m,t),
\end{aligned}
$$

$$
\begin{aligned}
\frac{\partial P_{(\mathrm{on,off})}^{X,Y}(n,m,t)}{\partial t}=&\left(\frac{k_{\mathrm{on}}^{X}}{(K_s^X)^h+(r_c m)^h}+\varepsilon_X\right)P_{(\mathrm{off,off})}^{X,Y}(n,m,t)\\
&-\left(k_{\mathrm{off}}^{X}+\frac{k_{\mathrm{on}}^{Y}}{(K_s^Y)^h+(r_c n)^h}+\varepsilon_Y\right)P_{(\mathrm{on,off})}^{X,Y}(n,m,t)\\
&+k_{\mathrm{syn}}^{X}\left[(n+1)P_{(\mathrm{on,off})}^{X,Y}(n+1,m,t)-nP_{(\mathrm{on,off})}^{X,Y}(n,m,t)\right]\\
&+k_{\mathrm{syn}}^{Y}\left[(m+1)P_{(\mathrm{on,off})}^{X,Y}(n,m+1,t)-mP_{(\mathrm{on,off})}^{X,Y}(n,m,t)\right],
\end{aligned}
$$

$$
\begin{aligned}
\frac{\partial P_{(\mathrm{off,on})}^{X,Y}(n,m,t)}{\partial t}=&\left(\frac{k_{\mathrm{on}}^{Y}}{(K_s^Y)^h+(r_c n)^h}+\varepsilon_Y\right)P_{(\mathrm{off,off})}^{X,Y}(n,m,t)\\
&-\left(\frac{k_{\mathrm{on}}^{X}}{(K_s^X)^h+(r_c m)^h}+\varepsilon_X+k_{\mathrm{off}}^{Y}\right)P_{(\mathrm{off,on})}^{X,Y}(n,m,t)\\
&+k_{\mathrm{syn}}^{X}\left[(n+1)P_{(\mathrm{off,on})}^{X,Y}(n+1,m,t)-nP_{(\mathrm{off,on})}^{X,Y}(n,m,t)\right]\\
&+k_{\mathrm{syn}}^{Y}\left[(m+1)P_{(\mathrm{off,on})}^{X,Y}(n,m+1,t)-mP_{(\mathrm{off,on})}^{X,Y}(n,m,t)\right],
\end{aligned}
$$

$$
\begin{aligned}
\frac{\partial P_{(\mathrm{on,on})}^{X,Y}(n,m,t)}{\partial t}=&\left(\frac{k_{\mathrm{on}}^{X}}{(K_s^X)^h+(r_c m)^h}+\varepsilon_X\right)P_{(\mathrm{off,on})}^{X,Y}(n,m,t)\\
&+\left(\frac{k_{\mathrm{on}}^{Y}}{(K_s^Y)^h+(r_c n)^h}+\varepsilon_Y\right)P_{(\mathrm{on,off})}^{X,Y}(n,m,t)\\
&-\left(k_{\mathrm{off}}^{X}+k_{\mathrm{off}}^{Y}\right)P_{(\mathrm{on,on})}^{X,Y}(n,m,t)\\
&+k_{\mathrm{syn}}^{X}\left[(n+1)P_{(\mathrm{on,on})}^{X,Y}(n+1,m,t)-nP_{(\mathrm{on,on})}^{X,Y}(n,m,t)\right]\\
&+k_{\mathrm{syn}}^{Y}\left[(m+1)P_{(\mathrm{on,on})}^{X,Y}(n,m+1,t)-mP_{(\mathrm{on,on})}^{X,Y}(n,m,t)\right],\quad \text{(S7)}
\end{aligned}
$$

where $k_{\mathrm{on}}^{X}=k_s^X\cdot\varepsilon_X, k_{\mathrm{on}}^{Y}=k_s^Y\cdot\varepsilon_Y$ are the basic switch rates, the regulation of promoter activation/inactivation is characterized by a Hill function. K_s denotes the dissociation constant, h is the Hill coefficient: positive feedback when $h<0$, negative feedback when $h>0$, none feedback when $h=0$. The effective regulatory input is a monotonic function of regulator mRNA abundance and use the simplest first-order approximation, $r_{\mathrm{X}}\cdot X$ and $r_{\mathrm{Y}}\cdot Y$, where r_{X} and r_{Y} are scaling coefficients. Although analytic solutions are infeasible, steady-state distributions can be obtained via SSA (Gillespie, 1976): $P(n,m)=\lim_{t\to\infty}\left[P_{(\mathrm{off,off})}^{X,Y}(n,m,t)+P_{(\mathrm{off,on})}^{X,Y}(n,m,t)+P_{(\mathrm{on,off})}^{X,Y}(n,m,t)+P_{(\mathrm{on,on})}^{X,Y}(n,m,t)\right]$. The validation of our statistical inference framework using synthetic data in the main text is achieved by generating simulated data via this algorithm.

S1.3 Regulatory interactions inferred from snapshot data

Here, we provide an intuitive explanation of how directional regulatory information can arise from snapshot data. We added a minimal analytical example based on a simple two-gene regulatory module. Given a two-gene regulatory module $X\to Y$, gene X is the upstream regulator and gene Y is the

downstream target gene. Conditional on the expression level of the upstream regulator $X = m$, the downstream gene Y follows a two-state transcriptional bursting model:

$$G_0 \xrightarrow{k_{on}(m)} G_1, G_1 \xrightarrow{k_{off}} G_1$$
$$G_1 \overset{s}{\to} G_1 + Y, Y \overset{\gamma}{\to} \phi. \tag{S8}$$

Here, G_0 and G_1 denote the OFF and ON promoter states, respectively. The parameter s is the transcription rate and γ is the mRNA degradation rate. The key assumption is that the upstream regulator modulates the burst activation rate of the downstream gene $k_{on}(m)$. If $k_{on}(m)$ is an increasing function of m, then X positively regulates Y; if it is decreasing, X negatively regulates Y.

Let $P_0(n, t \mid m)$ and $P_1(n, t \mid m)$ denote the probabilities that gene Y contains n mRNA molecules while its promoter is in the OFF and ON states, respectively. The corresponding conditional chemical master equations can be written as

$$\begin{aligned} \frac{dP_0(n,t \mid m)}{dt} &= -k_{\mathrm{on}}(m)P_0(n,t \mid m) + k_{\mathrm{off}}P_1(n,t \mid m) \\ &\quad + \gamma[(n+1)P_0(n+1,t \mid m) - nP_0(n,t \mid m)], \\ \frac{dP_1(n,t \mid m)}{dt} &= k_{\mathrm{on}}(m)P_0(n,t \mid m) - k_{\mathrm{off}}P_1(n,t \mid m) \\ &\quad + s[P_1(n-1,t \mid m) - P_1(n,t \mid m)] \\ &\quad + \gamma[(n+1)P_1(n+1,t \mid m) - nP_1(n,t \mid m)]. \end{aligned} \tag{S9}$$

By introducing the probability generating functions and solving the steady-state master equation, the total generating function can be obtained

$$F(z \mid m) = {}_1F_1!\left(\alpha(m); \alpha(m) + \beta; \phi(z-1)\right), \tag{S10}$$

where $\alpha(m) = k_{\mathrm{on}}(m)/\gamma$, $\beta = k_{\mathrm{off}}/\gamma$, $\phi = s/\gamma$. Using the integral representation of the Kummer function, the total generating function can be written as

$$F(z \mid m) = \int_0^1 \exp\{\phi\eta(z-1)\} \frac{\eta^{\alpha(m)-1}(1-\eta)^{\beta-1}}{\mathrm{Beta}(\alpha(m),\beta)} d\eta\,. \tag{S11}$$

Note that $\exp\{\phi\eta(z-1)\}$ is the probability generating function of a Poisson distribution with mean $\phi\eta$. Therefore, the above expression can be interpreted as a Poisson distribution whose rate parameter is mixed by a Beta distribution, implying that the steady-state distribution follows a Poisson-Beta form

$$Y \mid \eta, X = m \sim \mathrm{Poisson}(\phi\eta)$$
$$\eta \mid X = m \sim \mathrm{Beta}(\alpha(m), \beta) \tag{S12}$$

or equivalently,

$$P(Y = n \mid X = m) = \int_0^1 \frac{(\phi\eta)^n e^{-\phi\eta}}{n!} \frac{\eta^{\alpha(m)-1}(1-\eta)^{\beta-1}}{\mathrm{Beta}(\alpha(m),\beta)} d\eta. \tag{S13}$$

This result shows that the upstream regulator directly changes the steady-state conditional distribution of the downstream gene through $\alpha(m)$. Thus, the conditional mean is

$$\mathrm{E}[Y \mid X = m] = \phi\, \mathrm{E}[\eta \mid X = m] = \frac{s}{\gamma} \frac{k_{\mathrm{on}}(m)}{k_{\mathrm{on}}(m) + k_{\mathrm{off}}}. \tag{S14}$$

Differentiating with respect to m gives

$$\frac{d\,\mathrm{E}[Y \mid X = m]}{dm} = \frac{s\,k_{\mathrm{off}}}{\gamma\,(k_{\mathrm{on}}(m) + k_{\mathrm{off}})^2} k'_{\mathrm{on}}(m). \tag{S15}$$

Since $k_{\mathrm{on}}(m)$ is assumed to be monotonic, the sign of the derivative is determined by the sign of $k'_{\mathrm{on}}(m)$. Therefore, changes in the upstream gene expression systematically alter the stationary expression distribution of the downstream gene. Importantly, this analytical example demonstrates that directional regulation enters the generative process asymmetrically $X \xrightarrow{k_{\mathrm{on}}(m)} Y$. This result explains why directional information can be retained in snapshot data, under the assumption that the observed cells are samples from an underlying steady-state distribution, despite the absence of temporal measurements.

S1.4 Robustness of BurstLink to transcript-to-regulatory-input scaling and protein-mediated regulation

Firstly, we performed a sensitivity analysis by systematically varying r_{X} over a 16-fold dynamic range (1.75–28), generating synthetic data using the toggle-switch model SSA, and re-running the full BurstLink inference pipeline. Across the entire range examined, the recovery rate remained consistently high for both regulation types (Fig. S13, 0.80–0.94 for positive and 0.78–0.96 for negative regulation). These results demonstrate that the recovery of regulation direction and type is robust to the specific value of r_{X}.

Second, we extended the toggle-switch model to include the TF's protein species, so that regulation is mediated by protein rather than by mRNA, with protein degradation rate = 1/4 of the mRNA degradation rate (Schwanhäusser et al, 2011). This explicitly introduces the buffering and time delay between transcript and regulatory activity. We then compared inference under this protein-delay model against the original model, for both positive (Fig. S11A–G) and negative (Fig. S11H–N) regulation. As expected, the protein trajectories are slower and buffered relative to mRNA (Fig. S11C, J). Nevertheless, BurstLink still correctly recovers the qualitative regulatory relationship — both the type (positive/negative) and the direction — under the protein-delay model: the rescaled conditional-probability heatmaps continue to display the correct positive (F, G) or negative (M, N) trend with and without protein delay. While slow protein turnover attenuates the inferred coupling relative to the instantaneous case, it does not abolish all the regulatory signal in the stationary pairwise mRNA distribution, supporting the use of regulator mRNA as a proxy for recovering the type and direction of regulation even when the true mediator is a long-lived protein.

S1.5 Sensitivity analysis on capture efficiency

We examined the effect of sampling noise on Poisson–Beta-based burst kinetic inference through theoretical analysis and systematic benchmarking simulations. First, the bivariate Poisson-Beta model remains closed under independent binomial sampling. Specifically, let the true transcript counts (X_i, X_j) of any two genes be

$$X_i \mid \eta_i \sim \mathrm{Poisson}(\phi_i \eta_i), \quad X_j \mid \eta_j \sim \mathrm{Poisson}(\phi_j \eta_j), \quad \forall i, j \in \{1,2,\dots,n\}, \tag{S16}$$

where (η_i, η_j) follow the joint beta-type latent distribution used in the bivariate Poisson–Beta model. Here, we set $(\eta_i, \eta_j) \sim f_{\mathrm{Beta}}(\alpha_i, \beta_i, \alpha_j, \beta_j, \phi_i, \phi_j, w_{ij})$. If the observed counts (Y_i, Y_j) are generated by independent binomial sampling,

$$Y_i \mid X_i \sim \mathrm{Binomial}(X_i, c_i), \quad Y_j \mid X_j \sim \mathrm{Binomial}(X_j, c_j), \tag{S17}$$

by marginalizing over the unobserved true count X_i, we obtain

$$P(Y_i = y \mid \eta_i) = \sum_{x=y}^{\infty} P(Y_i = y \mid X_i = x) P(X_i = x \mid \eta_i)$$

$$= \sum_{x=y}^{\infty} \binom{x}{y} c_i^y (1 - c_i)^{x-y} \frac{e^{-\phi_i \eta_i} (\phi_i \eta_i)^x}{x!}. \tag{S18}$$

Let $m = x - y$, then

$$P(Y_i = y \mid \eta_i) = \frac{c_i^y (\phi_i \eta_i)^y}{y!} e^{-\phi_i \eta_i} \sum_{m=0}^{\infty} \frac{[(1 - c_i)\phi_i \eta_i]^m}{m!}$$

$$= \frac{(c_i \phi_i \eta_i)^y}{y!} e^{-\phi_i \eta_i} e^{(1-c_i)\phi_i \eta_i} = \frac{(c_i \phi_i \eta_i)^y}{y!} e^{-c_i \phi_i \eta_i} \tag{S19}$$

Thus,

$$Y_i \sim \text{Poisson}(c_i \phi_i \eta_i), \quad Y_j \sim \text{Poisson}(c_j \phi_j \eta_j). \tag{S20}$$

After integrating over the same joint latent distribution of (η_i, η_j), we obtain

$$(Y_i, Y_j) \sim \text{Bivariate Poisson-Beta}(\alpha_i, \beta_i, \alpha_j, \beta_j, c_i \phi_i, c_j \phi_j, w_{ij}). \tag{S21}$$

Therefore, under independent binomial sampling with constant capture efficiencies, the bivariate Poisson–Beta family is closed. The Beta shape parameters and the dependency parameter are preserved, whereas the ϕ_i are rescaled to $c_i \phi_i$.

Second, we performed a systematic benchmarking analysis to assess the robustness of BurstLink under sampling noise introduced during sequencing. As illustrated in Fig. S16A, we modeled transcript capture as an independent binomial sampling process with capture efficiency c. We constructed a synthetic example to evaluate the practical impact of sampling noise. Specifically, we first generated the ground-truth expression distribution from the underlying transcriptional bursting model from SSA simulator (Fig. S16B) and then applied binomial downsampling to obtain the observed counts (Fig. S16C), thereby mimicking transcript loss during sequencing. BurstLink was subsequently applied to the downsampled observations. We evaluated the robustness of BurstLink at three complementary levels: (1) whether BurstLink recovers the underlying biological distribution from the observed noisy data; (2) whether the inferred parameters were sufficient to reconstruct the original true distribution; (3) whether the underlying burst kinetic parameters, including burst frequency (BF) and burst size (BS), are accurately estimated despite the presence of sampling noise. As shown in Fig. S16D-G, BurstLink recovered the underlying expression distributions despite substantial sampling noise, and the inferred burst kinetic parameters remained consistent with their corresponding ground-truth values. In addition, BurstLink demonstrates robustness to sampling noise over a range of capture efficiencies (Fig. S16AH-J).

Furthermore, we performed sensitivity analysis using real scRNA-seq data. We applied binomial downsampling with different capture efficiencies and compared the burst parameters inferred from downsampled data with those inferred from the original data. As shown in Fig. S16AK-N, burst frequency and burst size estimates remained highly correlations across different downsampling levels, indicating that BurstLink-derived bursting kinetics are stable under sampling perturbations.

S1.6 Sensitivity analysis on cell size

In this section, we preformed sensitivity analysis on cell size. Specifically, to mimic the effect of cell-size variability on observed transcript counts, we estimated an empirical cell-size factor distribution from a real scRNA-seq dataset using per-cell library size, denoted as $s_k \sim \hat{P}(s)$ (Fig. S17A). The observed count X_{ik} for gene i in cell k was then sampled as

$$X_{ik} \sim \text{Poisson}(s_k \phi_i \eta_i), \quad \eta_i \sim \text{Beta}(\alpha_i, \beta_i), \quad i = 1,2,\dots,n \tag{S22}$$

where s_k denotes the cell-size factor. This treatment for cell size is consistent with the recent scRNA-seq burst-inference model (Tang et al, 2023), which explicitly represents cell size as a cell-specific multiplicative factor affecting the effective transcription rate and observed transcript-count distribution.

This procedure produced an observed distribution that differed from the original true distribution because of cell-size-associated extrinsic variability (Fig. S17B). We then applied BurstLink to the observed distribution and inferred the corresponding bursting parameters. Based on the inferred parameters, we reconstructed the observation-space distribution (Fig. S17C). We used the Kolmogorov–Smirnov test to assess whether the inferred distribution was statistically consistent with the target distribution. The results show that BurstLink accurately recovered both burst frequency and burst size under this cell-size perturbation setting (Fig. S17D). To further evaluate robustness, we repeated this simulation independently 100 times. In more than 90% of the simulations, the recovered distributions were statistically consistent with the corresponding target distribution (Fig. S17E). In addition, the recovered bursting kinetic parameters showed low error across repeated simulations (Fig. S17F). Together, these results indicate that BurstLink remains robust when cell-size-associated extrinsic noise is explicitly introduced into the synthetic benchmarking procedure.

S1.7 Computational scalability analysis of BurstLink

For the full mouse embryonic fibroblast (MEF) dataset containing 4,311 genes and 291,228 candidate gene pairs, the complete BurstLink analysis required approximately 14.2 h using 8 CPU cores and was reduced to approximately 7.8 h on a 16-core workstation. These results demonstrate the favorable parallel scalability of BurstLink and indicate that computational efficiency can be substantially improved with additional computing resources.

For the gene-number benchmark, we randomly sampled subnetworks containing 20, 50, 100, 500, and 1,000 genes from the MEF dataset. For each network size, the sampling and inference procedures were repeated 50 times, and the runtime distributions were recorded (Fig. S21A). For the cell-number benchmark we generated synthetic datasets using stochastic simulation algorithm (SSA) simulations and randomly sampled 500, 1,000, 2,000, and 5,000 cells from the simulated data. For each cell number, the sampling and inference procedures were repeated 50 times, and the runtime distributions were recorded (Fig. S21B). Together, BurstLink remained computationally feasible for large subnetworks, demonstrating favorable scalability with respect to network size. Runtime is substantially more sensitive to network size than to cell number, indicating that the number of candidate gene pairs is the primary determinant of computational cost.

S2 Appendix Figures

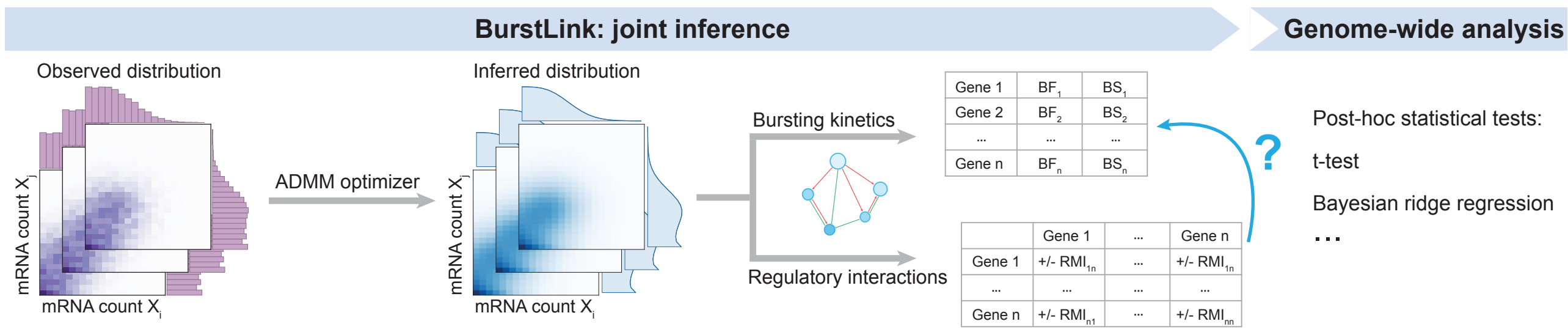

Appendix Figure S1. Overview of the BurstLink framework. Schematic illustration of the overall analysis pipeline. BurstLink first jointly infers transcriptional bursting parameters and gene regulatory interactions from observed single-cell gene expression distributions using an ADMM-based optimization framework. The inferred bursting and regulatory parameters are then used for downstream genome-wide analyses, including statistical modeling and hypothesis testing, to identify global relationships between transcriptional bursting dynamics and regulatory interactions.

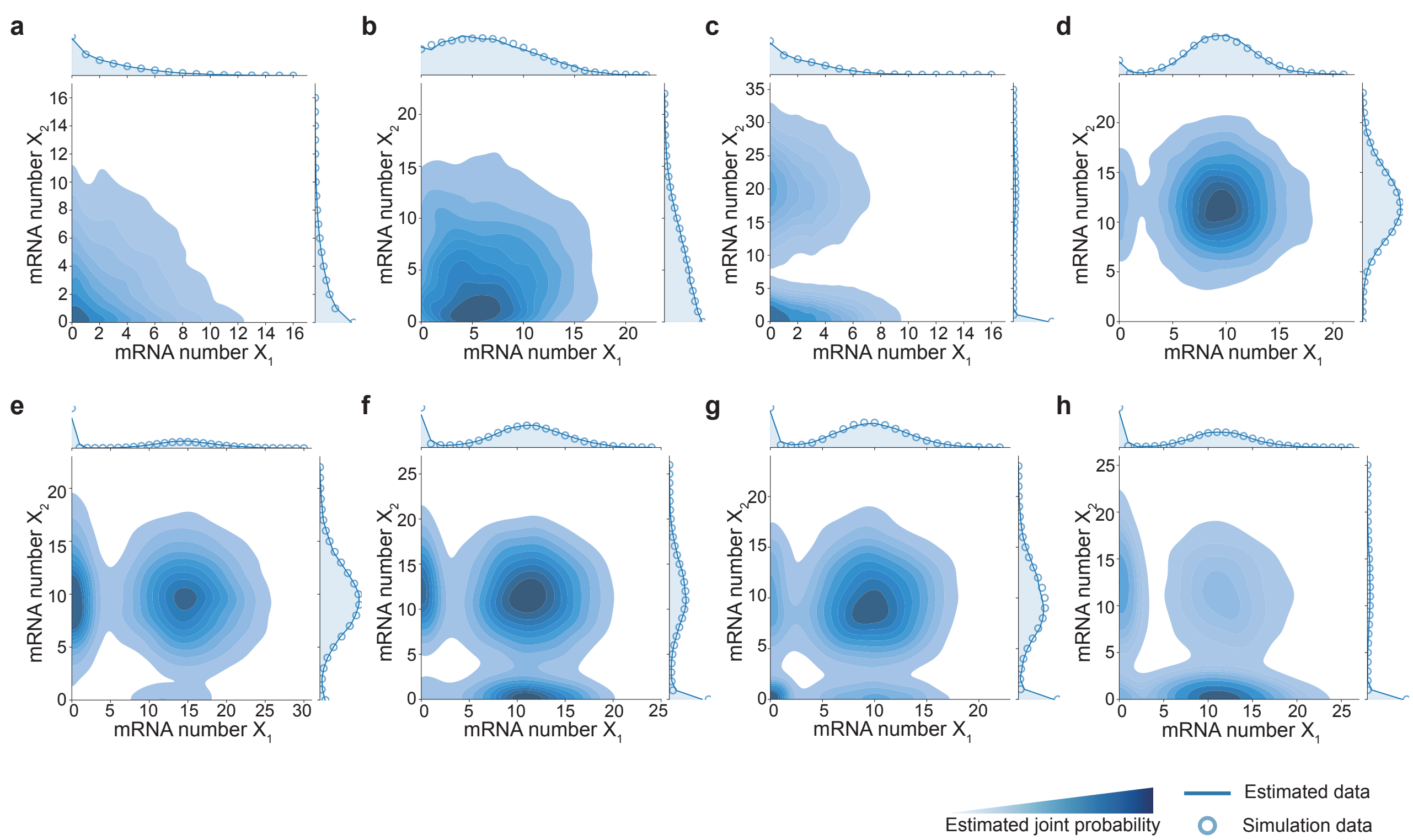

Appendix Figure S2. Mapping results between statistical mechanistic model and dynamic mechanistic model. (A-H) Comparisons of the distributions of simulated data (blue points) and estimated theoretical results (the contour corresponds to the estimated joint probability distribution, and the blue line corresponds to the estimated marginal probability). Parameter values are set in Table S1, and (A-G) correspond to the mapping results of landscapes (Fig. 2B).

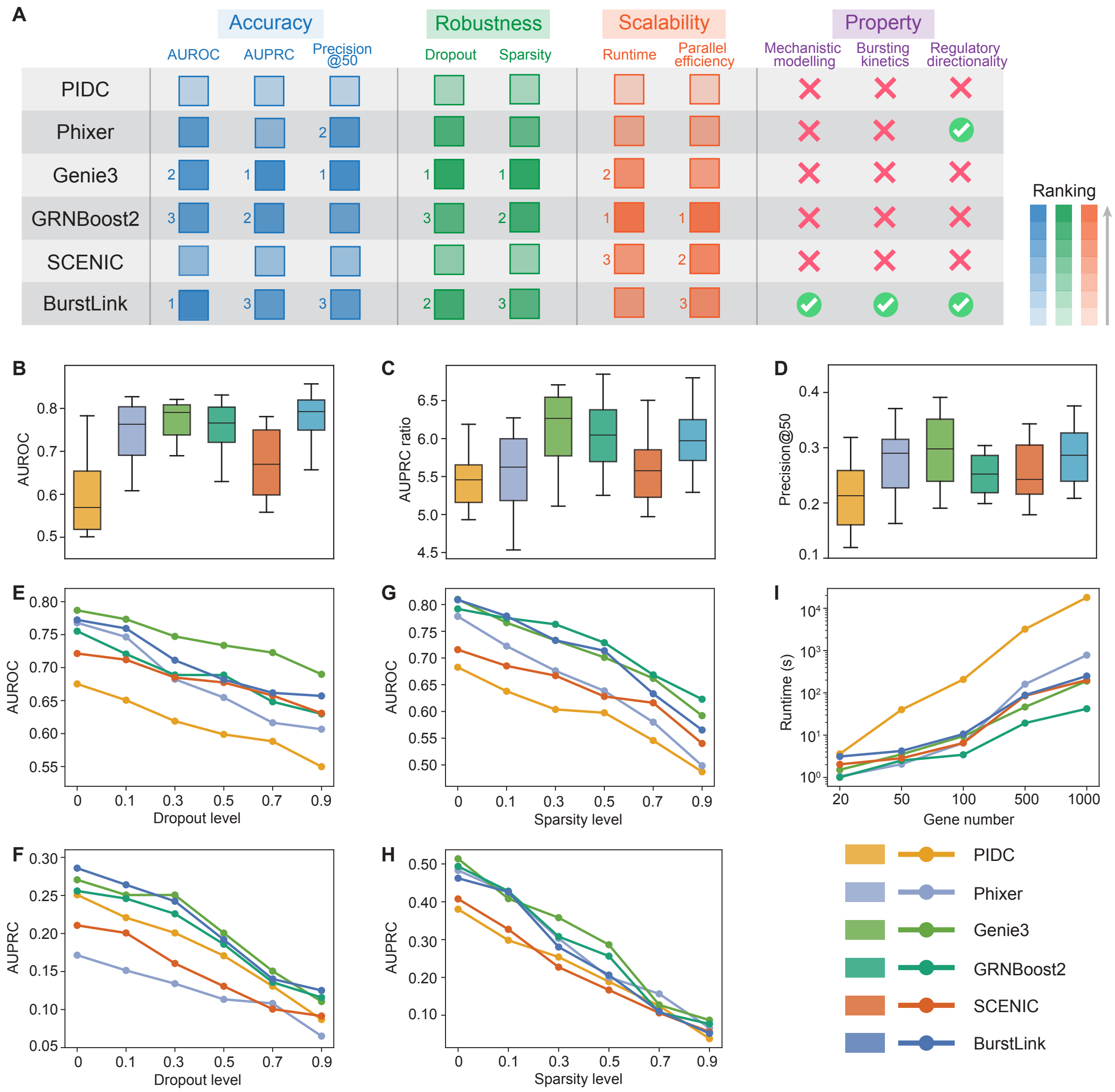

Appendix Figure S3. Benchmarking of BurstLink and representative GRN inference methods. (A) Summary of method rankings across accuracy, robustness, scalability, and methodological properties. (B–D) Comparison of inference accuracy using AUROC, AUPRC normalized by baseline, and Precision@50. (E–H) Performance under increasing dropout and sparsity levels measured by AUROC and AUPRC. (I) Runtime comparison across different gene numbers.

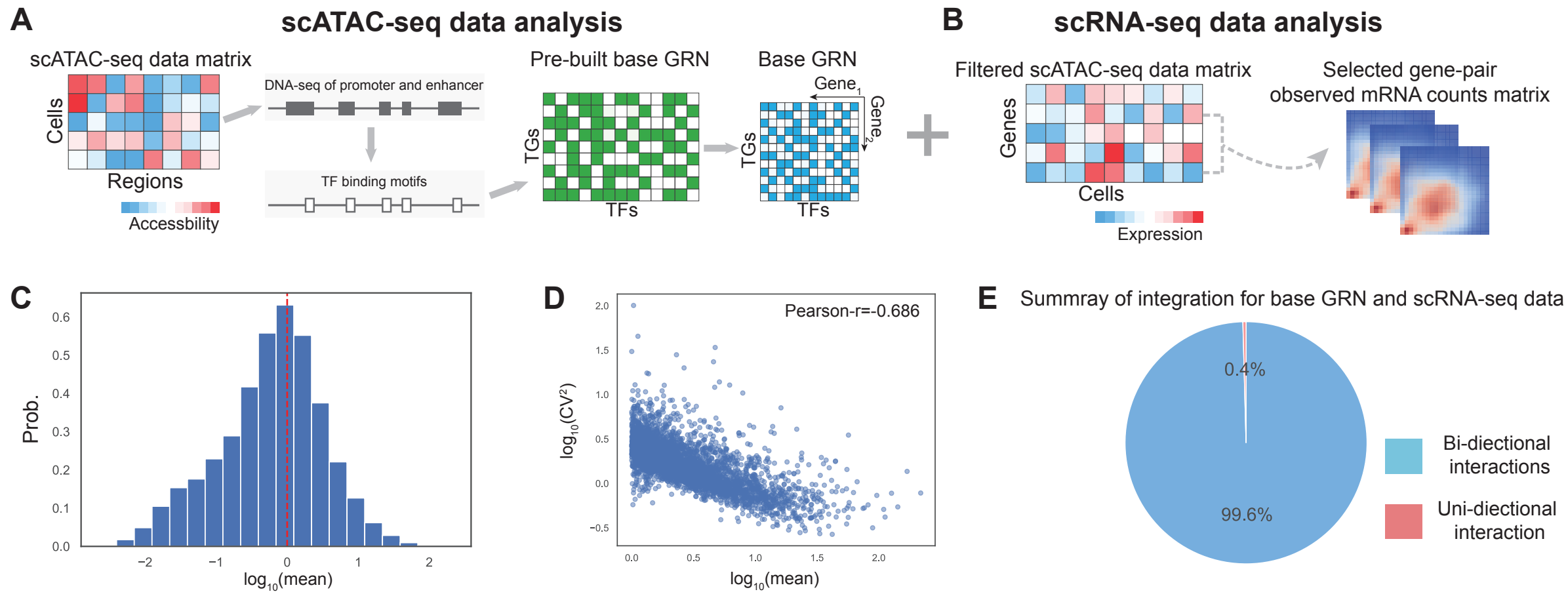

Appendix Figure S4. Data preprocess summary. (A) Construction procedure for base-GRN using scATAC-seq data. Base-GRN assembly can be divided into four steps: (1) identification of promoter and enhancer regions using scATAC-seq data; (2) motif scanning of promoter and enhancer DNA sequences; (3) genomic DNA sequence and TF-binding-motif information provide all potential regulatory links to construct a pre-built base-GRN (Kamimoto et al, 2023); (4) Selection for gene-pair regulation information to construct base-GRN from the pre-built base-GRN. (B) Selection procedure for scRNA-seq data integrated with base-GRN. (C) Histogram of gene-expression mean for all inferred genes. (D) Scatter plot of mean gene-expression levels against CV^2 for all inferred genes. (E) Pie chart for the proportion of regulation forms (bi-, uni-interaction) for all inferred genes integrated with base-GRN and scRNA-seq data.

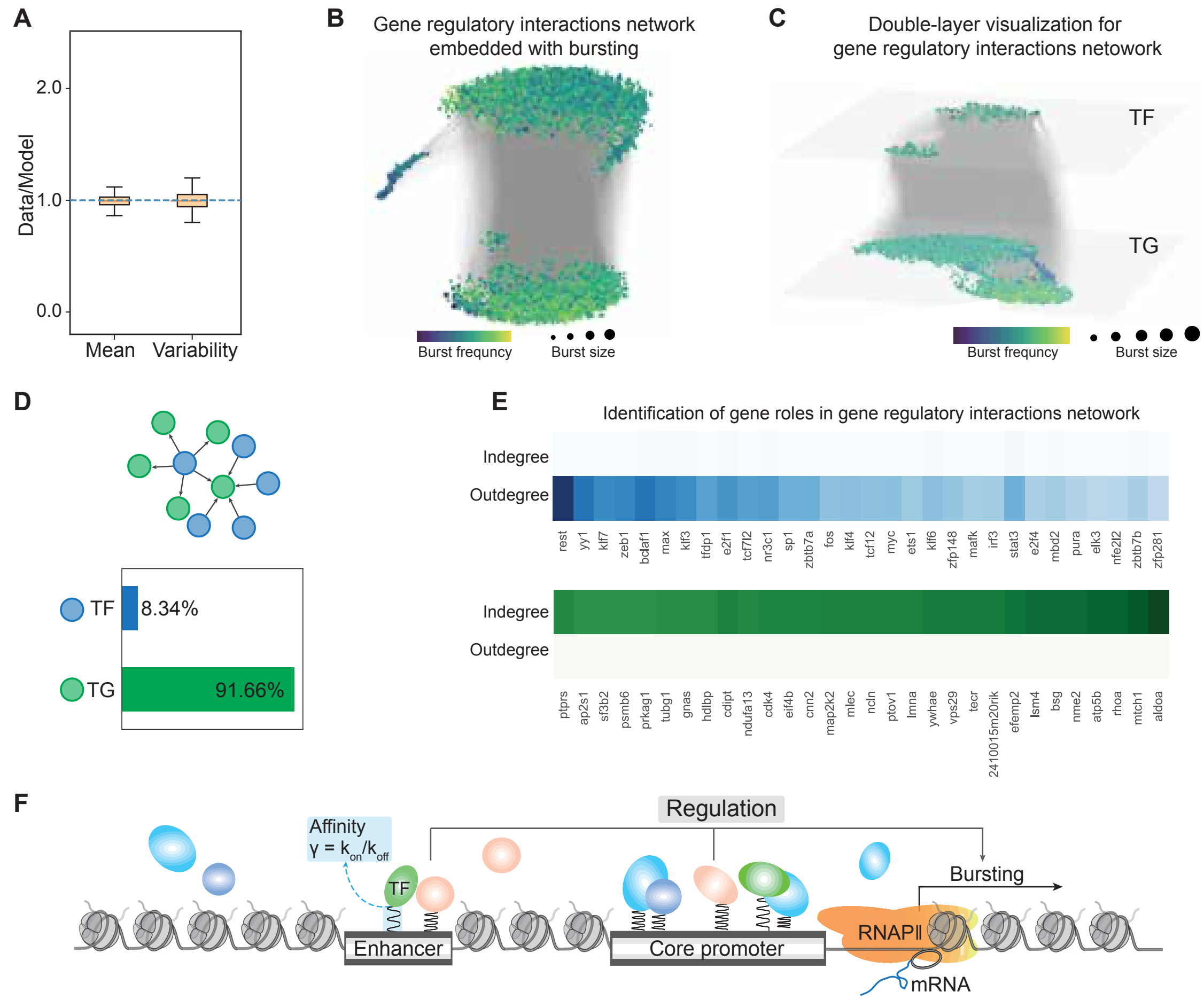

Appendix Figure S5. Visualization of inference results on single-cell multi-omics data of mouse primary fibroblast. (A) Comparison of the mean expression level and the variability between the scRNA-seq dataset and samples generated from the inferred parameters, where the blue dashed line represents those results of the model and the data are completely consistent. (B) UMAP embedding of the inferred gene regulatory interactions network with burst kinetics. (C) Double-layer visualization separating upstream TFs and downstream TGs, with genes positioned by UMAP embedding and encoded by BF and BS. (D) Bar plot for proportion of TFs and TGs. (E) The heatmap for interaction level of the genes as TF and TG in gene regulatory interaction network. (F) Schematic where TFs bind to the specific binding sites of enhancers and promoters on DNA, thereby impacting the transcriptional bursting activity.

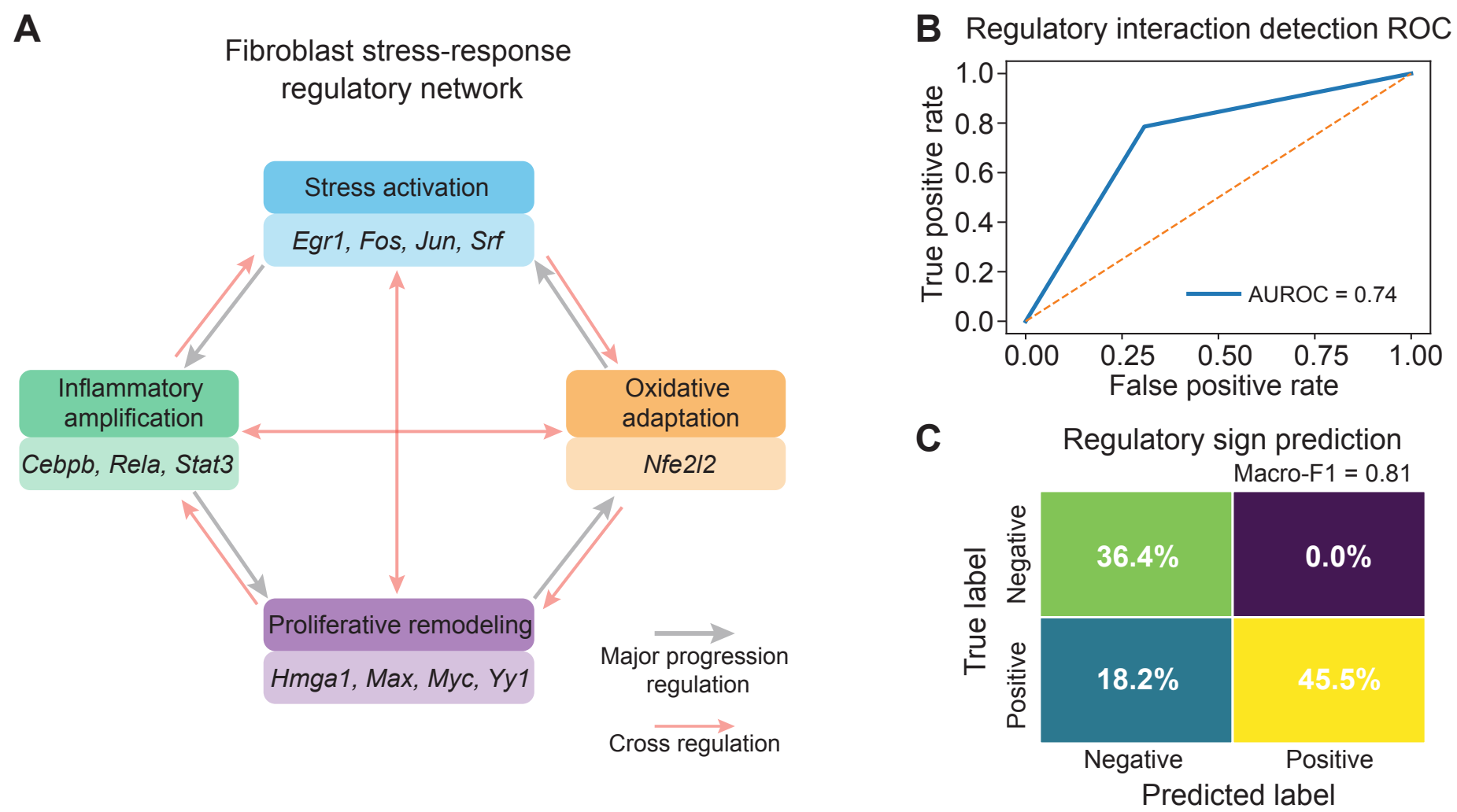

Appendix Figure S6. Literature-supported validation of a fibroblast stress-response regulatory network inferred by BurstLink. (A) Conceptual schematic of a fibroblast stress-response regulatory network constructed from 12 genes identified from mouse embryonic fibroblast (MEF) scRNA-seq data. These regulators were grouped into four interconnected transcriptional programs: stress activation (Egr1, Fos, Jun, and Srf), inflammatory amplification (Cebpb, Rela, and Stat3), proliferative remodeling (Hmga1, Max, Myc, and Yy1), and oxidative adaptation (Nfe2l2). Gray arrows indicate major progression relationships among functional states, whereas red arrows denote cross-regulatory interactions between modules. (B) Literature-based validation of regulatory interaction detection. Receiver operating characteristic (ROC) analysis demonstrates the ability of BurstLink to recover literature-supported regulatory interactions, achieving an AUROC of 0.74. (C) Validation of inferred regulatory signs using literature-supported activating and repressive interactions. The normalized confusion matrix shows the agreement between curated regulatory signs and BurstLink predictions, with a macro-F1 score of 0.81.

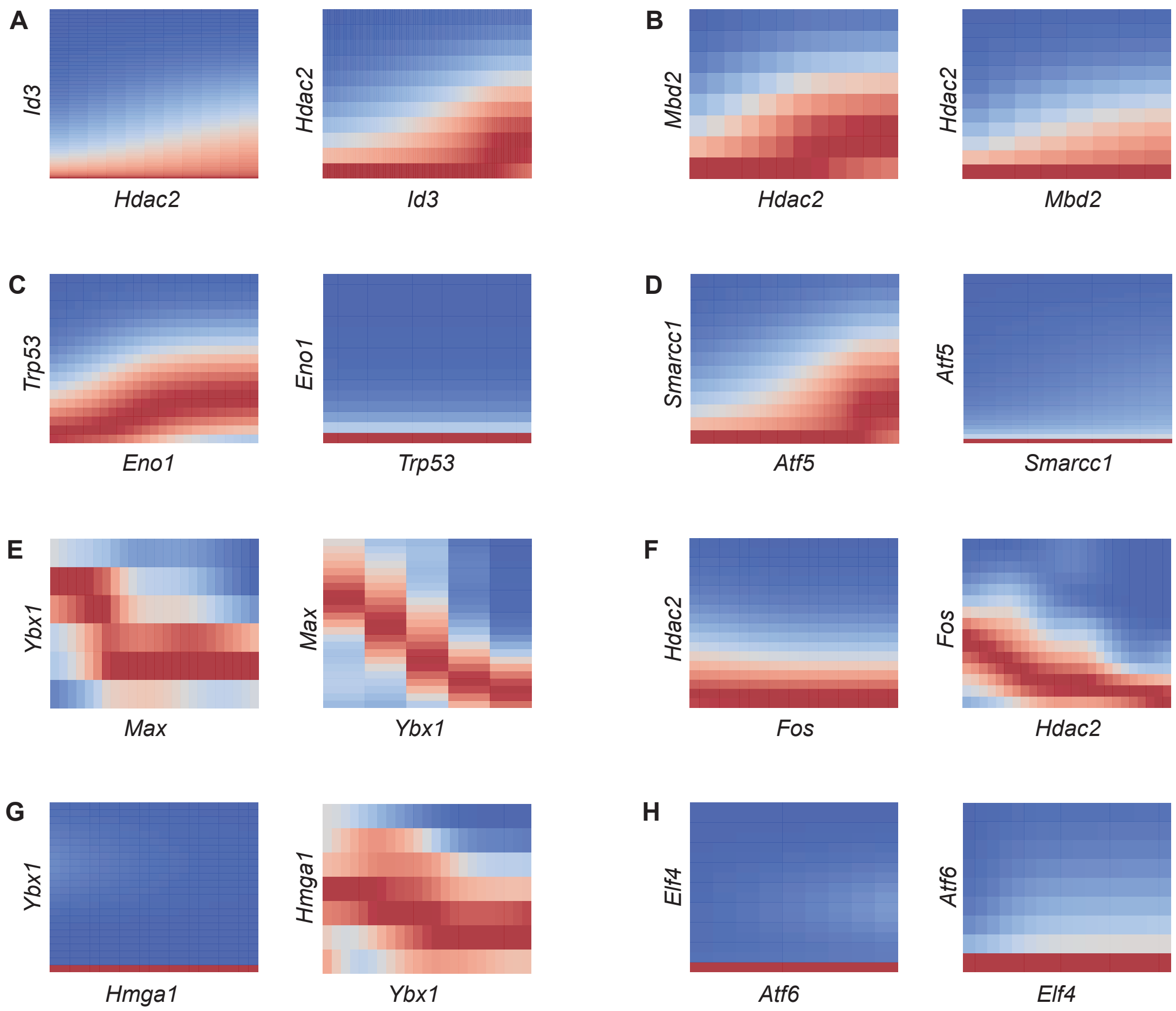

Appendix Figure S7. Examples of the inferred gene-pair regulation visualization from primary mouse fibroblast. (A-H) The inferred gene-pair regulatory interaction visualization for different regulation forms (bi-, uni-, non-regulation). These regulation loop types our method predicted have been confirmed by biological experiments (Table S4).

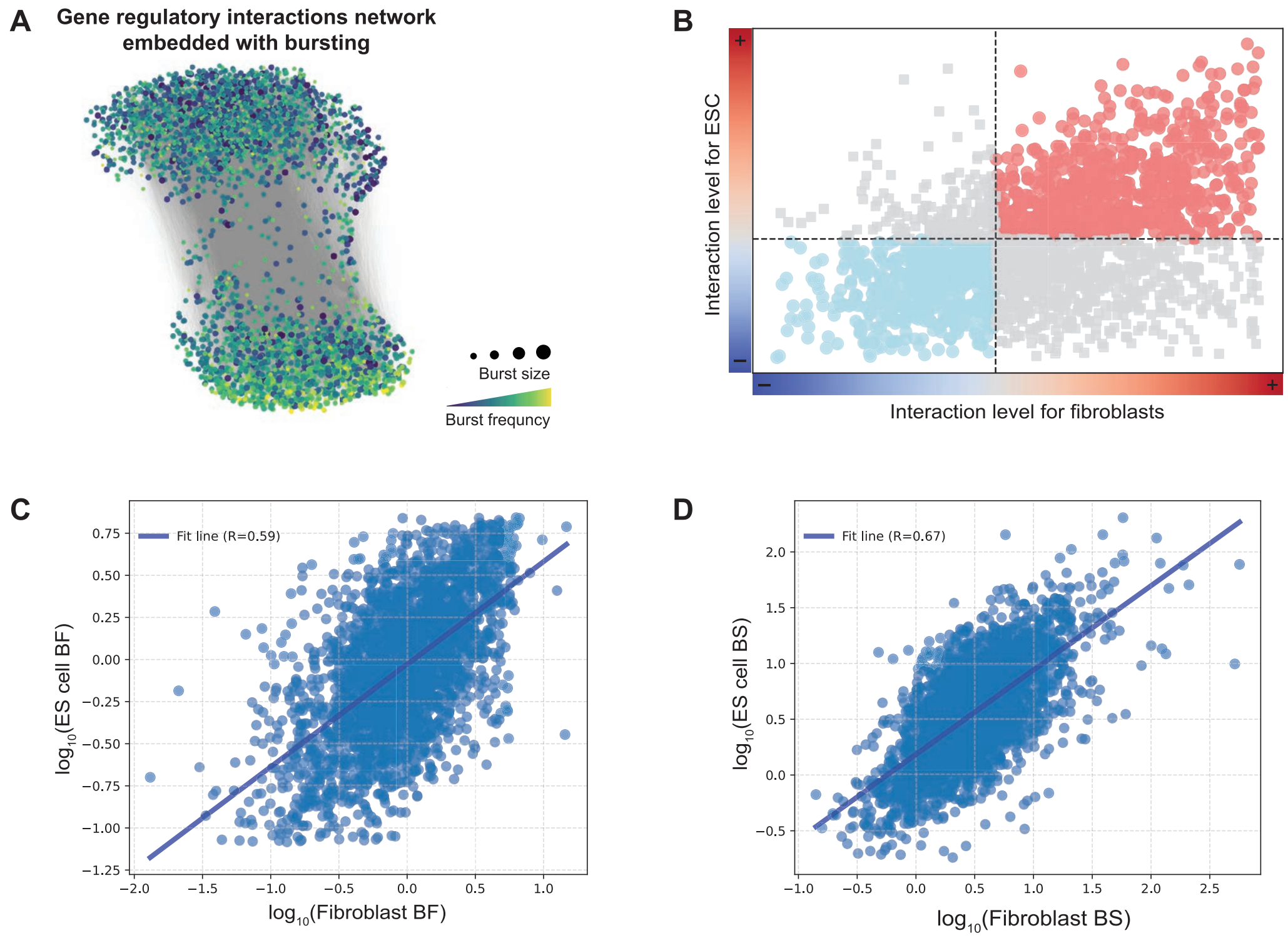

Appendix Figure S8. Genome-wide inference on single-cell multi-omics data of mouse embryonic stem (ES) cells. (A) UMAP embedding of the inferred gene regulatory interactions network with bursting, where the size and color of points represent burst size and frequency, respectively. (B) Scatter plots of overall interaction levels in mouse fibroblasts and ES cells, where circles represent the same regulation types (light red color denotes positive regulation, light blue color denotes negative regulation), and gray squares represent different regulation types in these two cell types. (C, D) Scatter plots of BF and BS in mouse fibroblasts and ES cells.

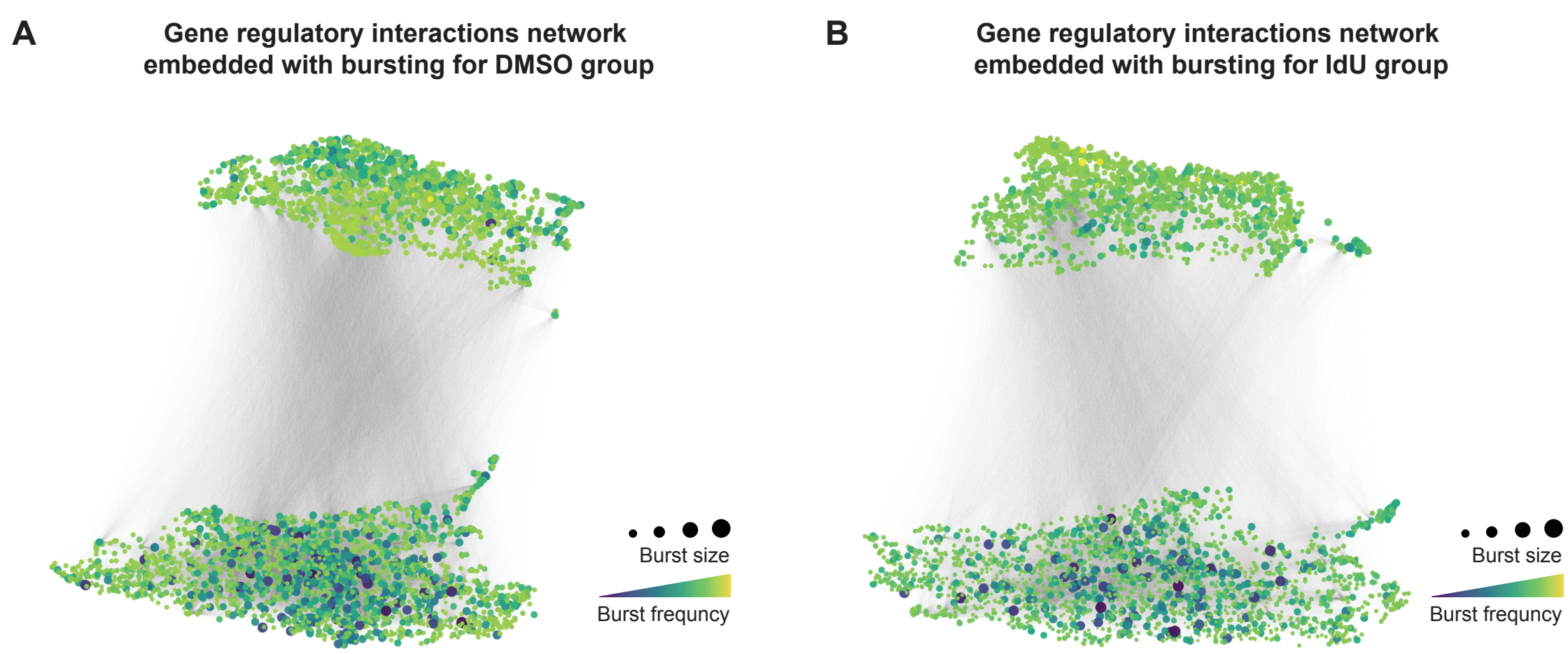

Appendix Figure S9. Visualization for the inference results on DMSO and IdU dataset. UMAP embedding of the inferred gene regulatory interactions network with bursting for DMSO and IdU groups respectively, where the size and color of points represent burst size and frequency.

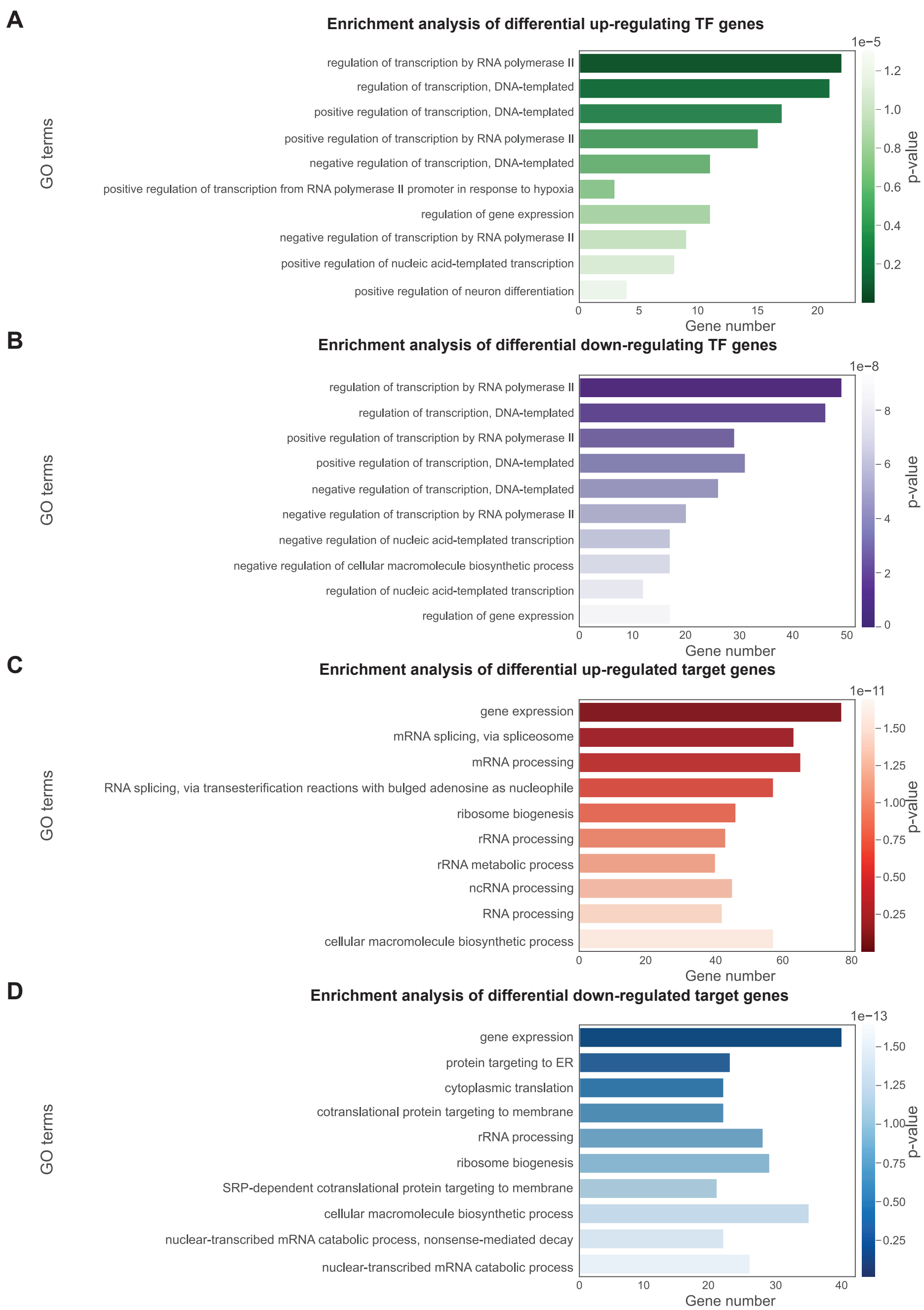

Appendix Figure S10. Gene GO enrichment analyses are performed on the differential genes set (up-regulating TFs, down-regulating TFs, up-regulated TGs and down-regulated TGs) to obtain enrichment pathway diagrams, respectively.

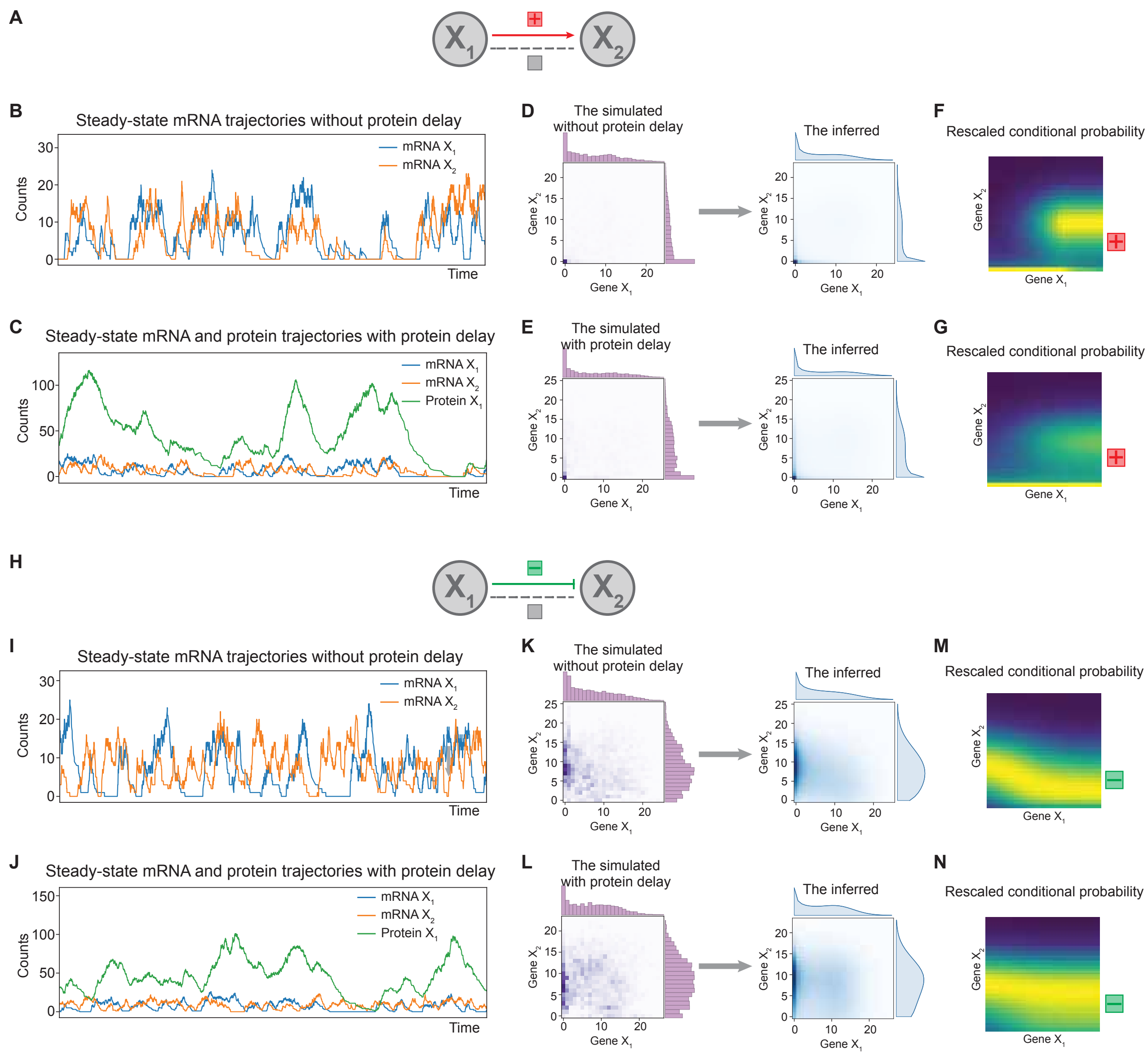

Appendix Figure S11. BurstLink recovers regulation under explicit protein dynamics with time delay. The toggle-switch model was extended so that regulation is mediated by the TF's protein, with protein degrading 4× slower than mRNA. (A, H) Ground-truth schematics: X_1 positively (A) or negatively (H) regulates X_2; reverse direction non-regulation. (B, I) Steady-state mRNA trajectories without protein delay; (C, J) mRNA and protein trajectories with protein delay, showing slower, buffered protein dynamics. (D, E, K, L) Simulated (left) and BurstLink-inferred (right) joint count distributions, without (D, K) and with (E, L) protein delay. (F, G, M, N) Rescaled conditional-probability heatmaps, without (F, M) and with (G, N) protein delay.

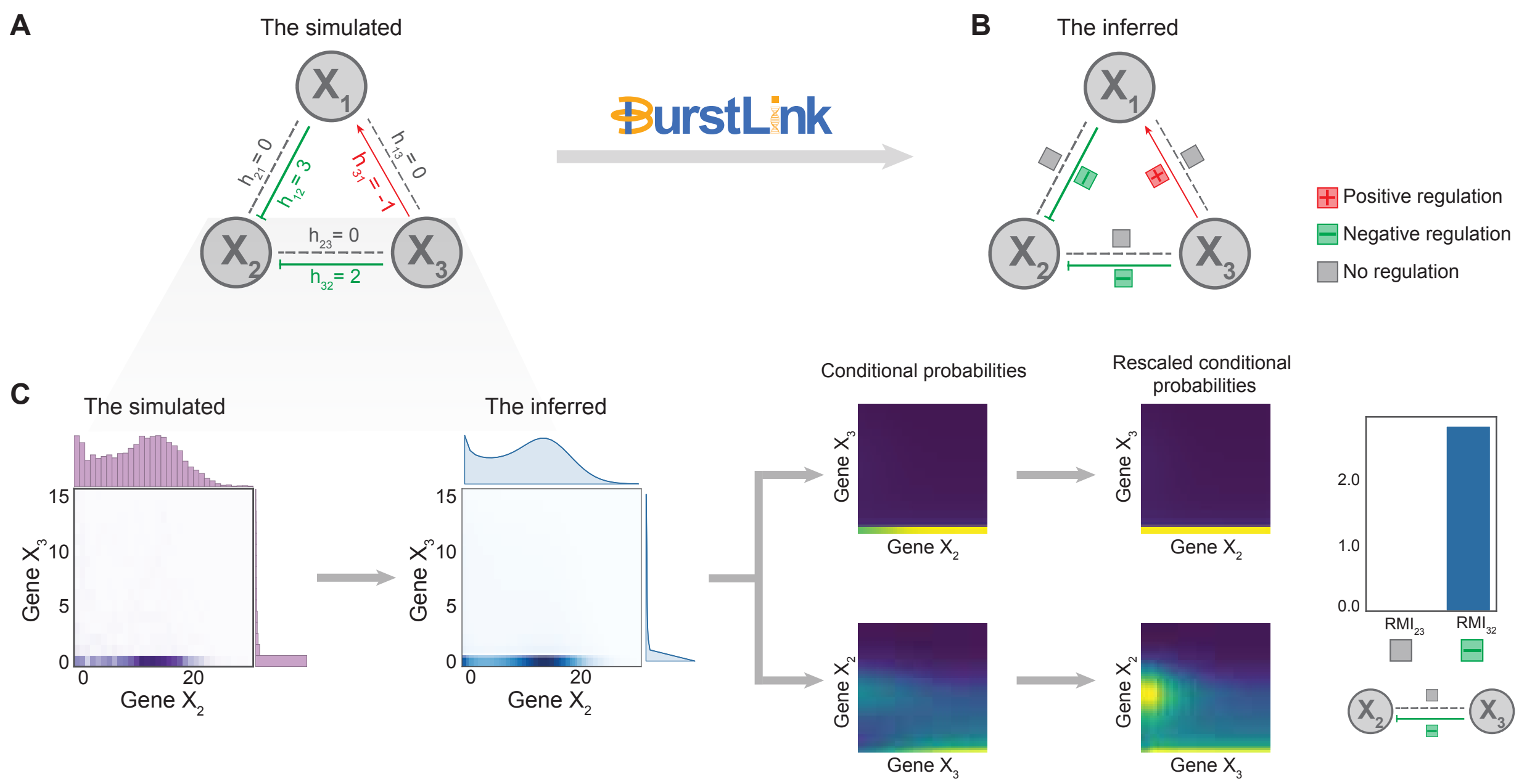

Appendix Figure S12. Analytical illustration of directional regulation inference from snapshot data. (A) Simulated three-gene regulatory network used as an example. Red arrows denote positive regulation, green arrows denote negative regulation, and gray lines indicate the absence of direct regulation. (B) Regulatory network inferred by BurstLink from the simulated snapshot gene expression data. The inferred regulatory interactions accurately recover the underlying network structure, including both regulatory directionality, regulation types and regulation strengths. (C) Illustration of the inference procedure for a representative gene pair. Starting from simulated snapshot single-cell expression data, BurstLink first reconstructs the underlying steady-state gene expression distribution. Conditional probability distributions and rescaled conditional probability distributions are then derived from the inferred distribution, from which regulation response curves and regulatory interaction statistics are calculated. These statistics are subsequently used to infer the regulatory type, directionality, and strength of the interaction between the two genes.

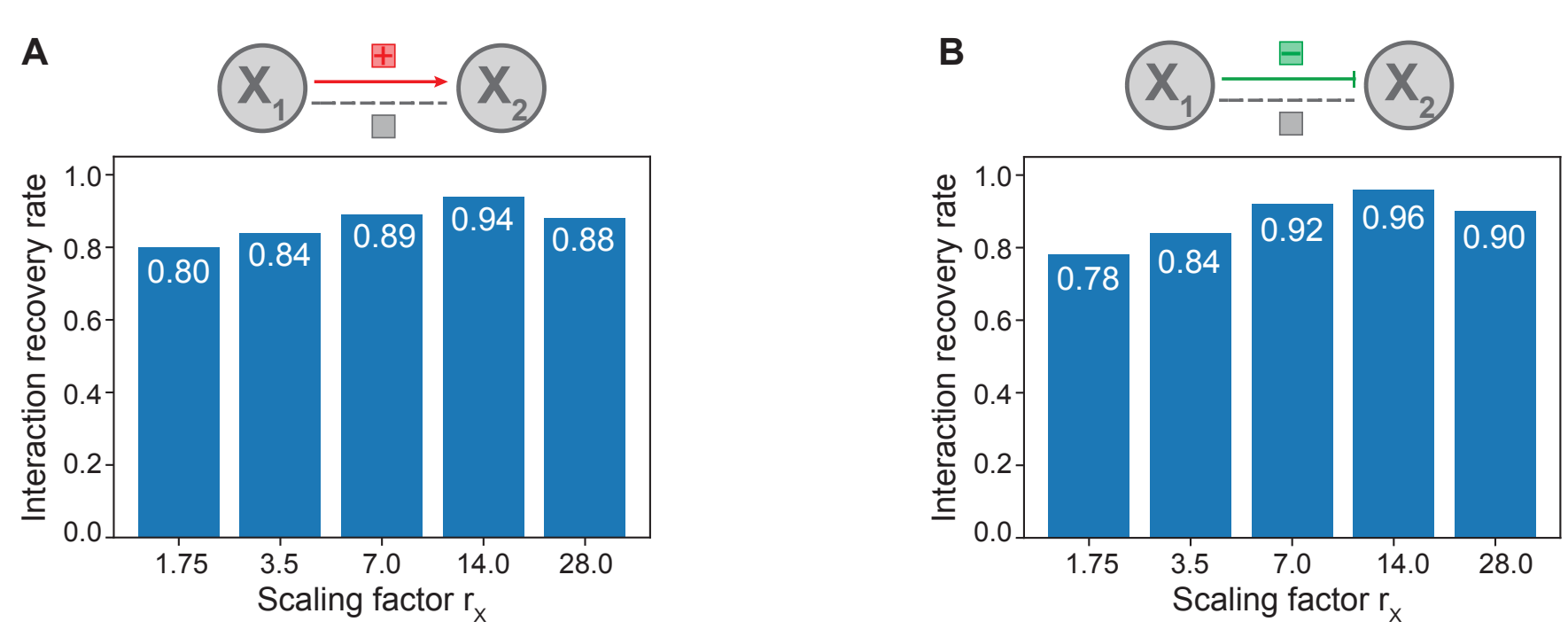

Appendix Figure S13. Sensitivity of BurstLink inference to r_X. Synthetic data were generated from the toggle-switch model (X_1 regulates X_2; reverse direction non-regulation) with r_X varied over a dynamic range, and BurstLink was re-run at each setting. The interaction recovery rate is the fraction of cases where

both the direction and type of regulation were correctly identified. (A) Positive and (B) negative regulation. Recovery stays high across the full range (0.78–0.96).

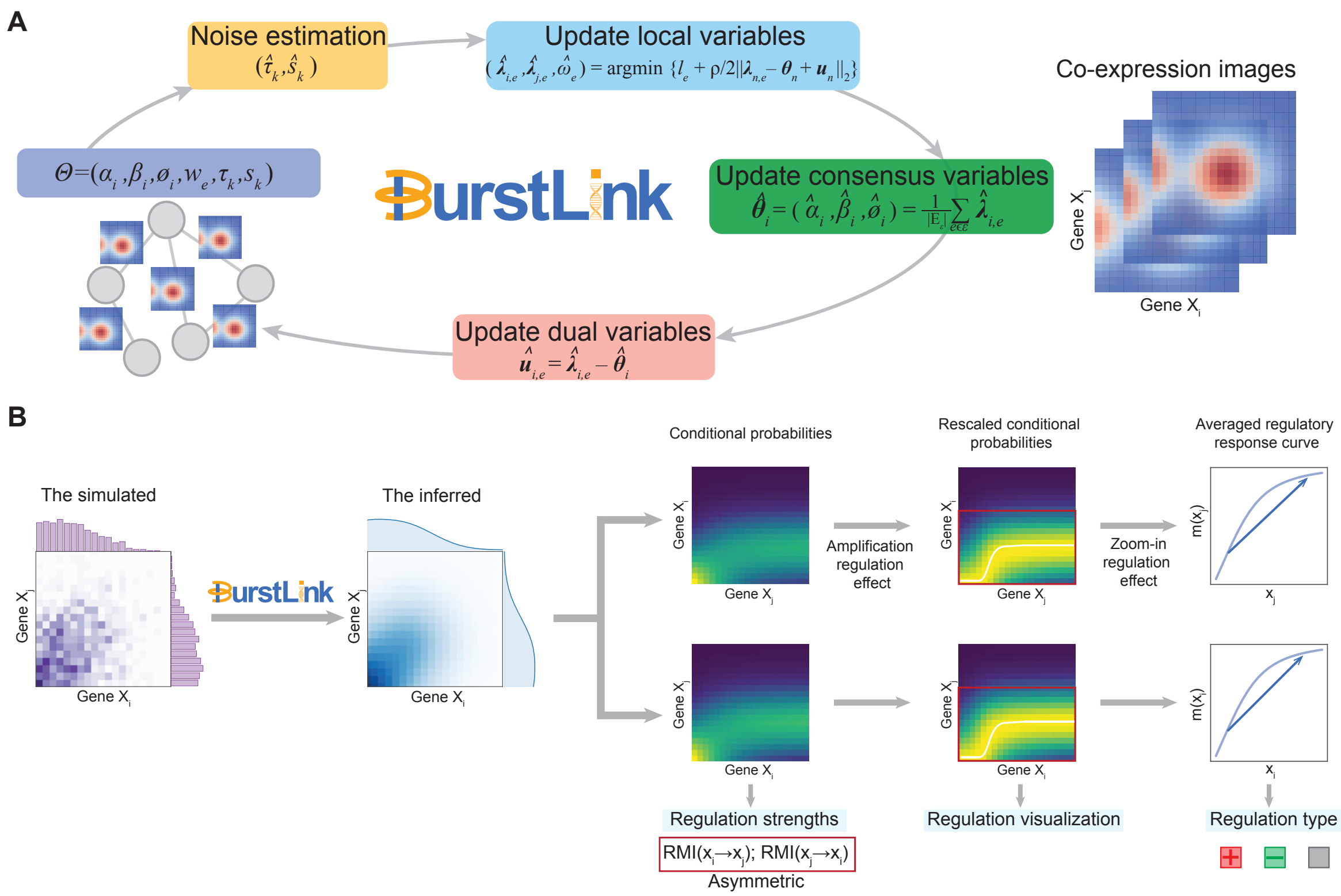

Appendix Figure S14. Schematic illustration of the BurstLink inference workflow. (A) ADMM-based optimization framework for parameter inference. Technical noise and cell-size–related parameters are estimated firstly. The algorithm then iteratively updates gene-pair–specific local variables, node-level consensus variables, and dual variables until convergence. This procedure yields consistent estimates of gene-expression related and bursting parameters across all gene pairs. (B) Algorithmic inference of gene–gene interactions from inferred probability distributions. Using the inferred joint and marginal distributions, BurstLink computes conditional probabilities for each gene pair. These conditional probabilities are rescaled to enhance regulatory signals, followed by column-wise aggregation to obtain averaged regulatory response curves. Regulation strength is quantified via reweighted mutual information, while regulation type (positive, negative, or none) is determined based on the Spearman correlation of the aggregated response curves, enabling quantitative and interpretable characterization of gene–gene regulatory interactions.

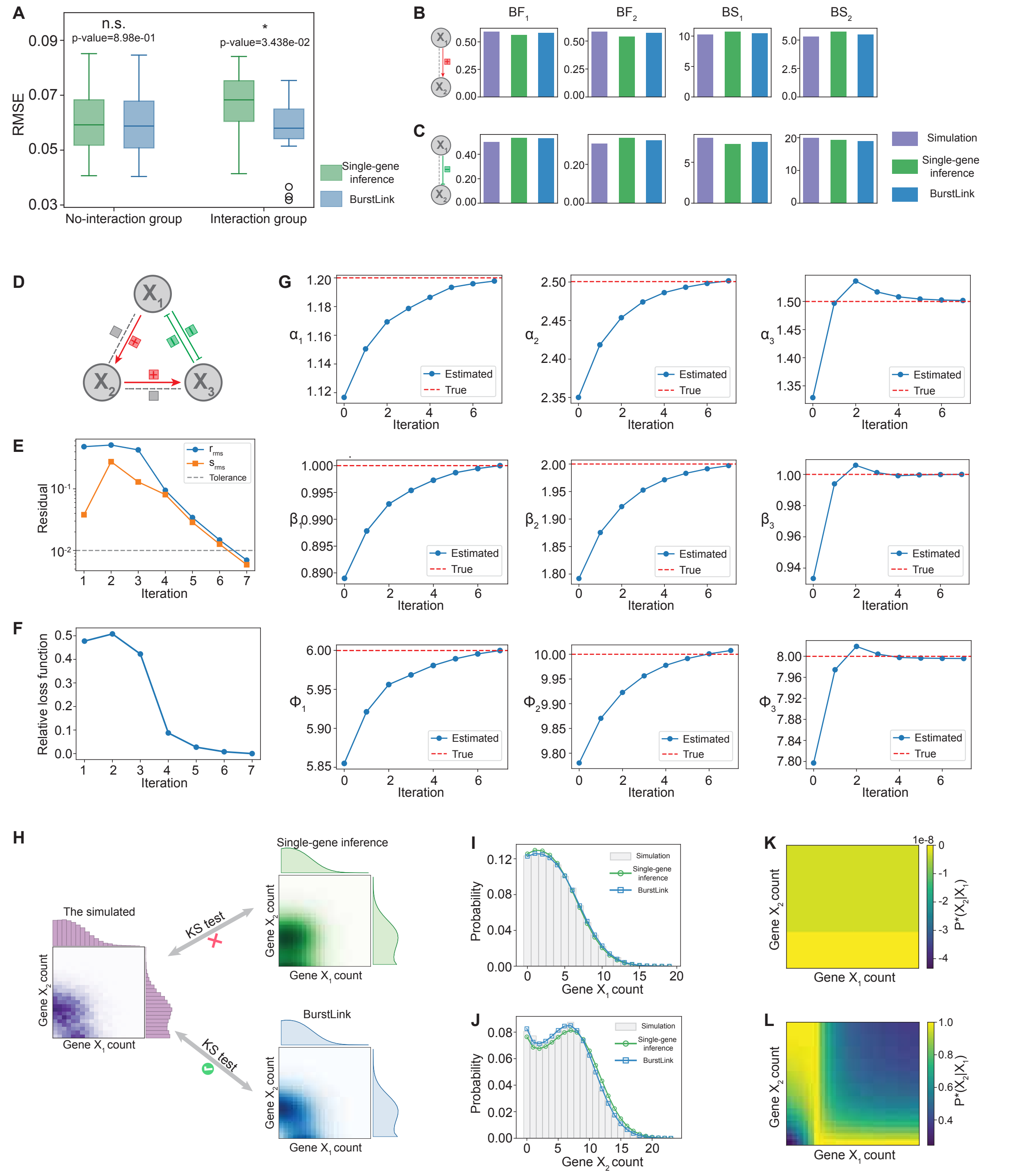

Appendix Figure S15. BurstLink refines burst kinetic estimates beyond single-gene fitting. (A) Comparison of burst kinetic parameter estimation errors between single-gene inference and BurstLink using simulated datasets with known ground-truth parameters. Gene pairs were divided into non-interacting and interacting groups. RMSE was calculated between inferred and true burst frequency (BF) and burst size (BS). (B–C) Two representative examples with positive and negative regulatory interactions, respectively. The inferred burst frequency (BF) and burst size (BS) obtained by single-gene inference and BurstLink are compared with the corresponding ground-truth values. (D) Simulated three-gene regulatory

network used to evaluate parameter refinement during ADMM optimization. (E) Evolution of ADMM primal residual and dual residual during optimization. (F) Evolution of the objective loss function during ADMM optimization. (G) Iterative trajectories of parameters. (H) Comparison of reconstructed joint gene expression distributions for a two-gene regulatory system. The simulated ground-truth distribution is shown on the left. Single-gene inference reconstructs marginal distributions but fails to recover the joint dependency structure and does not pass the Kolmogorov–Smirnov (KS) test. In contrast, BurstLink recovers the joint distribution and passes the KS test. (I–J) Marginal expression distributions of the two genes from single-gene inference and BurstLink, respectively. (K–L) Conditional probability landscapes inferred from single-gene inference and BurstLink, respectively.

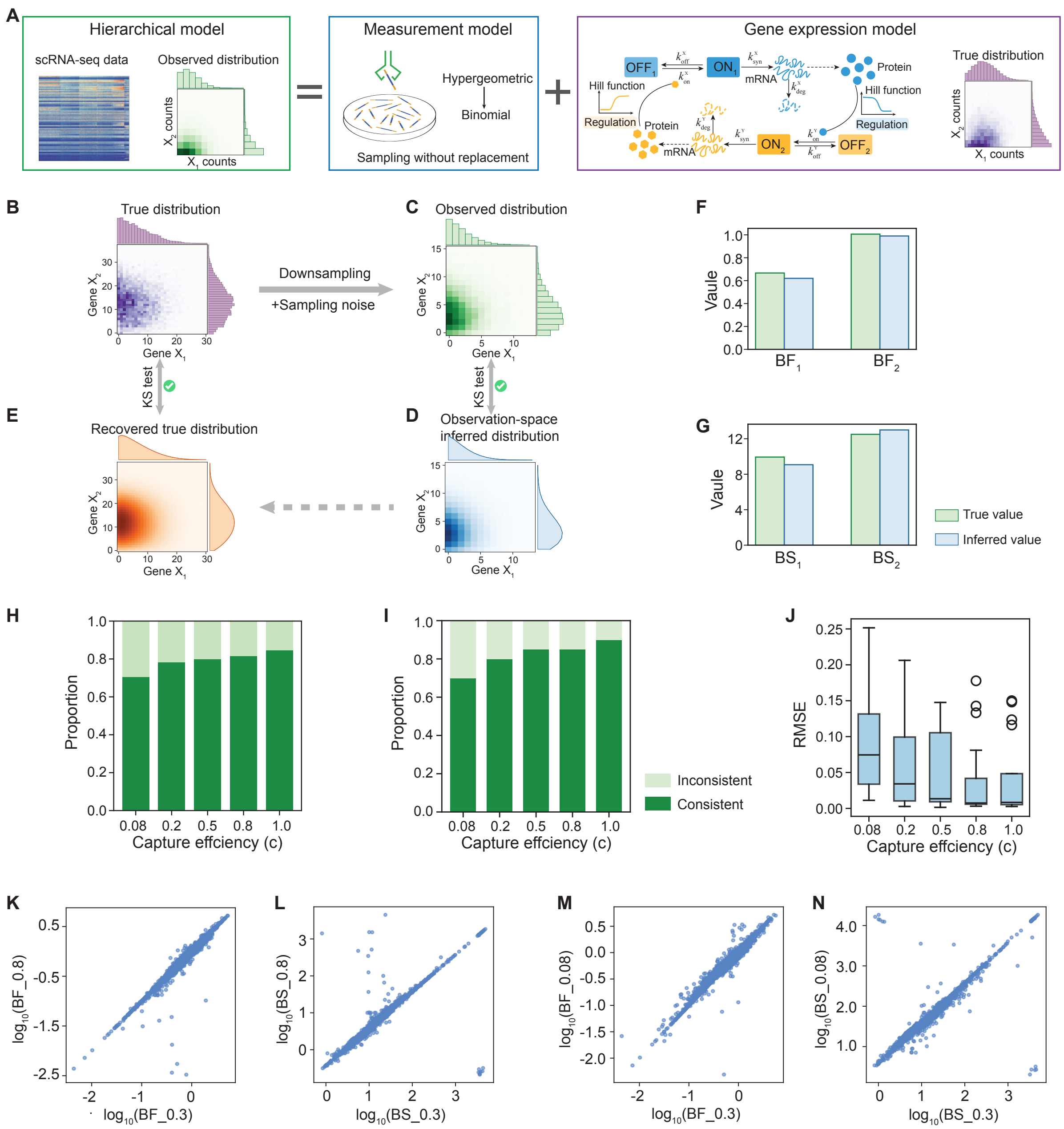

Appendix Figure S16. Benchmarking of BurstLink robustness under capture efficiency variation. (A) Hierarchical model linking underlying gene expression model and measurement model. (B-G) Recovery of gene expression distributions and burst kinetic parameters from downsampled observations. (F-G) Accuracy of burst parameter inference across different capture efficiencies, quantified by the RMSE between inferred and true kinetic parameters. (H–J) Distributional consistency between recovered and true expression distributions across different capture efficiencies. (K–N) Sensitivity analysis using real scRNA-seq data.

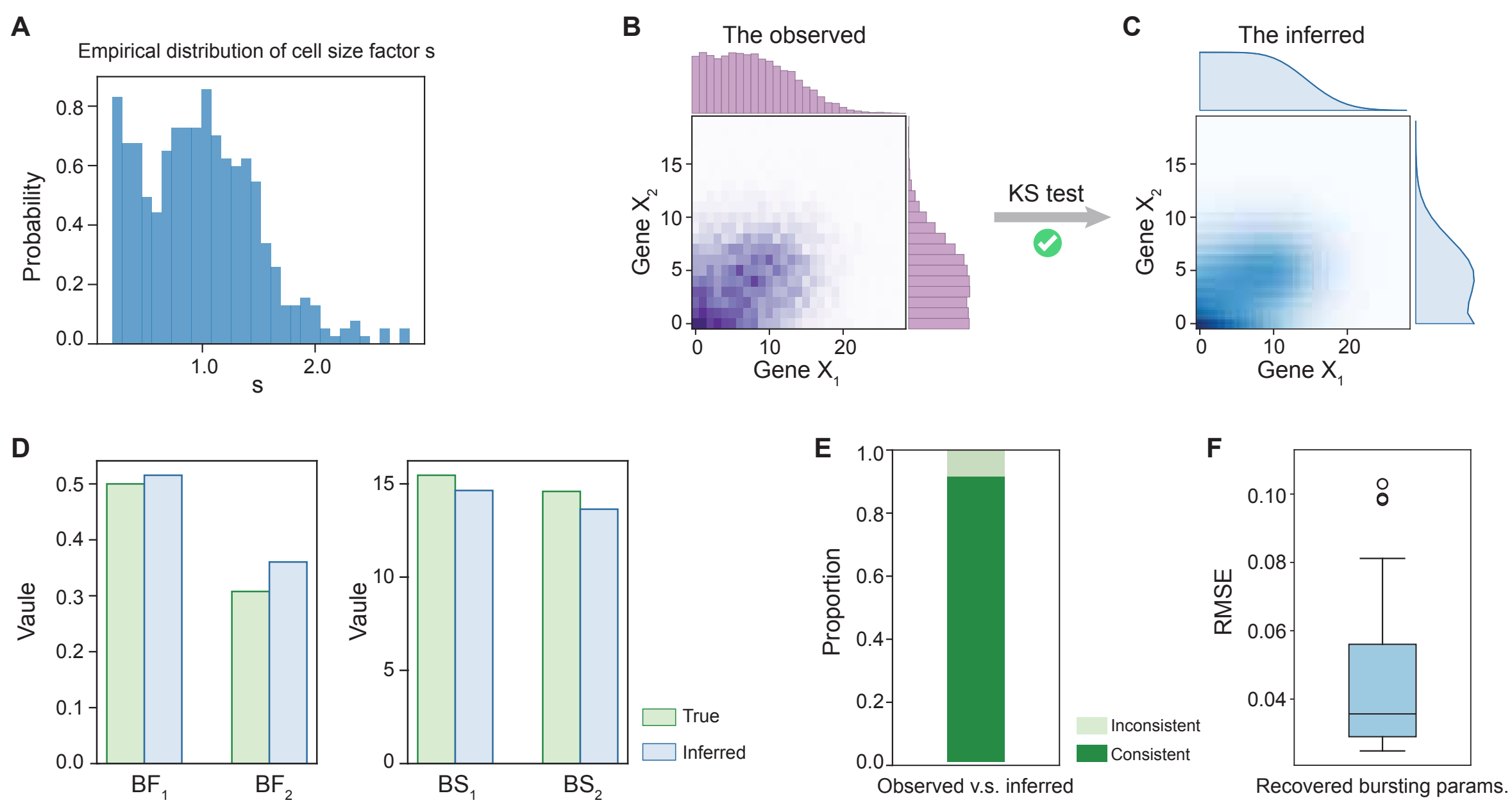

Appendix Figure S17. Benchmarking of BurstLink robustness under cell-size-associated extrinsic variability. (A) Empirical distribution of cell-size factors estimated from a real scRNA-seq dataset based on per-cell library size. (B) Observed joint gene-expression distribution generated by introducing cell-size-associated scaling. (C) Joint gene-expression distribution inferred by BurstLink from the observed data shown in (B). (D) Comparison of burst frequency (BF) and burst size (BS) between the ground-truth parameters and the parameters inferred by BurstLink for the representative example shown in (B–C). (E) Proportion of simulations in which the inferred distributions were statistically consistent with the corresponding observed distributions across 100 independent simulations, as assessed by the Kolmogorov–Smirnov test. (F) Distribution of the root mean square error (RMSE) between inferred and ground-truth bursting kinetic parameters across 100 independent simulations.

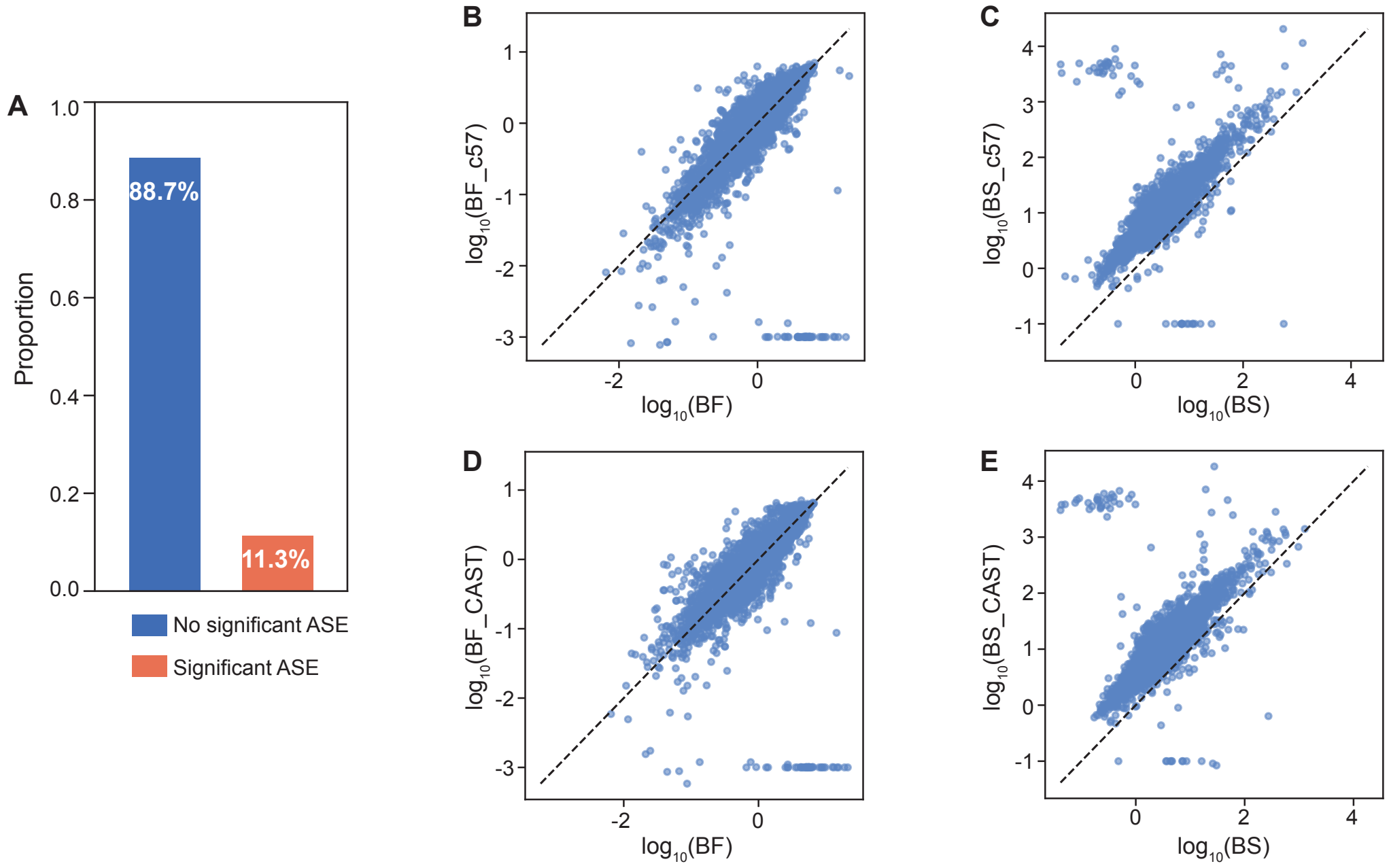

Appendix Figure S18. Evaluation of allele-specific expression distribution consistency and benchmarking of BurstLink-inferred bursting kinetics with Larsson et al. (A) Distributional consistency between CAST and C57 allele-specific expression (ASE) profiles assessed by the Kolmogorov–Smirnov (KS) test. (B-C) Comparison of BurstLink-inferred burst frequency (BF) and burst size (BS) at the gene level with the published allele-specific estimates reported by Larsson et al. for the CAST allele. (D-E) Comparison of BurstLink-inferred burst frequency (BF) and burst size (BS) at the gene level with the published allele-specific estimates reported by Larsson et al. for the C57 allele.

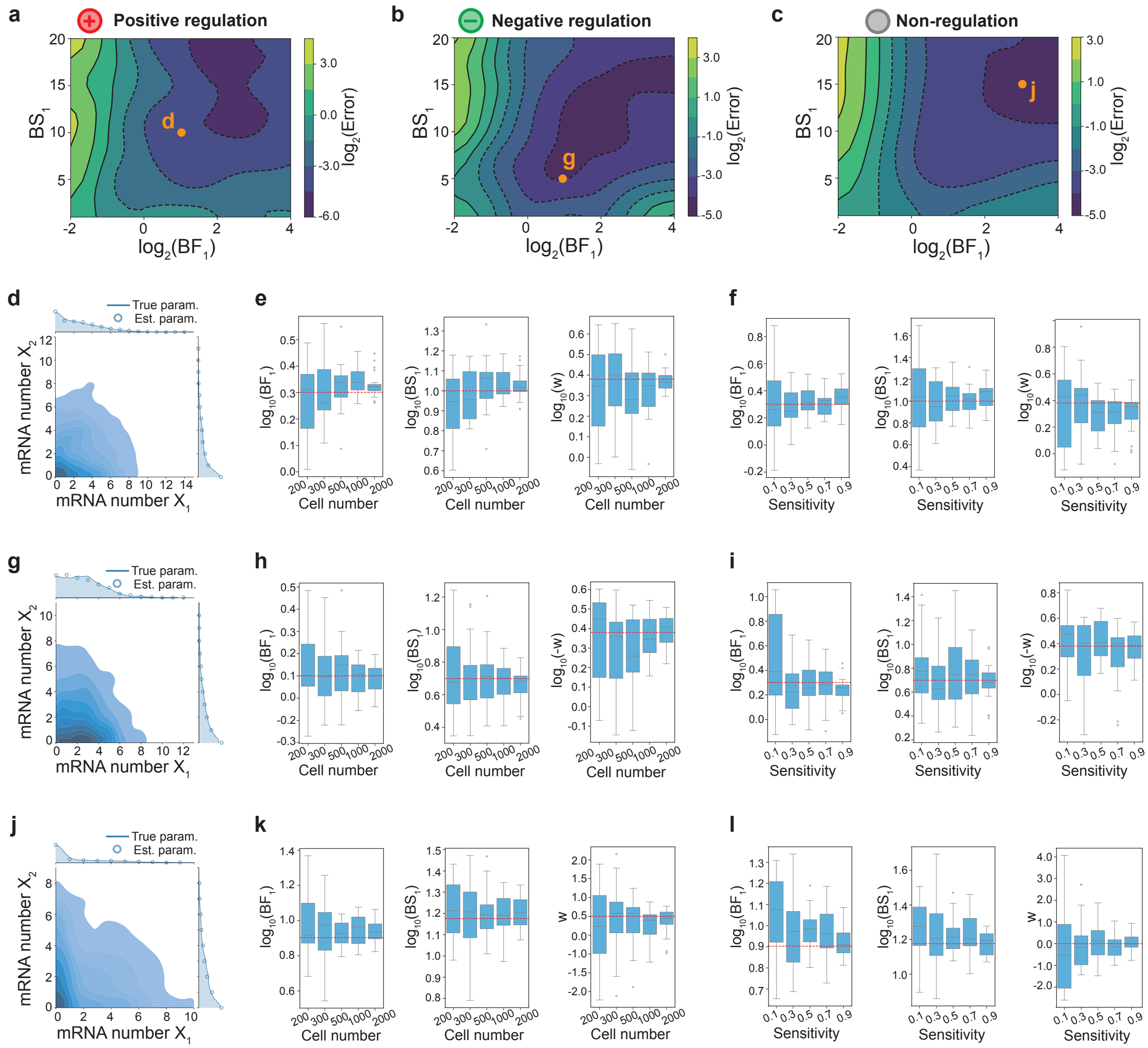

Appendix Figure S19. Precision and robustness of inferred results for the statistical mechanistic model. (A-C) Contours illustrate the error (color bars) of inferred burst kinetics from simulated expression data for positive, negative, and non-regulation cases by Gibbs sampler. The orange dots represent burst frequencies and sizes in the examples shown in (D–L). (D) mRNA joint probability distribution for the fixed $\mathrm{BS}_1 = 10, \mathrm{BS}_2 = 10, \mathrm{BF}_1 = 2, \mathrm{BF}_2 = 2$, where the blue points are for the simulated data, the contour and blue solid line for the estimated joint and marginal probability distribution. (E, F) Boxplots for inferred burst frequencies, burst sizes, and the parameter related to regulation as functions of different cell numbers and random deletions (sensitive), where the red dashed line corresponds to the true parameter. (G–I) Analysis of robustness inference similar to (D) and (E, F) for the fixed $\mathrm{BS}_1 = 5, \mathrm{BS}_2 = 10, \mathrm{BF}_1 = 1.25, \mathrm{BF}_2 = 2$. (J–L) Analysis of robustness inference similar to (J) and (K, L) for the fixed $\mathrm{BS}_1 = 15, \mathrm{BS}_2 = 10, \mathrm{BF}_1 = 8, \mathrm{BF}_2 = 2$.

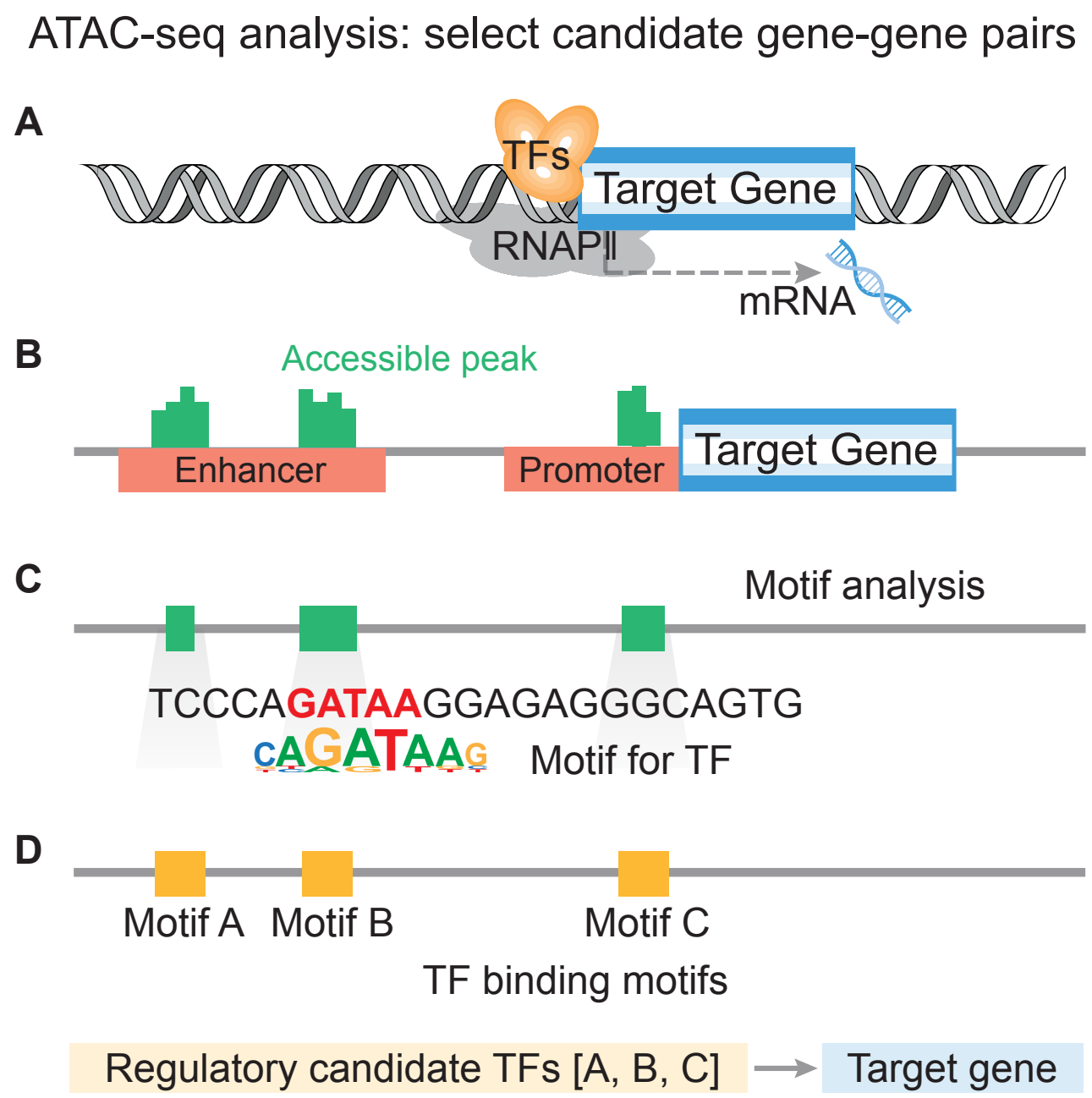

Appendix Figure S20. Selection of candidate TF-TG pairs using scATAC-seq data. (A) Schematic of TF-mediated regulation of a target gene (TG). (B) Identification of accessible chromatin peaks in putative regulatory regions of the TG, including promoters and enhancers. (C) Extraction of DNA sequences from accessible peaks and analysis of TF binding motifs. (D) Selection of candidate TFs whose motifs are detected in accessible regulatory regions of the TG.

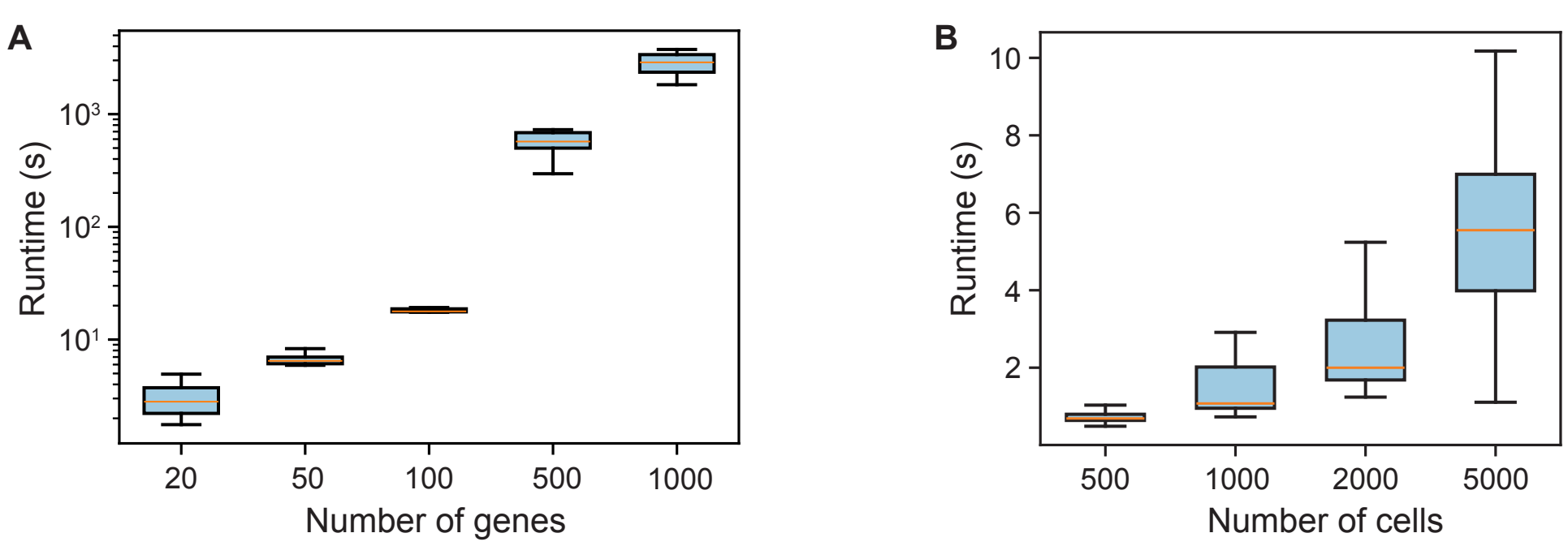

Appendix Figure S21. Computational scalability of BurstLink. (A) Boxplots of runtime distributions for subnetworks containing different numbers of genes. (B) Boxplots of runtime distributions for datasets containing different numbers of cells.

S3 Appendix Tables

Appendix Table S1. Parameter settings for the dynamic mechanistic model used to generate the landscapes in Fig. S2.

	k_{on}^{X}	k_{on}^{Y}	k_{off}^{X}	k_{off}^{Y}	k_{syn}^{X}	k_{syn}^{Y}	k_{deg}^{X}	k_{deg}^{X}	$\frac{r_X}{K_X}$	$\frac{r_Y}{K_Y}$	h_X	h_Y	ε_X	ε_Y	H_0
a	1.0	1.0	2.0	2.0	10	10	1	1	1	1	0	0	0.1	0.1	False
b	2.0	2.0	1.0	1.0	12	12	1	1	0.15	0.15	2	-2	0.01	0.01	False
c	1.0	0.01	1.0	0.01	5	20	1	1	1	1	0	1	0.1	0.1	False
d	0.1	1.0	0.01	0.01	10	12	1	1	1	1	-2	-2	0.1	0.1	False
e	0.1	1.0	0.01	0.01	15	10	1	1	1	1	5	2	0.1	0.1	False
f	0.4	0.4	0.02	0.02	12	12	1	1	1	1	1	1	0.1	0.1	False
g	0.3	0.3	0.02	0.02	12	12	1	1	1	1	1	1	0.1	0.1	False
h	0.2	0.2	0.01	0.01	12	12	1	1	1	1	0	0	0.1	0.1	False

Note: rejection for the null hypothesis H_0 (False) denotes that the distribution test has been passed, i.e., the inferred distribution is regarded as the same with the scRNA-seq data at a 95% confidence interval.

Appendix Table S2. Parameter settings used to establish the correspondence between statistical and dynamic mechanistic model parameters in Fig. 2C.

	k_{on}^{X}	k_{on}^{Y}	k_{off}^{X}	k_{off}^{Y}	k_{syn}^{X}	k_{syn}^{Y}	k_{deg}^{X}	k_{deg}^{X}	$\frac{r_X}{K_X}$	$\frac{r_Y}{K_Y}$	h_X	h_Y	ε_X	ε_Y
c1	10^{-2}~10^{1}	1.2	1	1	15	20	0.8	1.2	2	1	1	0	0.05	0.02
c2	0.8	1	10^{-2}~10^{1}	1	15	20	0.8	1.5	2	1	-1	0	0.05	0.05
c3	0.1	0.5	0.1	0.1	10~30	20	0.8	1.2	2	1	2	0	0.02	0.05

Appendix Table S3. Literature-supported gene regulatory interactions used for biological validation of the BurstLink-inferred subnetwork.

Gene name1	Gene name2	Regulation type1	Regulation type2	PMID
Cebpb	*Fos*	Positive	Positive	11151091
Cebpb	*Hmga1*	Positive	Positive	12665574
Cebpb	*Jun*	Regulation	Regulation	18026136
Cebpb	*Myc*	Positive	Regulation	20581311
Cebpb	*Rela*	Regulation	Regulation	12667216
Cebpb	*Srf*	Positive	Positive	11151091
Cebpb	*Stat3*	Regulation	Regulation	20581311
Cebpb	*Yy1*	Regulation	Regulation	8892889
Egr1	*Fos*	Regulation	Regulation	1909429

Egr1	*Jun*	——	Positive	18281687
Egr1	*Max*	Negative	——	19032775
Egr1	*Myc*	Regulation	Regulation	25147211
Egr1	*Srf*	——	Positive	2040600
Egr1	*Stat3*	——	Positive	25012664
Fos	*Hmga1*	Positive	——	27882471
Fos	*Jun*	Regulation	Regulation	1904542, 3141060
Fos	*Myc*	Regulation	Regulation	21135252
Fos	*Rela*	Regulation	Regulation	12667216
Fos	*Srf*	——	Positive	2453393, 3071491
Fos	*Stat3*	——	Positive	12600988
Fos	*Yy1*	——	Negative	7769693, 8276234
Hmga1	*Jun*	Positive	——	26980699
Hmga1	*Myc*	Positive	——	23184673
Hmga1	*Rela*	Regulation	——	27025651
Hmga1	*Stat3*	Regulation	Regulation	19074878
Jun	*Myc*	Positive	——	21135252
Jun	*Rela*	Regulation	Regulation	12667216
Jun	*Srf*	Regulation	Regulation	10318842
Jun	*Stat3*	Regulation	Regulation	20516108
Jun	*Yy1*	——	Regulation	7935475
Max	*Myc*	Positive	Positive	1501888, 8425218
Myc	*Nfe2l2*	Positive	——	33782124
Myc	*Rela*	——	Positive	8289784, 1114727
Myc	*Stat3*	——	Positive	9874564, 20581311
Myc	*Yy1*	Negative	Negative	9696045, 10858544
Nfe2l2	*Rela*	Negative	Negative	18241676
Nfe2l2	*Stat3*	Regulation	Regulation	32336239
Rela	*Stat3*	Regulation	Regulation	22466650
Rela	*Yy1*	Positive	——	17466695
Srf	*Yy1*	——	Positive	8276234
Stat3	*Yy1*	Positive	Negative	22190092, 33300076

Note: *Positive* indicates that published studies support a positive regulatory effect (activation). *Negative* indicates that published studies support a negative regulatory effect (repression or inhibition). *Regulation* indicates that published evidence supports the existence of a regulatory relationship between the two genes, but the direction or sign of regulation remains unclear. — indicates that no direct experimental evidence was identified supporting a regulatory relationship between the two genes.

Appendix Table S4: The inferred regulation loop type verified by publications.

Gene name1	Gene name2	Base-GRN regulation type1	Base-GRN regulation type2	Inferred regulation type1	Inferred regulation type2	PMID
Hdac2	*Id3*	1.0	1.0	Positive	Positive	30207412, 33273801
Hdac2	*Mbd2*	1.0	1.0	Positive	Positive	10773092
Eno1	*Trp53*	1.0	0.0	Positive	None	10973245
Atf5	*Smarcc1*	0.0	1.0	None	Positive	29137451
Max	*Ybx1*	1.0	1.0	Positive	Negative	36949044
Fos	*Hdac2*	1.0	1.0	Negative	Negative	22496552, 32145408
Hmga1	*Ybx1*	0.0	1.0	None	Negative	-
Atf6	*Elf4*	0.0	0.0	None	None	-

References

Cao Z, Grima R (2020) Analytical distributions for detailed models of stochastic gene expression in eukaryotic cells. Proc Natl Acad Sci USA 117: 4682-4692

Gillespie DT (1976) A general method for numerically simulating the stochastic time evolution of coupled chemical reactions. J Comput Phys 22: 403-434

Hernández-Bastida A, Fernández-Sánchez MP (2012) A Sarmanov family with beta and gamma marginal distributions: an application to the Bayes premium in a collective risk model. Statistical Methods & Applications 21: 391-409

Kamimoto K, Stringa B, Hoffmann CM, Jindal K, Solnica-Krezel L, Morris SA (2023) Dissecting cell identity via network inference and in silico gene perturbation. Nature 614: 742-751

Kim JK, Marioni JC (2013) Inferring the kinetics of stochastic gene expression from single-cell RNA-sequencing data. Genome Biol 14: R7

Liu Y, Beyer A, Aebersold R (2016) On the dependency of cellular protein levels on mRNA abundance. Cell 165: 535-550

Peccoud J, Ycart B (1995) Markovian modeling of gene-product synthesis. Theor Popul Biol 48: 222-234

Sarabia JM, Gomez-Deniz E (2011) Multivariate Poisson-Beta distributions with applications. Communications in Statistics-Theory and Methods 40: 1093-1108

Schwanhäusser B, Busse D, Li N, Dittmar G, Schuchhardt J, Wolf J, Chen W, Selbach M (2011) Global quantification of mammalian gene expression control. Nature 473: 337-342

Tang W, Jørgensen ACS, Marguerat S, Thomas P, Shahrezaei V (2023) Modelling capture efficiency of single-cell RNA-sequencing data improves inference of transcriptome-wide burst kinetics. Bioinformatics 39: btad395

Tian T, Burrage K (2006) Stochastic models for regulatory networks of the genetic toggle switch. Proc Natl Acad Sci USA 103: 8372-8377

Vu TN, Wills QF, Kalari KR, Niu N, Wang L, Rantalainen M, Pawitan Y (2016) Beta-Poisson model for single-cell RNA-seq data analyses. Bioinformatics 32: 2128-2135
