# Supplementary material for: Deciphering global transcriptional dynamics coordinated by gene-gene regulatory interactions using single-cell data
Source: Mol Syst Biol. 2026 Aug 3;22(11):1747–70. doi: 10.1038/s44320-026-00235-4 (PMC13728105; doi:10.1038/s44320-026-00235-4)
Supplement: Supplementary file 2 [file 44320_2026_235_MOESM2_ESM.pdf]

molecular systems biology

Deciphering global transcriptional dynamics coordinated by gene-gene regulatory interactions using single-cell data

Liying Zhou, Songhao Luo, Zhiwei Huang, Zhenquan Zhang, Zihao Wang, and Jiajun Zhang

Corresponding author(s): Jiajun Zhang (zhjiajun@mail.sysu.edu.cn)

Review Timeline:

Submission Date:	7th Jan 26
Editorial Decision:	11th Mar 26
Revision Received:	7th Jun 26
Editorial Decision:	3rd Jul 26
Revision Received:	6th Jul 26
Accepted:	7th Jul 26

Editor: Jingyi Hou

Transaction Report:

(Note: With the exception of the correction of typographical or spelling errors that could be a source of ambiguity, letters and reports are not edited. Depending on transfer agreements, referee reports obtained elsewhere may or may not be included in this compilation. Referee reports are anonymous unless the Referee chooses to sign their reports.)

11th Mar 2026

Manuscript Number: MSB-2026-13561
Title: Deciphering global transcriptional dynamics coordinated by gene-gene regulatory interactions using single-cell data
Author: Liying Zhou
Songhao Luo
Zhiwei Huang
Zhenquan Zhang
Zihao Wang
Jiajun Zhang

Dear Prof Zhang,

Thank you for submitting your work to Molecular Systems Biology. First, please accept my apologies for the delay in sending a decision, which was due to the late arrival of reviewer reports. We have now received reports from two of the three reviewers; unfortunately, despite multiple reminders, we were unable to secure a report from Reviewer #1. To avoid further delay, we are making a decision based on the available reports.

The reviewers find the general topic of your study to be of interest and relavant. However, they have raised substantial concerns regarding both the conceptual and technical aspects of the work. At this stage, the manuscript cannot be considered for publication.

Addressing the reviewers' concerns in full will be necessary for further consideration and this appears to require a lot of additional work. I am unsure whether you will be able or willing to address those and return a revised manuscript. On the other hand, given the potential interest of the study, we would be willing to consider a revised manuscript with the understanding that the reviewers' concerns must be fully addressed and that acceptance of the manuscript would entail a second round of review.

As you probably know, it is Molecular Systems Biology policy to allow a single round of revision only and that, therefore, acceptance or rejection of the manuscript will depend on the completeness of your responses included in the next, final version of the manuscript. For this reason, and to save you from any frustrations in the end we would strongly advise against returning an incomplete revision and would also understand your decision if you choose to rather seek rapid publication elsewhere at this stage.

Should you find that the requested revisions are not feasible within the constraints outlined here and choose, therefore, to submit your paper elsewhere, we would welcome a message to this effect.

On a more editorial level, we would ask you to address the following issues:

- Please provide a .docx formatted version of the manuscript text (including legends for main figures, EV figures and tables). Please make sure that the changes are highlighted to be clearly visible.

- Please provide individual production quality figure files as .eps, .tif, .jpg (one file per figure). When assembling figures, please refer to our figure preparation guideline in order to ensure proper formatting and readability in print as well as on screen:
https://media.springernature.com/original/springer-cms/rest/v1/content/27825798/data/v1

-Please provide a .docx formatted letter INCLUDING the reviewers' reports and your detailed point-by-point responses to their comments. As part of the EMBO Press transparent editorial process, the point-by-point response is part of the Review Process File (RPF), which will be published alongside your paper.

-Please note that all corresponding authors are required to supply an ORCID ID for their name upon submission of a revised manuscript.

-We replaced Supplementary Information with Expanded View (EV) Figures and Tables that are collapsible/expandable online. A maximum of 5 EV Figures can be typeset. EV Figures should be cited as 'Figure EV1, Figure EV2" etc... in the text and their respective legends should be included in the main text after the legends of regular figures.

Additional Tables/Datasets should be labeled and referred to as Table EV1, Dataset EV1, etc. Legends have to be provided in a separate tab in case of .xls files. Alternatively, the legend can be supplied as a separate text file (README) and zipped together with the Table/Dataset file.

For the figures and tables that you do NOT wish to display as Expanded View figures, they should be bundled together with their

legends in a single PDF file called *Appendix*, which should start with a short Table of Content. Each legend should be below the corresponding Figure/Table in the Appendix. Appendix figures and tables should be referred to in the main text as: "Appendix Figure S1, Appendix Figure S2, Appendix Table S1" etc. See detailed instructions regarding expanded view here: https://link.springer.com/journal/44320/submission-guidelines#cms-Expanded-View-data.

-Before submitting your revision, primary datasets (and computer code, where appropriate) produced in this study need to be deposited in an appropriate public database.
Please remember to provide a reviewer password if the datasets are not yet public.
The accession numbers and database should be listed in a formal "Data Availability" section (placed after Materials & Method) that follows the model below (see also https://link.springer.com/partners/embo-press/editorial-policies#Data%20availability%20statement). Please note that the Data Availability Section is restricted to new primary data that are part of this study.

Data availability
The datasets (and computer code) produced in this study are available in the following databases:
- RNA-Seq data: Gene Expression Omnibus GSE46843 (https://www.ncbi.nlm.nih.gov/geo/query/acc.cgi?acc=GSE46843)
- [data type]: [name of the resource] [accession number/identifier/doi] ([URL or identifiers.org/DATABASE:ACCESSION])
*** Note - All links should resolve to a page where the data can be accessed. ***

- At EMBO Press we ask authors to provide source data for the main manuscript figures. You will receive a separate email with instructions for providing source data with your revised manuscript, including how to upload and organize the files.
Additional information on source data and instruction on how to label the files are available <
https://link.springer.com/journal/44320/submission-guidelines#cms-Source-data >

- Our journal encourages inclusion of *data citations in the reference list* to directly cite datasets that were re-used and obtained from public databases. Data citations in the article text are distinct from normal bibliographical citations and should directly link to the database records from which the data can be accessed. In the main text, data citations are formatted as follows: "Data ref: Smith et al, 2001". In the Reference list, data citations must be labeled with "[DATASET]". A data reference must provide the database name, accession number/identifiers and a resolvable link to the landing page from which the data can be accessed at the end of the reference. Further instructions are available at < https://link.springer.com/journal/44320/submission-guidelines#cms-Reference-guidelines >.

- We updated our journal's competing interests policy in January 2022 and request authors to consider both actual and perceived competing interests. Please review the policy (https://link.springer.com/partners/embo-press/editorial-policies#Competing%20interest%20disclosures) and update your competing interests if necessary.
Please use the heading "Disclosure statement and competing interests".

- All Materials and Methods need to be described in the main text using our 'Structured Methods' format. According to this format, the Methods section includes a Reagents and Tools Table (listing key reagents, experimental models, software and relevant equipment and including their sources and relevant identifiers) followed by a Methods and Protocols section describing the methods, ideally using a step-by-step protocol format. The aim is to facilitate adoption of the methodologies across labs.

Please download and fill our Reagents and Tools Table template (.docx), which you can find in our author guidelines: https://link.springer.com/journal/44320/submission-guidelines#structuredmethods.

When submitting your revised manuscript, please do not include the Reagents and Tools Table in the Methods section of the manuscript but upload it as a separate file choosing the file type "Reagent Table".

-Regarding data quantification:
Please ensure to specify the name of the statistical test used to generate error bars and P values, the number (n) of independent experiments (please specify technical or biological replicates) underlying each data point and the test used to calculate p-values in each figure legend. Discussion of statistical methodology can be reported in the materials and methods section, but figure legends should contain a basic description of n, P and the test applied.
Graphs must include a description of the bars and the error bars (s.d., s.e.m.).
Please also include scale bars in all microscopy images.

- Please provide a "standfirst text" summarizing the study in one or two sentences (approximately 250 characters, including space), three to four "bullet points" highlighting the main findings and a " visual abstract" (550px width and 400-600 px height, PNG format) to highlight the paper on our homepage.
Please refer to published papers for examples.

When you resubmit your manuscript, please download our CHECKLIST (https://media.springernature.com/original/springer-cms/rest/v1/content/27825796/data/v1) and include the completed form in your submission.
Please note that the Author Checklist will be published alongside the paper as part of the transparent process .

If you feel you can satisfactorily deal with these points and those listed by the reviewers, you may wish to submit a revised version of your manuscript. Please attach a covering letter giving details of the way in which you have handled each of the points raised by the reviewers. A revised manuscript will be once again subject to review and you probably understand that we can give you no guarantee at this stage that the eventual outcome will be favorable.

I look forward to receiving the revised manuscript soon.

Kind regards,
Jingyi

Jingyi Hou, PhD
Senior Editor
Molecular Systems Biology

We realize that it is difficult to revise to a specific deadline. In the interest of protecting the conceptual advance provided by the work, we recommend a revision within 3 months (9th Jun 2026). Please discuss the revision progress ahead of this time with the editor if you require more time to complete the revisions. Use the link below to submit your revision:

https://msb.msubmit.net/cgi-bin/main.plex

IMPORTANT: Please read our guidelines for revised submissions: https://link.springer.com/journal/44320/submission-guidelines#cms-Revised-submissions

*** PLEASE NOTE *** As part of the EMBO Press transparent editorial process initiative (see our Editorial at https://dx.doi.org/10.1038/msb.2010.72), Molecular Systems Biology publishes online a Review Process File with each accepted manuscripts. This file will be published in conjunction with your paper and will include the anonymous referee reports, your point-by-point response and all pertinent correspondence relating to the manuscript. If you do NOT want this File to be published, please inform the editorial office at contact@molsystbiol.org within 14 days upon receipt of the present letter.

Reviewer #2:

Overall this is a well written manuscript on using single-cell RNAseq data to infer gene regulatory networks. Given the increasing amounts of such data across cell types, this can be a useful tool for the community.

One issue is that a lot of literature on single cell inference is missing from this work, and no comparisons to current gold-standard methods are made.

As an example, the phixer method, which gets causality using single-cell snapshot data:

https://pubmed.ncbi.nlm.nih.gov/33313341/

and was used for inference in a very similar bursting model as used here in this paper:

https://pubmed.ncbi.nlm.nih.gov/32325034/

A somewhat oldish review on single-cell inference methods is here:

https://www.sciencedirect.com/science/article/pii/S0958166919301399

At a bare minimum, a detailed discussion on all these methods and a systematic comparison with BurstLink is needed. Without a systematic comparison, it is hard to see the utility of this new approach.

Extrinsic noise from cell size and cell-cycle has to be discussed and taken into account in the synthetic benchmarking. For example, see:

https://www.cell.com/molecular-cell/fulltext/S1097-2765(15)00170-7

It was unclear how the manuscript was able to infer causality from snapshot data. Can the authors provide analytical results using a simple gene network to intuitively guide the reader on this?

There is also mention of single-cell ATAC-seq data. As far as I know, the correlation between expression level and chromatin opening is quite poor at the single-cell level. Can the author comment on this correlation and how ATAC-seq is being used to inform inference without any dynamical data?

Reviewer #3:

Summary
Zhou et al. present BurstLink, a statistical framework that jointly infers gene-gene regulatory interactions and transcriptional bursting kinetics from single-cell RNA-seq data at genome-wide scale. The framework models gene pairs using two telegraph-model marginals (Poisson-Beta distributions) coupled by Sarmanov-Lee dependence, solved via ADMM, and introduces reweighted mutual information (RMI) to quantify directional regulation strength. Applied to mouse embryonic fibroblast multi-omics data, the authors report that (i) target genes show higher burst frequency and expression variability than transcription factors, (ii) stronger TF binding affinity correlates with lower burst frequency and higher burst size, and (iii) positive regulation increases burst frequency and variability whereas negative regulation has the opposite effect.

The paper tackles an important and timely question: how regulatory network architecture shapes transcriptional bursting globally. The statistical framework is conceptually elegant. However, several major concerns regarding biological assumptions, model validation, and presentation clarity need to be addressed before this work is suitable for publication.

Major Comments
1. The entire framework for gene-gene interactions rests on the assumption that "the abundances of mRNA and protein are highly dependent" (Eq. S6 and line 458), with protein levels proportional to mRNA counts via a constant ratio rc. While there is a genome-wide correlation between mRNA and protein abundances at steady state (Liu et al., 2016, cited by the authors), this assumption ignores the well-documented differences in timescales between mRNA half-lives (minutes to hours) and protein half-lives (hours to days). Transcriptional bursting occurs on the timescale of promoter switching (minutes), whereas the TF proteins that mediate the regulation integrate over much longer timescales due to their slow degradation. This timescale separation means that TF protein levels are substantially buffered relative to mRNA fluctuations, and also fluctuations in protein levels will lag changes in mRNA levels. This weakens the direct mRNA to protein proportionality that the model assumes. The authors should discuss this limitation explicitly, justify the parameter rc used throughout the analyses, and ideally test how sensitive their results are to this assumption. It is thus highly unlikely that regulatory interactions between a TF and its targets can be inferred from pairwise RNA count distributions, the authors must show realistic simulations taking into account multiple alleles, mRNA, and proteins levels, obtained from a realistic translation model, with realistic half-lives and sampling models.

2. The scope of "gene-gene regulatory interactions" is narrow and does not account for enhancer-mediated regulation. The model defines gene-gene interactions exclusively as the sequence: mRNA to protein/TF to promoter binding to target gene activation (Eq. 13-14). This neglects enhancer-mediated regulation, which is a dominant mechanism for controlling transcriptional bursting. TF binding at distal enhancers (not just promoters) is a major determinant of burst frequency, as demonstrated by Bartman et al. (2016, Cell), Fukaya et al. (2016, Cell), Mermet, Yeung et al (Genes and Dev 2017) and importantly by Larsson et al. (2019, cited by the other), who showed genome-wide that enhancers primarily control burst frequency while core promoter elements control burst size. Trzaskoma et al. (2024, ref. 31) further demonstrated that factors acting beyond simple TF-promoter binding (Mediator, BRD4, cohesin) have distinct effects on burst frequency versus burst size, and that the mechanism of regulation matters more than its sign. While the scATAC-seq preprocessing identifies both promoter and enhancer regions (Fig. S4A), the mechanistic model itself only considers promoter-level regulation via a single Hill function. The authors should clarify this discrepancy and discuss whether enhancer-mediated effects are implicitly captured by the statistical model or fundamentally outside its scope. Additionally, it appears that the scATAC-seq data used for base-GRN construction comes from pre-built reference data (CellOracle datasets), not from matched multi-omic profiling of the same cells. Could the authors comment on the feasibility and potential benefit of using matched multi-ome data?

3. The Larsson et al. (2019) dataset was specifically designed to provide allele-resolved expression counts (CAST x C57), which is essential for accurately inferring burst kinetics from scRNA-seq data. Allele-specific counts avoid the confounding effect of summing two independently bursting alleles, each of which follows its own telegraph process. Larsson et al. performed inference independently on each allele and showed that kinetics inferred for the two alleles are correlated but represent two independent transcriptional processes. The authors of the present manuscript state (line 656-659) that they merge the two alleles "under the assumption that the expression distributions of almost all genes for the CAST and C57 alleles follow similar shapes." This merging doubles the effective cell count number but conflates two independent transcriptional processes, artificially inflating

expression levels and altering the count distribution in ways that can bias burst parameter inference. The sum of two independent Poisson-Beta random variables does not itself follow a Poisson-Beta distribution, which is the distribution assumed by the model. The authors need to justify why they depart from this standard practice. Most importantly, Larsson et al. provide published allele-specific BF/BS values for the exact same cells. BurstLink should benchmark directly against these. Are the bursting kinetics inferred in their joint framework similar to those from methods that model bursting in an allele-specific manner? This comparison is missing from the manuscript. Also the authors should discuss the consequences of sampling noises, as the theoretical Poisson-Beta is not invariant under bi-(multi-)nomial sampling.

4. A central claim is that BurstLink jointly infers regulatory interactions and bursting kinetics. However, the burst parameters are node-level variables that are initialized from single-gene Poisson-Beta fits (Eq. 21-22), and the interaction parameter w_e is edge-specific. In the ADMM framework, the consensus variables are updated by averaging local edge estimates (Eq. 25-26). It is not clear how much the burst parameters actually change from their single-gene initializations after convergence. In other words, do the regulatory interactions meaningfully refine the burst kinetics estimates, or are BF and BS essentially the same as those obtained by existing single-gene tools (e.g., txburst from Larsson et al.)? The authors should (a) quantitatively compare their inferred BF/BS to those from single-gene methods on the same dataset, (b) show the magnitude of parameter shift during ADMM iterations, and (c) demonstrate specific cases where joint inference corrects or improves single-gene estimates. Without this, the added value of the joint framework over a two-step approach (infer bursting first, then infer interactions separately) remains unclear.

5. Despite inferring interactions and bursting jointly, the downstream analyses (Section 2.5, Fig. 5) rely entirely on post-hoc statistical tests (t-tests, Bayesian ridge regression) to establish the relationship between regulatory interactions and burst kinetics. If the model truly captures the coupled dynamics, one would expect the parameter we and the burst parameters to be mechanistically linked within the model itself. Instead, the link between regulation type/strength and BF/BS/CV2 is established only through external statistical analysis of the inferred parameters. This raises a fundamental question: what is the gain of doing both together in a joint model if the relationship between the two must still be discovered through separate post-hoc analyses? It is worth noting that the directionality captured by RMI is also a post-hoc construction from the conditional probabilities, not something emerging from the joint model, since the coupling parameter w is itself symmetric. The authors should articulate more clearly what the joint inference provides that a sequential approach would not.

6. The entire inference is performed on mature (spliced) mRNA counts. Unspliced/spliced read ratios carry substantial information about transcriptional dynamics and are now routinely extracted from scRNA-seq data (La Manno et al., 2018; Bergen et al., 2020). Recent methods for burst kinetics inference (e.g., biVI, Carilli et al., 2024, cited by the author; Gorin et al., 2022) leverage both spliced and unspliced counts to improve parameter identifiability. While the authors mention RNA splicing kinetics as a future direction (line 381), this is a significant limitation given the current state of the field. At minimum, the authors should discuss whether incorporating unspliced counts could improve the identifiability of their model parameters, particularly for separating transcription rate from degradation rate.

7. Lines 111-114 state that the input is scRNA-seq data, with scATAC-seq used for "pre-filtering." The manuscript should be more transparent about whether BurstLink is practically useful with scRNA-seq data alone or whether scATAC-seq is effectively required. Additionally, does BurstLink require UMI counts from plate-based protocols (Smart-seq2, as used in the paper), or can it work with standard 10x Genomics data (higher dropout)? The data requirements and technology compatibility should be stated upfront.

8. To support that the model can infer biologically relevant regulatory interactions, the authors should dissect the network inferred in Fig. 4E and validate it through a thorough analysis of published literature in the relevant cell types.

Minor Comments
1. Fig. 3C panels c1, c3, c4 are not cited in the main text. Multiple supplementary figure panels (e.g., Fig. S5D-E) are referenced inconsistently or not at all. Figures contain an excessive number of panels that are difficult to parse. For instance, Fig. 4 has 14 panels (A-N), with multiple plots per panel, many of which could be moved to the supplement. The double-layer network visualization (Fig. 4J) is particularly unclear: what biological insight can the reader extract from this plot? Consider simplifying the main figures to highlight the key messages and moving supporting panels to the supplement.

2. The manuscript uses numerous acronyms (BF, BS, RMI, CMI, NMI, CV2, GRN, TF, TG, SSA, KS, ROC, AUC, ADMM, etc.) that are often not spelled out in figure legends. Since readers frequently examine figures before reading the full text, all acronyms should be defined upon first use in each figure caption. This is especially important for domain-specific terms like RMI and CMI that appear in Fig. 4F-G without definition in the caption.

3. Line 406: "the any two binary variables" should read "any two variables."

4. In the dynamic mechanistic model (Eq. 13), the degradation rates kdeg are set to 1 throughout in Table S1 and vary (0.8, 1.2, 1.5) in Table S2. Both tables lack descriptive captions, and it is unclear what the different rows represent. The authors should clarify what timescale normalization is implied. Since all rates in the telegraph model are expressed relative to the degradation rate, the absolute values of mRNA and protein half-lives matter for interpreting the biological meaning of the inferred

parameters. This should be discussed.

5.The average capture efficiency is set to 0.3 for Smart-seq2 (line 583). How sensitive are the results to this value? This should be tested, especially since the method claims applicability to different scRNA-seq technologies, which have widely varying capture efficiencies. This is shortly tested in the simulation (Fig S2) but barely described; one sentence for 12 panels. How does the binomial sampling changes the Poisson-Beta model? (See above).

6.The manuscript emphasizes scalability but does not report runtime or memory usage. How long does inference take for the 4311 genes and 291'228 gene pairs? How does this scale with gene number and cell number?

Response to Reviewers

Reviewer #1

Overall this is a well written manuscript on using single-cell RNAseq data to infer gene regulatory networks. Given the increasing amounts of such data across cell types, this can be a useful tool for the community.

Comment 1: One issue is that a lot of literature on single cell inference is missing from this work, and no comparisons to current gold-standard methods are made. As an example, the phixer method, which gets causality using single-cell snapshot data: https://pubmed.ncbi.nlm.nih.gov/33313341/ and was used for inference in a very similar bursting model as used here in this paper: https://pubmed.ncbi.nlm.nih.gov/32325034/. A somewhat oldish review on single-cell inference methods is here: https://www.sciencedirect.com/science/article/pii/S0958166919301399. At a bare minimum, a detailed discussion on all these methods and a systematic comparison with BurstLink is needed. Without a systematic comparison, it is hard to see the utility of this new approach.

Response: We thank you for this important comment. In the revised manuscript, we added a more comprehensive review and citation to existing single-cell GRN inference literature in the Introduction section (Page 2, Line 58-63), including information-theoretic, tree-based, dynamical, and mechanistic approaches. We also performed systematic benchmarking against current methods in terms of accuracy, robustness, scalability and unique methodological properties (Page 9, Line 275-283; Page 30-31, Line 936-947; Appendix Fig. S3; Response Fig. 1.1).

Specifically, we compared BurstLink with several representative single-cell GRN inference methods, including PIDC, Phixer, GENIE3, GRNBoost2, and SCENIC. For benchmarking on biological data, we used the Smart-seq2 scRNA-seq dataset from mouse embryonic fibroblasts (C57 × CAST) [Larsson et al., Nature, 2019]. In each benchmark realization, 30 genes were randomly sampled to construct a fully connected candidate regulatory subnetwork. To set up the ground truth through biologically plausible regulatory interactions, we employed scATAC-seq-derived TF–target priors [Kamimoto et al., Nature, 2023] as an ATAC-supported reference network for performance evaluation. To ensure robust evaluation, the entire benchmarking procedure was independently repeated 100 times using different randomly sampled gene subsets.

As summarized in Response Fig. 1.1, we systematically evaluated these single-cell GRN inference methods from four perspectives: (1) inference accuracy, assessed using AUROC, AUPRC, Precision@k, and normalized AUPRC (AUPRC/baseline), where the baseline precision was defined as the fraction of positive interactions in the reference network; (2) robustness, including performance under varying gene expression dropout levels and network sparsity; (3) computational efficiency, including runtime and parallel scalability; and (4) methodological properties, including mechanistic modeling, inference of transcriptional burst kinetics, and recovery of regulatory directionality. Overall, the benchmark results demonstrate that BurstLink achieves regulatory interaction inference

performance that is comparable to current state-of-the-art GRN inference methods across accuracy, robustness, and computational scalability. In particular, BurstLink consistently ranks among the top-performing methods in terms of AUROC, AUPRC, and Precision@k, while maintaining strong robustness to both expression dropout and network sparsity. Importantly, beyond achieving competitive GRN reconstruction performance, the primary advantage of BurstLink lies in its mechanistic modeling framework, which simultaneously infers transcriptional bursting parameters, regulatory interactions and regulation-dependent conditional expression distributions within a unified framework. This joint modeling strategy accounts for the coordinated effects between gene regulation and transcriptional bursting, thereby enabling mechanistically interpretable characterization of both GRN structure and bursting dynamics, which is not readily available from existing GRN inference methods.

Notably, the study cited by the reviewer [Schuh et al., Cell Systems, 2020] is highly relevant to our work, as it also investigated how transcriptional bursting combined with gene regulatory interactions can generate coordinated expression states. However, the goal of that study was different from ours. Schuh et al. developed a **forward modeling problem** to test whether predefined transcriptional bursting models and network topologies were sufficient to recapitulate rare, transient, coordinated high-expression states observed in melanoma cells. They systematically sampled model parameters and network architectures, and used Gillespie simulations to examine the conditions under which such rare states can arise. We also note that the authors employed the Phixer algorithm in their study. However, Phixer was not used to estimate the kinetic parameters of the transcriptional bursting model. Rather, it was applied as an existing network inference method to RNA-FISH data to assess whether experimentally inferred network connectivity was consistent with the mechanistic predictions and conclusions derived from the simulation framework.

In contrast, BurstLink addresses the **inverse inference problem**. Rather than starting from predefined kinetic parameters and network structures, BurstLink uses snapshot scRNA-seq data to infer underlying transcriptional bursting kinetics and directional gene–gene regulatory interactions. From this perspective, the Cell Systems study and BurstLink are conceptually complementary approaches rather than directly competing approaches. Indeed, the mechanistic bursting framework adopted in the Cell Systems study further supports the biological plausibility of the inference problem addressed by BurstLink. We have clarified this distinction in the revised manuscript Discussion section (Page 17, Line 468-470).

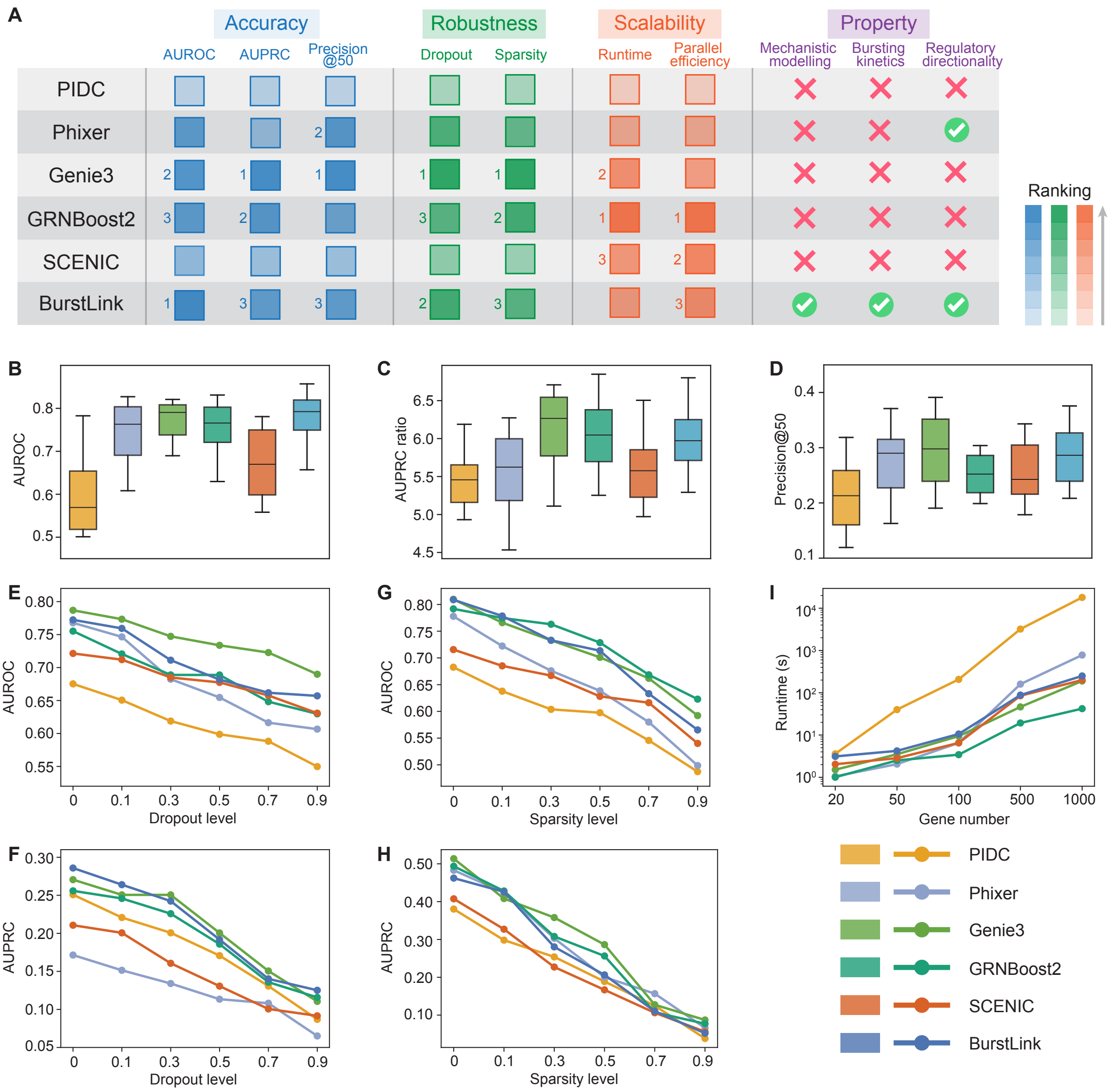

Response Fig. 1.1 | Benchmarking of BurstLink and representative GRN inference methods. (A) Summary of method rankings across accuracy, robustness, scalability, and methodological properties. (B–D) Comparison of inference accuracy using AUROC, AUPRC normalized by baseline, and Precision@50. (E–H) Performance under increasing dropout and sparsity levels measured by AUROC and AUPRC. (I) Runtime comparison across different gene numbers.

Comment 2: Extrinsic noise from cell size and cell-cycle has to be discussed and taken into account in the synthetic benchmarking. For example, see: https://www.cell.com/molecular-cell/fulltext/S1097-2765(15)00170-7

Response: We thank you for this valuable comment. We agree that extrinsic variability associated with cell size and cell-cycle state can affect observed single-cell gene expression distributions and should be considered when evaluating the robustness of bursting-kinetic inference. As noted in previous studies, gene expression is coupled to cell size and is therefore affected by the cell cycle [Battich et al., 2015; Sun et al., 2020]. Thus, cell-size-associated scaling represents one important route

through which cell-cycle progression can influence observed transcript-count distributions. In the revised manuscript, we have added a synthetic benchmarking analysis explicitly incorporating cell-size-associated extrinsic noise (Page 28, Line 856-857; Appendix Text S1.6; Appendix Fig. S17). At the same time, cell cycle can also introduce other phase-specific transcriptional programs and regulatory effects. We also have expanded the Discussion to clarify the broader potential influence of cell-cycle effects (Page 20, Line 571-574).

Specifically, to mimic the effect of cell-size variability on observed transcript counts, we estimated an empirical cell-size factor distribution from a real scRNA-seq dataset using per-cell library size, denoted as $s_k \sim \hat{P}(s)$ (Response Fig. 1.2A). The observed count X_{ik} for gene i in cell k was then sampled as

$$X_{ik} \sim \text{Poisson}\left(s_k \phi_i \eta_i\right), \eta_i \sim \text{Beta}\left(\alpha_i, \beta_i\right) i = 1, 2, ..., n, \quad (1.1)$$

where s_k denotes the cell-size factor. This treatment for cell size is consistent with the recent scRNA-seq burst-inference model [Tang et al., Bioinformatics, 2023] which explicitly represents cell size as a cell-specific multiplicative factor affecting the effective transcription rate and observed transcript-count distribution.

This procedure produced an observed distribution that differed from the original true distribution because of cell-size-associated extrinsic variability (Response Fig. 1.2B). We then applied BurstLink to the observed distribution and inferred the corresponding bursting parameters. Based on the inferred parameters, we reconstructed the observation-space distribution (Response Fig. 1.2C). We used the Kolmogorov–Smirnov test to assess whether the inferred distribution was statistically consistent with the target distribution.

The results show that BurstLink accurately recovered both burst frequency and burst size under this cell-size perturbation setting (Response Fig. 1.2D). To further evaluate robustness, we repeated this simulation independently 100 times. In more than 90% of the simulations, the recovered distributions were statistically consistent with the corresponding target distribution (Response Fig. 1.2E). In addition, the recovered bursting kinetic parameters showed low error across repeated simulations (Response Fig. 1.2F). Together, these results indicate that BurstLink remains robust when cell-size-associated extrinsic noise is explicitly introduced into the synthetic benchmarking procedure.

Finally, we have clarified in the revised Discussion that the current benchmarking accounts for cell-size-associated global transcript scaling, which captures one major component of cell-cycle-associated extrinsic variability. However, cell-cycle-dependent regulation may also introduce additional structured variation in bursting kinetics, such as phase-specific transcriptional programs or cell-cycle-associated regulatory activity. We therefore now discuss explicit modeling of cell-cycle phase or cell-cycle-associated covariates as an important direction for future extensions.

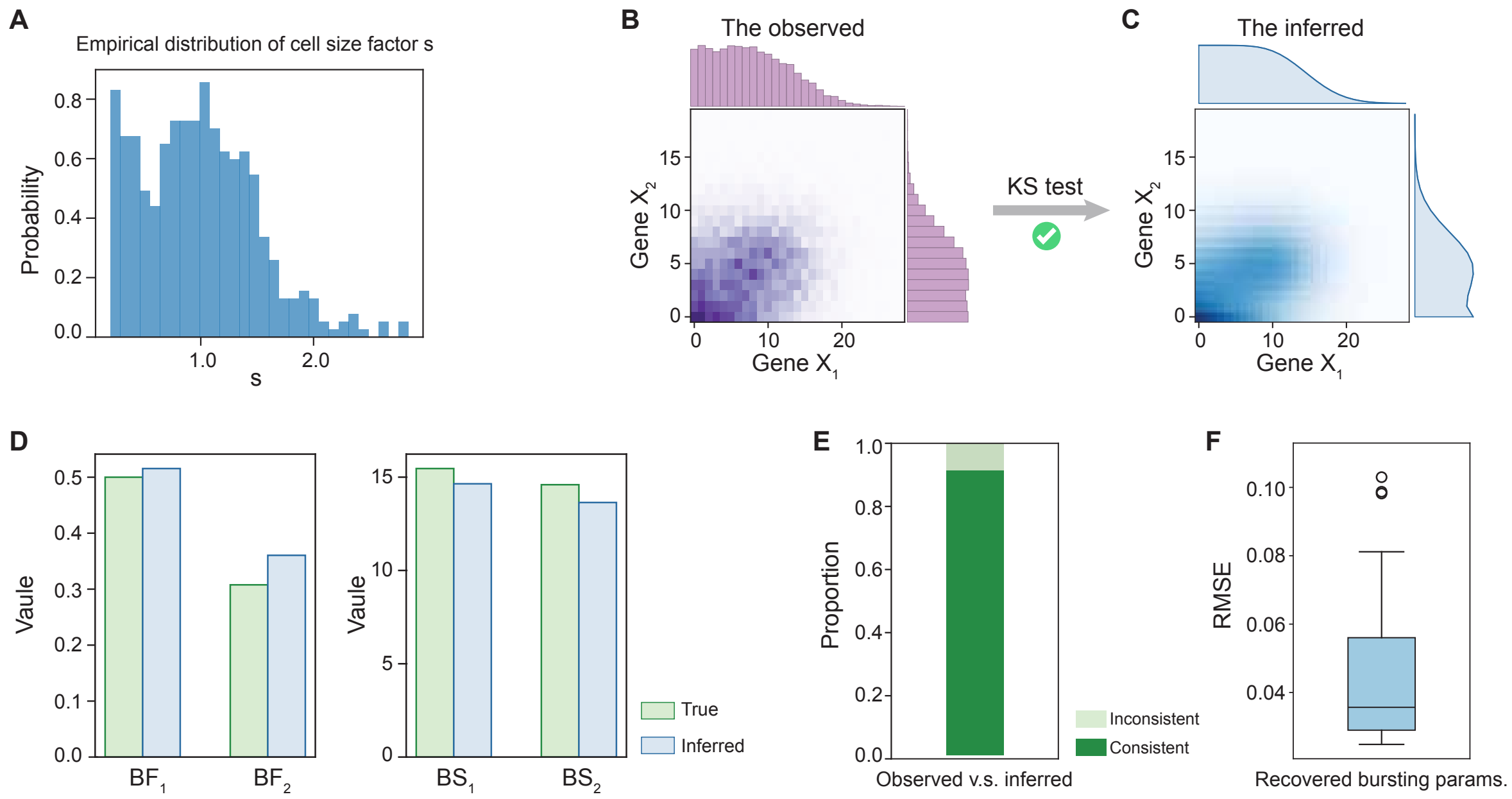

Response Fig. 1.2 | Benchmarking of BurstLink robustness under cell-size-associated extrinsic variability. (A) Empirical distribution of cell-size factors estimated from a real scRNA-seq dataset based on per-cell library size. (B) Observed joint gene-expression distribution generated by introducing cell-size-associated scaling. (C) Joint gene-expression distribution inferred by BurstLink from the observed data shown in (B). (D) Comparison of burst frequency (BF) and burst size (BS) between the ground-truth parameters and the parameters inferred by BurstLink for the representative example shown in (B–C). (E) Proportion of simulations in which the inferred distributions were statistically consistent with the corresponding observed distributions across 100 independent simulations, as assessed by the Kolmogorov–Smirnov test. (F) Distribution of the root mean square error (RMSE) between inferred and ground-truth bursting kinetic parameters across 100 independent simulations.

Comment 3: It was unclear how the manuscript was able to infer causality from snapshot data. Can the authors provide analytical results using a simple gene network to intuitively guide the reader on this?

Response: We thank you for raising this important point. Whether and to what extent causal relationships can be inferred from static snapshot data remains a fundamental question. In the discussion section of this revised manuscript, we have clarified precisely the scope and the limits of the causal interpretation that BurstLink's inferences can support (Page 18-19, Line 516-523). And we have added theoretical analyses to explain how BurstLink infers regulatory directionality from snapshot data by using asymmetric information encoded in steady-state conditional distributions. We also included a simple gene-network example, together with a schematic illustration, to guide readers through the intuition behind this directionality inference (Page 22, Line 638-641; Appendix Text S1.3; Appendix Fig. S12)

First, to be explicit about the scope of these claims, observational snapshot data alone cannot establish causality in the strict interventional sense, which would require perturbation experiments and temporal data; BurstLink therefore does not claim to demonstrate intervention-level causality. What BurstLink infers is a directional dependence between genes extracted from the asymmetry of the steady-state conditional distributions, in the same spirit as observational causal-discovery methods that orient edges from distributional asymmetry rather than from symmetric association. The reweighted mutual information (RMI) score is precisely the measure that operationalizes this asymmetry, distinguishing the possible regulatory directions between genes rather than relying on symmetric measures such as correlation. Based on it, when combined with biologically informed GRN priors [Stock et al., Molecular Systems Biology, 2025] such as TF–target relationships, chromatin accessibility, motif information, or curated regulatory databases, these directional dependencies are further constrained and may be interpreted as biologically plausible, putative causal regulatory relationships.

Second, to provide an intuitive explanation of how directional regulatory information can arise from snapshot data, we added a minimal analytical example based on a simple two-gene regulatory module. Given a two-gene regulatory module $X \rightarrow Y$, gene X is the upstream regulator and gene Y Y is the downstream target gene. Conditional on the expression level of the upstream regulator $X = m$, the downstream gene Y follows a two-state transcriptional bursting model:

$$\begin{aligned} &G_0 \xrightarrow{k_{\text{on}}(m)} G_1,\ G_1 \xrightarrow{k_{\text{off}}} G_0, \\ &G_1 \xrightarrow{s} G_1 + Y,\ Y \xrightarrow{\gamma} \phi. \end{aligned} \tag{1.2}$$

Here, G_0 and G_1 denote the OFF and ON promoter states, respectively. The parameter s is the transcription rate and γ is the mRNA degradation rate. The key assumption is that the upstream regulator modulates the burst activation rate of the downstream gene $k_{\text{on}}(m)$. If $k_{\text{on}}(m)$ is an increasing function of m, then X positively regulates Y; if it is decreasing, X negatively regulates Y.

Let $P_0(n,t \mid m)$ and $P_1(n,t \mid m)$ denote the probabilities that gene Y contains n mRNA molecules while its promoter is in the OFF and ON states, respectively. The corresponding conditional chemical master equations can be written as

$$\begin{cases} \dfrac{dP_0(n,t \mid m)}{dt} = -k_{\text{on}}(m)P_0(n,t \mid m) + k_{\text{off}}P_1(n,t \mid m) + \gamma\left[(n+1)P_0(n+1,t \mid m) - nP_0(n,t \mid m)\right], \\ \dfrac{dP_1(n,t \mid m)}{dt} = k_{\text{on}}(m)P_0(n,t \mid m) - k_{\text{off}}P_1(n,t \mid m) + s\left[P_1(n-1,t \mid m) - P_1(n,t \mid m)\right] \\ \qquad\qquad\qquad + \gamma\left[(n+1)P_1(n+1,t \mid m) - nP_1(n,t \mid m)\right]. \end{cases} \tag{1.3}$$

By introducing the probability generating functions and solving the steady-state master equation, the total generating function can be obtained

$$F(z|m) = {}_1F_1\left(\alpha(m); \alpha(m) + \beta; \phi(z-1)\right), \tag{1.4}$$

where $\alpha(m)=k_{\text{on}}(m)/\gamma, \beta=k_{\text{off}}/\gamma, \phi=s/\gamma$. Using the integral representation of the Kummer function, the total generating function can be written as

$$F(z|m)=\int_0^1 \exp\{\phi\eta(z-1)\}\frac{\eta^{\alpha(m)-1}(1-\eta)^{\beta-1}}{\text{Beta}(\alpha(m),\beta)}d\eta. \tag{1.5}$$

Note that $\exp\{\phi\eta(z-1)\}$ is the probability generating function of a Poisson distribution with mean $\phi\eta$. Therefore, the above expression can be interpreted as a Poisson distribution whose rate parameter is mixed by a Beta distribution, implying that the steady-state distribution follows a Poisson-Beta form

$$\begin{aligned} Y \mid \eta, X=m &\sim \text{Poisson}(\phi\eta), \\ \eta \mid X=m &\sim \text{Beta}(\alpha(m),\beta), \end{aligned} \tag{1.6}$$

or equivalently,

$$P(Y=n \mid X=m)=\int_0^1 \frac{(\phi\eta)^n e^{-\phi\eta}}{n!}\frac{\eta^{\alpha(m)-1}(1-\eta)^{\beta-1}}{\text{Beta}(\alpha(m),\beta)}d\eta. \tag{1.7}$$

This result shows that the upstream regulator directly changes the steady-state conditional distribution of the downstream gene through $\alpha(m)$. Thus, the conditional mean is

$$\text{E}[Y \mid X=m]=\phi\text{E}[\eta \mid X=m]=\frac{s}{\gamma}\frac{k_{\text{on}}(m)}{k_{\text{on}}(m)+k_{\text{off}}}. \tag{1.8}$$

Differentiating with respect to m gives

$$\frac{d\text{E}[Y \mid X=m]}{dm}=\frac{sk_{\text{off}}}{\gamma(k_{\text{on}}(m)+k_{\text{off}})^2}k_{\text{on}}'(m). \tag{1.9}$$

Since $k_{\text{on}}(m)$ is assumed to be monotonic, the sign of the derivative is determined by the sign of $k_{\text{on}}'(m)$. Therefore, changes in the upstream gene expression systematically alter the stationary expression distribution of the downstream gene. Importantly, this analytical example demonstrates that directional regulation enters the generative process asymmetrically $X \xrightarrow{k_{\text{on}}(m)} Y$. This result explains why directional information can be retained in snapshot data, under the assumption that the observed cells are samples from an underlying steady-state distribution, despite the absence of temporal measurements.

Third, to further improve intuition, we additionally included a simple three-gene network example (Response Fig. 1.3). This example explicitly illustrates how BurstLink transforms inferred conditional probability distributions into regulation response curves and subsequently infers regulatory type and directionality between gene pairs from snapshot data.

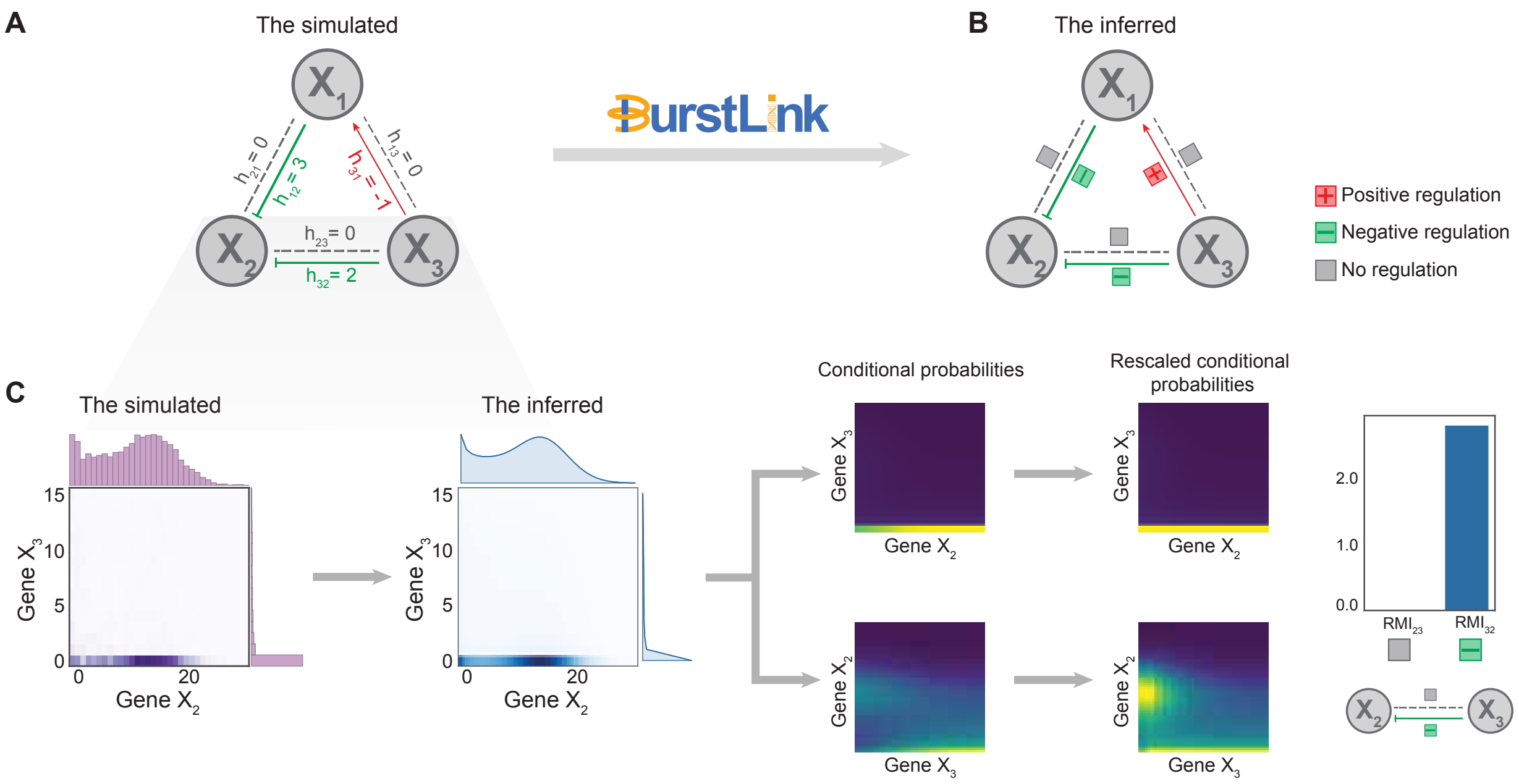

Response Fig. 1.3 | Analytical illustration of directional regulation inference from snapshot data. (A) Simulated three-gene regulatory network used as an example. Red arrows denote positive regulation, green arrows denote negative regulation, and gray lines indicate the absence of direct regulation. (B) Regulatory network inferred by BurstLink from the simulated snapshot gene expression data. The inferred regulatory interactions accurately recover the underlying network structure, including both regulatory directionality, regulation types and regulation strengths. (C) Illustration of the inference procedure for a representative gene pair. Starting from simulated snapshot single-cell expression data, BurstLink first reconstructs the underlying steady-state gene expression distribution. Conditional probability distributions and rescaled conditional probability distributions are then derived from the inferred distribution, from which regulation response curves and regulatory interaction statistics are calculated. These statistics are subsequently used to infer the regulatory type, directionality, and strength of the interaction between the two genes.

Comment4: There is also mention of single-cell ATAC-seq data. As far as I know, the correlation between expression level and chromatin opening is quite poor at the single-cell level. Can the author comment on this correlation and how ATAC-seq is being used to inform inference without any dynamical data?

Response: We thank you for raising this concern. We fully agree that, at the single-cell level, the correlation between chromatin accessibility and expression level of the same genes is often weak, which has been widely reported. However, this weak correlation does not conflict with our use of scATAC-seq, which is employed not as a proxy for gene expression but solely to identify structurally plausible candidate TF–target pairs based on chromatin accessibility and TF motif enrichment in putative regulatory regions (Page 32, Line 992-996; Appendix Fig. S20; Response Fig. 1.4).

In our framework, scATAC-seq is used only to define a biologically plausible candidate regulatory space for downstream inference (Response Fig. 1.4A). Specifically, for a given target gene (TG), we first identify accessible chromatin peaks located in its putative regulatory regions, including promoters and enhancers (Response Fig. 1.4B). We then extract the DNA sequences underlying these peaks and perform TF motif analysis (Response Fig. 1.4C). If a motif associated with a particular transcription factor (TF) is enriched within an accessible region, that TF is considered a candidate regulator of the target gene (Response Fig. 1.4D). Thus, scATAC-seq provides structural regulatory priors, indicating that a TF–TG interaction is biologically plausible, rather than quantitative estimates of transcriptional activity, regulatory strength, or kinetic parameters. This use of chromatin accessibility as a regulatory prior is conceptually aligned with CellOracle [Kamimoto et al., Nature, 2023].

The inference of regulatory strength and transcriptional bursting kinetics is subsequently performed using scRNA-seq data through our mechanistic model. Although snapshot scRNA-seq data do not provide direct dynamics, they contain distributional information shaped by stochastic transcriptional bursting, such as expression variability, zero inflation, and higher-order distributional features. BurstLink uses this information to infer the kinetic parameters that best explain the observed single-cell expression distributions under our statistical mechanistic model. Therefore, our method does not infer dynamical behavior directly from scATAC-seq, nor does it require scATAC-seq to encode dynamics. Instead, scATAC-seq constrains the candidate regulatory TF-TG pairs, while scRNA-seq drives the quantitative inference of bursting kinetics and regulatory interactions.

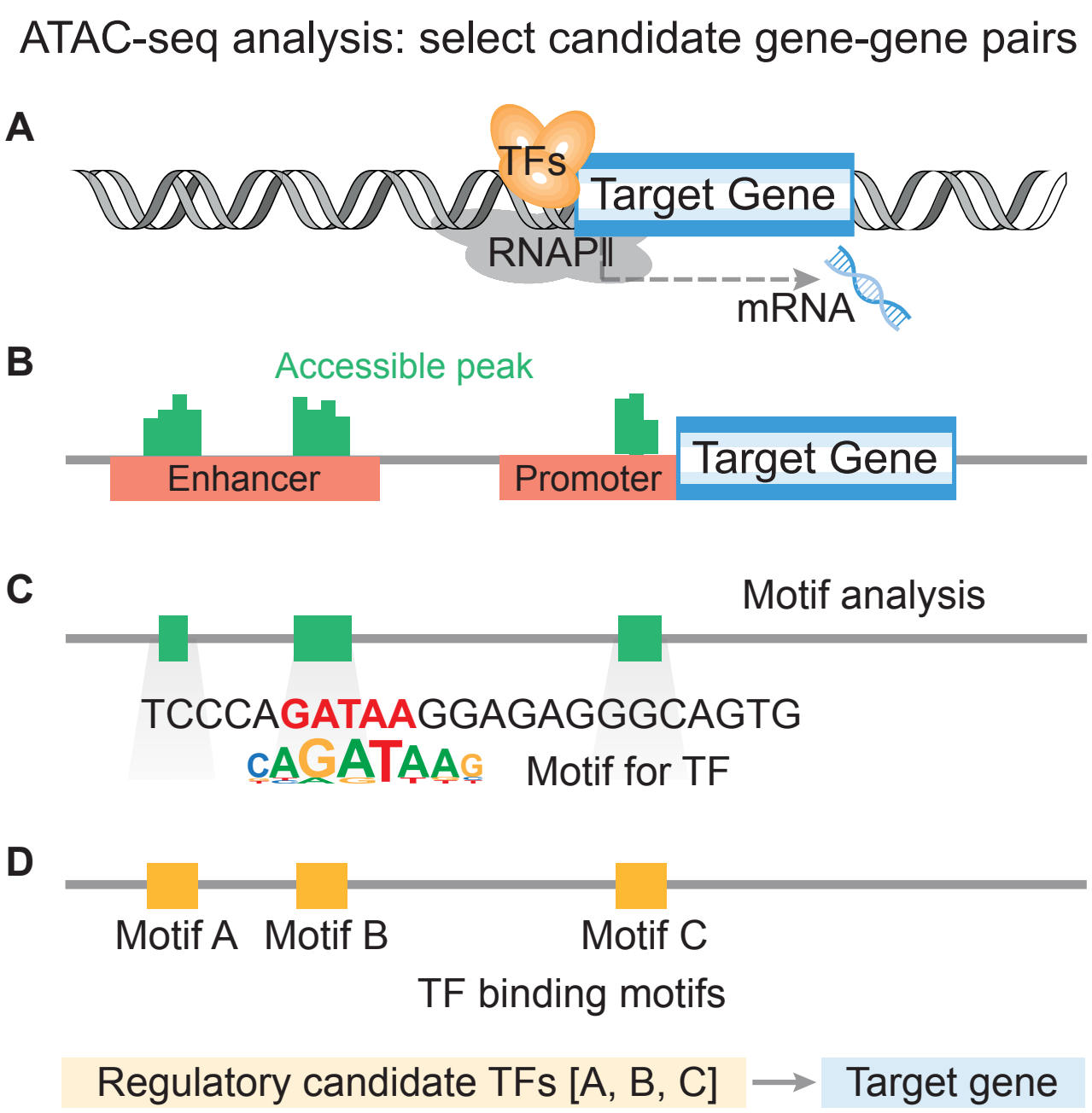

Response Fig. 1.4 | Selection of candidate TF-TG pairs using scATAC-seq data. (A) Schematic of TF-mediated regulation of a target gene (TG). (B) Identification of accessible chromatin peaks in putative regulatory regions of the TG, including promoters and enhancers. (C) Extraction of DNA

sequences from accessible peaks and analysis of TF binding motifs. (D) Selection of candidate TFs whose motifs are detected in accessible regulatory regions of the TG.

Reviewer #2

Summary

Zhou et al. present BurstLink, a statistical framework that jointly infers gene-gene regulatory interactions and transcriptional bursting kinetics from single-cell RNA-seq data at genome-wide scale. The framework models gene pairs using two telegraph-model marginals (Poisson-Beta distributions) coupled by Sarmanov-Lee dependence, solved via ADMM, and introduces reweighted mutual information (RMI) to quantify directional regulation strength. Applied to mouse embryonic fibroblast multi-omics data, the authors report that (i) target genes show higher burst frequency and expression variability than transcription factors, (ii) stronger TF binding affinity correlates with lower burst frequency and higher burst size, and (iii) positive regulation increases burst frequency and variability whereas negative regulation has the opposite effect. The paper tackles an important and timely question: how regulatory network architecture shapes transcriptional bursting globally. The statistical framework is conceptually elegant. However, several major concerns regarding biological assumptions, model validation, and presentation clarity need to be addressed before this work is suitable for publication.

Major Comments

Comment 1: The entire framework for gene-gene interactions rests on the assumption that "the abundances of mRNA and protein are highly dependent" (Eq. S6 and line 458), with protein levels proportional to mRNA counts via a constant ratio r_c. While there is a genome-wide correlation between mRNA and protein abundances at steady state (Liu et al., 2016, cited by the authors), this assumption ignores the well-documented differences in timescales between mRNA half-lives (minutes to hours) and protein half-lives (hours to days). Transcriptional bursting occurs on the timescale of promoter switching (minutes), whereas the TF proteins that mediate the regulation integrate over much longer timescales due to their slow degradation. This timescale separation means that TF protein levels are substantially buffered relative to mRNA fluctuations, and also fluctuations in protein levels will lag changes in mRNA levels. This weakens the direct mRNA to protein proportionality that the model assumes. The authors should discuss this limitation explicitly, justify the parameter r_c used throughout the analyses, and ideally test how sensitive their results are to this assumption. It is thus highly unlikely that regulatory interactions between a TF and its targets can be inferred from pairwise RNA count distributions, the authors must show realistic simulations taking into account multiple alleles, mRNA, and proteins levels, obtained from a realistic translation model, with realistic half-lives and sampling models.

Response: Thank you for raising this important point regarding the interpretation of r_c. We agree that interpreting r_c as an instantaneous protein-to-mRNA ratio would overlook the timescale differences between mRNA and protein dynamics. In response, we have (i) revised the Methods to reinterpret r_c ·

m not as protein abundance but as an effective, phenomenological regulatory proxy for input that enters the model through the Hill term (Page 23, Line 683-690); (ii) added an explicit discussion of this limitation in the Discussion (Page 19, Line 537-544); and (iii) performed two new analyses to test robustness — a sensitivity analysis varying r_c (Response Fig. 2.1; Page 23, Line 683-690; Appendix Fig. S1.4; Appendix Fig. S13), and a new simulation that explicitly incorporates protein dynamics with a realistic mRNA–protein timescale separation and time delay (Response Fig. 2.2; Page 19, Line 539-544; Appendix Text S1.4; Appendix Fig. S11). We elaborate on each point below.

First, we clarify the structure of the toggle-switch model used here. The model explicitly describes gene switching between inactive and active states together with mRNA production and degradation, and gene regulation enters only through the Hill term that modulates the switching rate. The model therefore contains no explicit translation, protein synthesis, or protein-degradation layer; its central object is mRNA-level transcriptional bursting. This is the context in which the input to the Hill term must be interpreted.

Moreover, the path from a regulator's transcript to its actual regulatory activity on a target gene is biologically complex: it involves translation, protein turnover, post-translational modification, nuclear localization, cofactor binding, and DNA binding, among other steps. Our model does not attempt to reconstruct this multi-step process mechanistically, nor does it reduce it to a single protein synthesis–degradation system. Accordingly, the quantity entering the Hill term is best understood as an *effective regulatory input* — a coarse-grained proxy for the (unobserved) regulatory activity contributed by the TF gene — rather than an instantaneous TF protein abundance.

Second, because the full transcript-to-activity pathway is neither observable from scRNA-seq nor the object we aim to model, we require a proxy that depends only on the observable regulator mRNA. We adopt the most parsimonious, assumption-light choice: a monotonic relationship in which a higher regulator transcript level corresponds to a stronger regulatory input, using the simplest first-order form $\mathrm{f(m)} = \mathrm{r_c} \cdot \mathrm{m}$, which is the leading-order term of a general monotonic mapping. We therefore no longer interpret $r_c \cdot m$ as protein abundance; it is a minimal phenomenological mapping. Importantly, this effective input is not assumed to act without bound: it is passed through a Hill function, so that thresholding, saturation, and cooperative-type responses are captured by the downstream nonlinearity. The principal limitation of this choice is that $f(m) = r_c \cdot m$ does not explicitly capture protein turnover, time delays, or other processes that can decouple transcript abundance from regulatory activity.

Third, we further note that this is not a limitation specific to our BurstLink but a constraint shared by scRNA-seq-based regulatory inference. Because scRNA-seq measures transcript abundance and does not report protein levels or TF activity, any method that infers gene regulation from scRNA-seq must necessarily use transcript abundance as a proxy for the underlying regulatory activity. This assumption underlies widely used approaches: SCENIC defines regulons from the co-expression of

TF transcripts with their putative target genes [Aibar et al., Nature Methods, 2017]; CellOracle predicts target-gene expression using regularized linear models of TF transcript levels [Kamimoto et al., Nature, 2023]. More broadly, the use of TF transcript abundance as a proxy for TF activity, together with its limitations, has been explicitly recognized in the single-cell GRN literature [Badia-i-Mompel et al., Nature Reviews Genetics, 2023]. That these methods recover biologically meaningful regulatory relationships indicates that informative regulatory structure is recoverable from transcript-level data despite this shared assumption.

Consistent with this, our own results provide direct empirical support. When benchmarked against an orthogonal chromatin-accessibility base-GRN, BurstLink recovers regulatory interactions from pairwise RNA distributions with high accuracy (AUC = 0.85, Fig. 4B), and it continues to do so even without any scATAC prior (AUC = 0.72–0.82, Fig. 4C). The inferred interactions are further consistent with independently validated regulatory pairs (e.g., *Srf–Stat3*, *Cebpb–Hmga1*) and are largely conserved across mouse embryonic stem cells and fibroblast (Fig. S8), indicating that the recovered structure reflects genuine regulatory relationships.

Fourth, to directly address the concern regarding the value of r_c, we performed a sensitivity analysis by systematically varying r_X over a 16-fold dynamic range (1.75–28), generating synthetic data using the toggle-switch model SSA, and re-running the full BurstLink inference pipeline. Across the entire range examined, the recovery rate remained consistently high for both regulation types (0.80–0.94 for positive and 0.78–0.96 for negative regulation). These results demonstrate that the recovery of regulation direction and type is robust to the specific value of r_X.

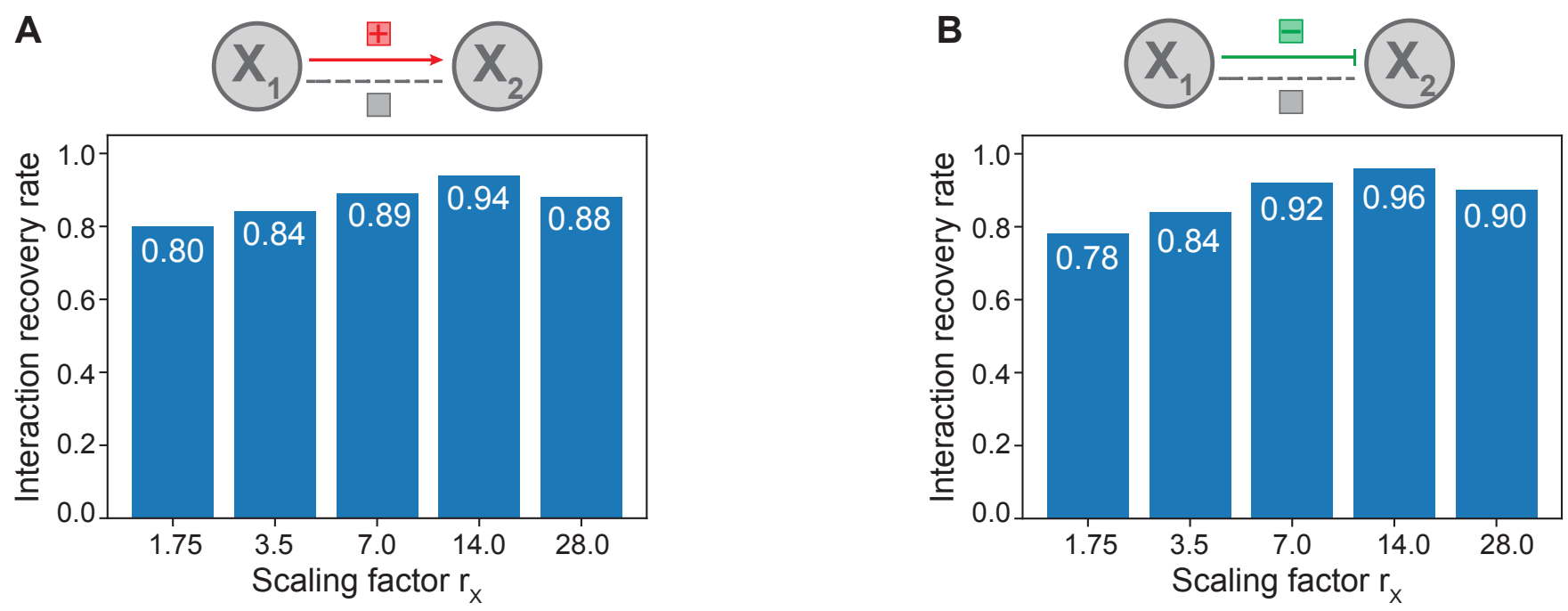

Response Fig. 2.1 | Sensitivity of BurstLink inference to r_X. Synthetic data were generated from the toggle-switch model (X_1 regulates X_2; reverse direction non-regulation) with r_X varied over a dynamic range, and BurstLink was re-run at each setting. The interaction recovery rate is the fraction of cases where both the direction and type of regulation were correctly identified. **(A)** Positive and **(B)** negative regulation. Recovery stays high across the full range (0.78–0.96).

Fifth, to directly address the simulation incorporating explicit protein dynamics, we extended the toggle-switch model to include the TF's protein species, so that regulation is mediated by protein rather than by mRNA, with protein degradation rate = 1/4 of the mRNA degradation rate [Schwanhäusser et

al., Nature, 2011]. This explicitly introduces the buffering and time delay between transcript and regulatory activity. We then compared inference under this protein-delay model against the original model, for both positive (Response Fig. 2.2A–G) and negative (Response Fig. 2.2H–N) regulation. As expected, the protein trajectories are slower and buffered relative to mRNA (Response Fig. 2.2C, J). Nevertheless, BurstLink still correctly recovers the qualitative regulatory relationship — both the type (positive/negative) and the direction — under the protein-delay model: the rescaled conditional-probability heatmaps continue to display the correct positive (F, G) or negative (M, N) trend with and without protein delay. While slow protein turnover attenuates the inferred coupling relative to the instantaneous case, it does not abolish all the regulatory signal in the stationary pairwise mRNA distribution, supporting the use of regulator mRNA as a proxy for recovering the type and direction of regulation even when the true mediator is a long-lived protein. As for the simulation of multiple alleles, allele-resolved modeling lies outside the scope of the current BurstLink framework, we discuss this point further in our response to Comment 2.3.

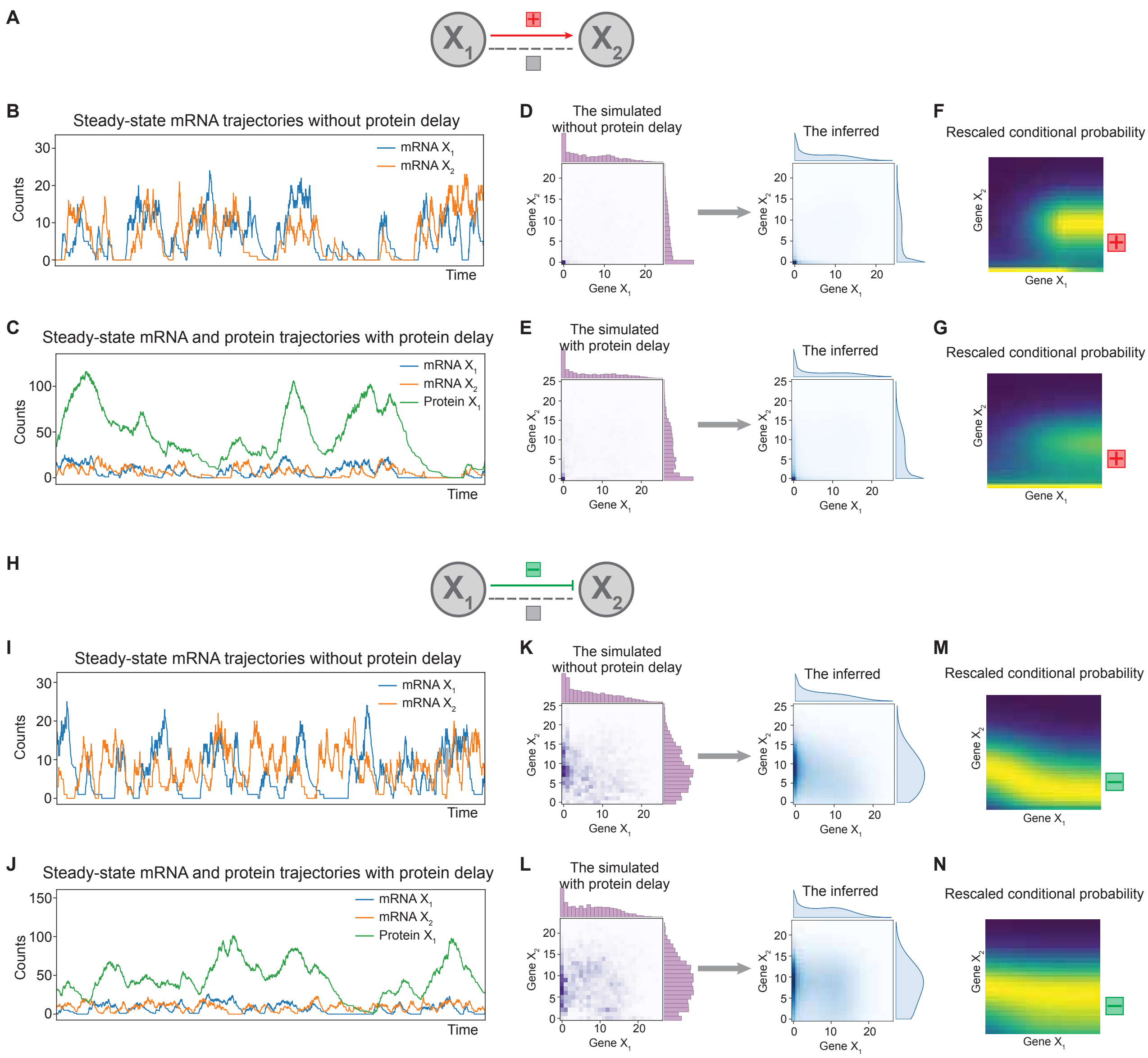

Response Fig. 2.2 | BurstLink recovers regulation under explicit protein dynamics with time delay. The toggle-switch model was extended so that regulation is mediated by the TF's protein, with protein degrading 4× slower than mRNA. (A, H) Ground-truth schematics: X_1 positively (A) or negatively (H) regulates X_2; reverse direction non-regulation. (B, I) Steady-state mRNA trajectories without protein delay; (C, J) mRNA and protein trajectories with protein delay, showing slower, buffered protein dynamics**.** (D, E, K, L) Simulated (left) and BurstLink-inferred (right) joint count distributions, without (D, K) and with (E, L) protein delay. (F, G, M, N) Rescaled conditional-probability heatmaps, without (F, M) and with (G, N) protein delay.

Comment 2: The scope of "gene-gene regulatory interactions" is narrow and does not account for enhancer-mediated regulation. The model defines gene-gene interactions exclusively as the sequence: mRNA to protein/TF to promoter binding to target gene activation (Eq. 13-14). This neglects enhancer-mediated regulation, which is a dominant mechanism for controlling transcriptional bursting. TF binding at distal enhancers (not just promoters) is a major determinant of burst frequency, as demonstrated by Bartman et al. (2016, Cell), Fukaya et al. (2016, Cell), Mermet, Yeung et al (Genes and Dev 2017) and importantly by Larsson et al. (2019, cited by the other), who showed genome-wide that enhancers primarily control burst frequency while core promoter elements control burst size. Trzaskoma et al. (2024, ref. 31) further demonstrated that factors acting beyond simple TF-promoter binding (Mediator, BRD4, cohesin) have distinct effects on burst frequency versus burst size, and that the mechanism of regulation matters more than its sign. While the scATAC-seq preprocessing identifies both promoter and enhancer regions (Fig. S4A), the mechanistic model itself only considers promoter-level regulation via a single Hill function. The authors should clarify this discrepancy and discuss whether enhancer-mediated effects are implicitly captured by the statistical model or fundamentally outside its scope. Additionally, it appears that the scATAC-seq data used for base-GRN construction comes from pre-built reference data (CellOracle datasets), not from matched multi-omic profiling of the same cells. Could the authors comment on the feasibility and potential benefit of using matched multi-ome data?

Response: We thank you for raising these important points regarding enhancer-mediated regulation and the use of scATAC-seq data. In the current revised manuscript, we clarified and commented on these two points in the Discussion section (Page 20, Line 566-571).

First, regarding enhancer-associated regulation, we fully agree that transcriptional bursting is not solely governed by promoter-proximal regulation. Enhancer-mediated TF binding is a major mechanism controlling burst frequency and transcriptional variability, as demonstrated by previous studies. We apologize for the ambiguity in our previous description, where the model formulation may have appeared to restrict gene regulation to promoter-level TF binding. In BurstLink, the inferred gene–gene regulatory interaction should be interpreted as a generalized regulatory effect from a TF

gene to a target gene, rather than as a promoter-only interaction. Mechanistically, such regulation may arise through TF binding at promoters, distal enhancers, or other regulatory elements that modulate the switching between transcriptionally inactive and active gene states. Accordingly, in the revised manuscript, we have modified the model description from "promoter-state switching" to a more general "gene-state switching". In this formulation, the Hill-function term is used as a coarse-grained representation of how the regulator affects the activation probability of the target gene, without assuming that this effect occurs exclusively at the promoter. We also agree that this coarse-grained treatment has limitations: the current BurstLink framework does not explicitly distinguish whether the inferred regulatory effect is mediated through promoter binding, enhancer binding. Therefore, enhancer-mediated effects are implicitly captured. We have now clarified this limitation in the Discussion and noted that extending BurstLink to separately model promoter- and enhancer-mediated regulatory effects will be an important direction for future work.

Regarding the use of scATAC-seq data, we agree that matched single-cell multiomics data would be ideal when available, because it can provide cell-state-matched chromatin accessibility and gene expression information. Such matched measurements could further improve the specificity of candidate TF–TG links by reducing potential confounding effects caused by differences in cell type composition, cell states, batch effects, or sample-to-sample variability between independently generated scRNA-seq and scATAC-seq datasets. We have commented on this point and discussed matched multiomics data as a useful future extension (Page 32, Line 992-999).

Comment 3: The Larsson et al. (2019) dataset was specifically designed to provide allele-resolved expression counts (CAST x C57), which is essential for accurately inferring burst kinetics from scRNA-seq data. Allele-specific counts avoid the confounding effect of summing two independently bursting alleles, each of which follows its own telegraph process. Larsson et al. performed inference independently on each allele and showed that kinetics inferred for the two alleles are correlated but represent two independent transcriptional processes. The authors of the present manuscript state (line 656-659) that they merge the two alleles "under the assumption that the expression distributions of almost all genes for the CAST and C57 alleles follow similar shapes." This merging doubles the effective cell count number but conflates two independent transcriptional processes, artificially inflating expression levels and altering the count distribution in ways that can bias burst parameter inference. The sum of two independent Poisson-Beta random variables does not itself follow a Poisson-Beta distribution, which is the distribution assumed by the model. The authors need to justify why they depart from this standard practice. Most importantly, Larsson et al. provide published allele-specific BF/BS values for the exact same cells. BurstLink should benchmark directly against these. Are the bursting kinetics inferred in their joint framework similar to those from methods that model bursting in an allele-specific manner? This comparison is missing from the manuscript. Also the authors should

discuss the consequences of sampling noises, as the theoretical Poisson-Beta is not invariant under bi-(multi-)nomial sampling.

Response: We thank you for this valuable problem regarding allele-specific preprocessing and burst kinetic inference. We apologize for the ambiguity in our previous description. We would like to clarify that our preprocessing did not sum the CAST and C57 allele-specific counts within each cell. Instead, we treated the two alleles as allele-level transcriptional observations and concatenated them at the observation level.

Specifically, there are two conceptually different ways to combine allele-specific data: (1) one is to concatenate CAST and C57 allele-specific observations, which increases the effective number of samples; (2) the other is to add the two allele-specific counts within each cell, which increases the observed expression level while keeping the number of cells unchanged. We agree with the reviewer that the latter approach would conflate two independently bursting transcriptional processes, alter the count distribution, and violate the assumption that a single gene follows a Poisson–Beta distribution, because the sum of two independent Poisson–Beta random variables is not necessarily Poisson–Beta distributed. However, this is not the strategy used in our analysis.

In our analysis, CAST and C57 allele-specific counts were concatenated as separate observations. The "pooling" step was performed under the assumption that the CAST and C57 allele-specific distributions are sufficiently identical for most genes included in downstream analysis, thereby increasing the effective sample size and improving the robustness of inference without directly inflating expression levels. To evaluate this assumption, we performed a distributional consistency test (KS test) between CAST and C57 allele-specific expression distributions. Among the genes retained for downstream analysis, 88.7% showed no significant difference between the two allele-specific distributions (Response Fig. 2.3A). We have added this analysis and clarified the allele-level pooling strategy in the revised Methods section (Page 31, Line 963-974).

We also benchmarked BurstLink-inferred burst kinetics against the published allele-specific burst frequency and burst size estimates from Larsson et al. Using the same dataset, we found that the burst kinetic parameters inferred by BurstLink correlate linearly with the published allele-resolved estimates (Response Fig. 2.3B-E). This comparison supports the validity of our inferred bursting kinetics, while BurstLink additionally provides gene–gene regulatory interaction information within the same framework.

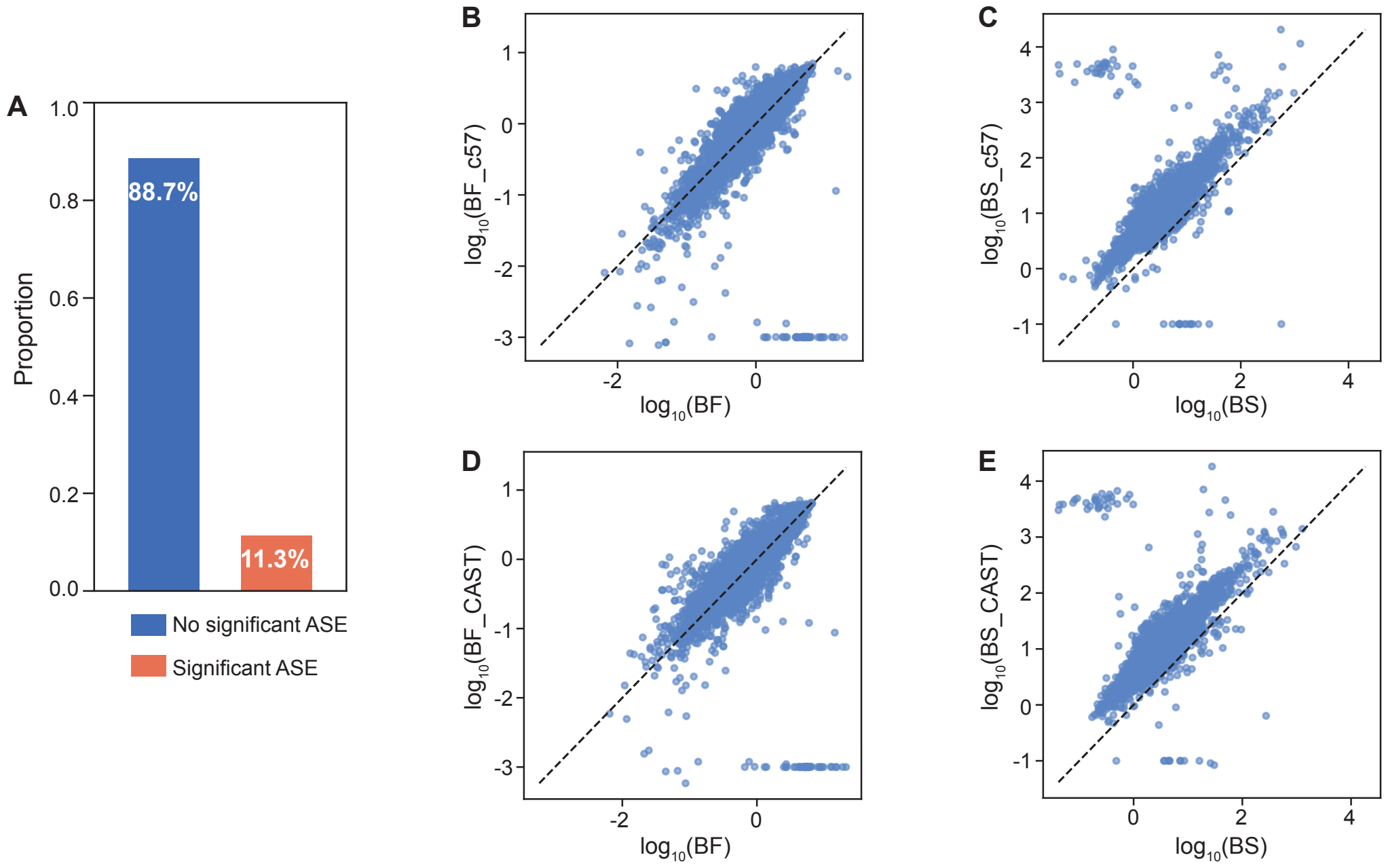

Response Fig. 2.3 | Evaluation of allele-specific expression distribution consistency and benchmarking of BurstLink-inferred bursting kinetics with Larsson et al. (A) Distributional consistency between CAST and C57 allele-specific expression (ASE) profiles assessed by the Kolmogorov–Smirnov (KS) test. (B-C) Comparison of BurstLink-inferred burst frequency (BF) and burst size (BS) at the gene level with the published allele-specific estimates reported by Larsson et al. for the CAST allele. (D-E) Comparison of BurstLink-inferred burst frequency (BF) and burst size (BS) at the gene level with the published allele-specific estimates reported by Larsson et al. for the C57 allele.

Regarding the influence of sampling noise on PoissonBeta-based burst kinetic inference. We have now added both theoretical clarification and systematic benchmarking analyses to address this concern. First, the bivariate Poisson-Beta model remains closed under independent binomial sampling. Specifically, let the true transcript counts (X_i, X_j) of any two genes be

$$X_i \mid \eta_i \sim \text{Poisson}(\phi_i \eta_i), X_j \mid \eta_j \sim \text{Poisson}(\phi_j \eta_j), \forall i, j \in \{1, 2, \ldots, n\}, \tag{2.1}$$

where (η_i, η_j) follow the joint beta-type latent distribution used in the bivariate Poisson–Beta model. Here, we set $(\eta_i, \eta_j) \sim f_{\text{Beta}}(\alpha_i, \beta_i, \alpha_j, \beta_j, \phi_i, \phi_j, w_{ij})$. If the observed counts (Y_i, Y_j) are generated by independent binomial sampling,

$$Y_i \mid X_i \sim \text{Binomial}(X_i, c_i), Y_j \mid X_j \sim \text{Binomial}(X_j, c_j), \tag{2.2}$$

by marginalizing over the unobserved true count X_i, we obtain

$$P(Y_i = y \mid \eta_i) = \sum_{x=y}^{\infty} P(Y_i = y \mid X_i = x) P(X_i = x \mid \eta_i) = \sum_{x=y}^{\infty} \binom{x}{y} c_i^y (1 - c_i)^{x-y} \frac{e^{-\phi_i \eta_i} (\phi_i \eta_i)^x}{x!}. \tag{2.3}$$

Let $m = x - y$, then

$$P(Y_i = y \mid \eta_i) = \frac{c_i^y (\phi_i \eta_i)^y}{y!} e^{-\phi_i \eta_i} \sum_{m=0}^{\infty} \frac{\left[(1-c_i)\phi_i \eta_i\right]^m}{m!} = \frac{(c_i \phi_i \eta_i)^y}{y!} e^{-\phi_i \eta_i} e^{(1-c_i)\phi_i \eta_i} = \frac{(c_i \phi_i \eta_i)^y}{y!} e^{-c_i \phi_i \eta_i}. \quad (2.4)$$

Thus,

$$Y_i \sim \text{Poisson}(c_i \phi_i \eta_i), Y_j \sim \text{Poisson}(c_j \phi_j \eta_j). \quad (2.5)$$

After integrating over the same joint latent distribution of (η_i, η_j), we obtain

$$(Y_i, Y_j) \sim \text{Bivariate Poisson-Beta}(\alpha_i, \beta_i, \alpha_j, \beta_j, c_i \phi_i, c_j \phi_j, w_{ij}). \quad (2.6)$$

Therefore, under independent binomial sampling with constant capture efficiencies, the bivariate Poisson–Beta family is closed. The Beta shape parameters and the dependency parameter are preserved, whereas the ϕ_i are rescaled to $c_i \phi_i$. We have added this theoretical derivation and clarification to the revised manuscript (Appendix Text S1.5).

Second, we performed a systematic benchmarking analysis to assess the robustness of BurstLink under sampling noise introduced during sequencing (Page 28, Line 856-857; Appendix Text S1.5; Appendix Fig. S16; Response Fig. 2.4). As illustrated in Response Fig. 2.4A, we modeled transcript capture as an independent binomial sampling process with capture efficiency c. We constructed a synthetic example to evaluate the practical impact of sampling noise. Specifically, we first generated the ground-truth expression distribution from the underlying transcriptional bursting model from SSA simulator (Response Fig. 2.4B) and then applied binomial downsampling to obtain the observed counts (Response Fig. 2.4C), thereby mimicking transcript loss during sequencing. BurstLink was subsequently applied to the downsampled observations. We evaluated the robustness of BurstLink at three complementary levels: (1) whether BurstLink recovers the underlying biological distribution from the observed noisy data; (2) whether the inferred parameters were sufficient to reconstruct the original true distribution; (3) whether the underlying burst kinetic parameters, including burst frequency (BF) and burst size (BS), are accurately estimated despite the presence of sampling noise. As shown in Response Fig. 2.4D-G, BurstLink recovered the underlying expression distributions despite substantial sampling noise, and the inferred burst kinetic parameters remained consistent with their corresponding ground-truth values. In addition, BurstLink demonstrates robustness to sampling noise over a range of capture efficiencies (Response Fig. 2.4H-J).

Furthermore, we performed sensitivity analysis using real scRNA-seq data. We applied binomial downsampling with different capture efficiencies and compared the burst parameters inferred from downsampled data with those inferred from the original data. As shown in Response Fig. 2.4K-N, burst frequency and burst size estimates remained highly correlations across different downsampling levels, indicating that BurstLink-derived bursting kinetics are stable under sampling perturbations.

We nevertheless agree with the reviewer that real scRNA-seq measurements are often more complex than the idealized binomial sampling model considered above. The impact of more complex measurement processes on burst kinetic inference remains an important open question. We have therefore expanded the Discussion section to explicitly discuss these limitations and highlight the

development of inference frameworks that more fully account for other technical effects as an important direction for future work (Page 20, Line 574-579).

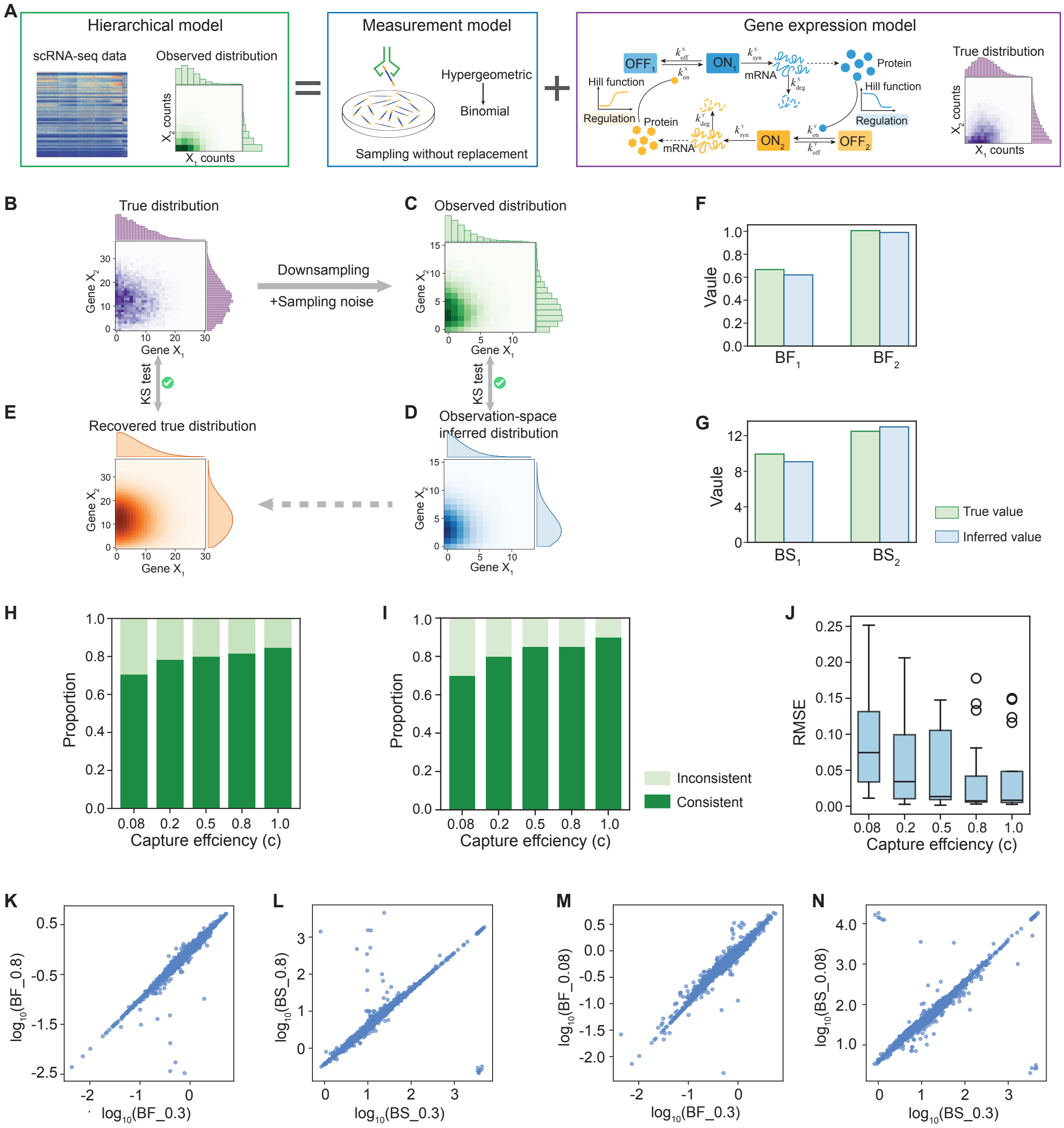

Response Fig. 2.4 | Systematic benchmarking of BurstLink robustness under capture efficiency variation. (A) Hierarchical model linking underlying gene expression model and measurement model. (B-G) Recovery of gene expression distributions and burst kinetic parameters from downsampled observations. (F-G) Accuracy of burst parameter inference across different capture efficiencies, quantified by the RMSE between inferred and true kinetic parameters. (H–J) Distributional consistency between recovered and true expression distributions across different capture efficiencies. (K–N) Sensitivity analysis using real scRNA-seq data.

Comment 4: A central claim is that BurstLink jointly infers regulatory interactions and bursting kinetics. However, the burst parameters are node-level variables that are initialized from single-gene

Poisson-Beta fits (Eq. 21-22), and the interaction parameter w_e is edge-specific. In the ADMM framework, the consensus variables are updated by averaging local edge estimates (Eq. 25-26). It is not clear how much the burst parameters actually change from their single-gene initializations after convergence. In other words, do the regulatory interactions meaningfully refine the burst kinetics estimates, or are BF and BS essentially the same as those obtained by existing single-gene tools (e.g., txburst from Larsson et al.)? The authors should (a) quantitatively compare their inferred BF/BS to those from single-gene methods on the same dataset, (b) show the magnitude of parameter shift during ADMM iterations, and (c) demonstrate specific cases where joint inference corrects or improves single-gene estimates. Without this, the added value of the joint framework over a two-step approach (infer bursting first, then infer interactions separately) remains unclear.

Response: We thank you for raising this important point. We agree that it is important to demonstrate whether the joint inference framework meaningfully refines burst kinetic estimates beyond single-gene Poisson-Beta fitting. In the revised manuscript, we have added new comparison analyses for parameters inference (Page 27-28, Line 838-845; Appendix Fig. S15; Response Fig. 2.5).

(a) First, to compare BurstLink with single-gene inference, we evaluated the inferred burst frequency (BF) and burst size (BS) using simulated datasets with known ground-truth parameters. Specifically, we generated datasets containing either non-interacting gene pairs or regulatory interactions and compared the root mean squared error (RMSE) of the recovered burst kinetic parameters against the true values. For non-interacting gene pairs, BurstLink and single-gene inference achieved comparable accuracy. In contrast, for gene pairs with regulatory interactions, BurstLink achieved significantly lower RMSE values than single-gene inference (Response Fig. 2.5A).

To further illustrate this result, we present two representative examples with positive and negative regulatory interactions, respectively (Response Fig. 2.5B–C). In both cases, the parameters inferred by BurstLink were consistently closer to the ground-truth values, while single-gene inference was able to approximately recover the true BF and BS values. These results demonstrate that explicitly modeling gene–gene regulatory interactions can improve burst kinetic estimation when regulatory coupling is present. We further investigate the origin of this improvement in section (c).

(b) Second, to assess the extent to which burst kinetic parameters are refined during ADMM optimization, we constructed a simulated three-gene regulatory network with known ground-truth parameters (Response Fig. 2.5D). We then tracked the optimization process starting from the single-gene initialization. As shown in Response Fig. 2.5E–F, both the ADMM residuals and the objective loss function converged rapidly, indicating stable optimization behavior. Importantly, the inferred burst kinetic parameters were not fixed at their initial values but were continuously updated throughout the optimization process. As shown in Response Fig. 2.5G–J, the estimated parameters progressively moved toward the ground-truth values and converged after several iterations. These results

demonstrate that the parameters are substantially refined during ADMM optimization, rather than simply inherited from the initial single-gene Poisson–Beta fits.

(c) Third, we investigated the origin of the improvement observed in the above section (a) and (b) using a two-gene regulatory system. As shown in Response Fig. 2.5H, single-gene inference accurately recovered the marginal distributions of the two genes but failed to reconstruct the underlying joint distribution, because it models each gene independently and does not account for regulatory coupling. In contrast, BurstLink can recover the joint distribution and passed the KS test against the simulated ground-truth distribution.

This difference is further reflected in the inferred conditional probability structure. Although the marginal distributions obtained by the two approaches were similar (Response Fig. 2.5I–J), single-gene inference could not capture the dependency between the two genes and therefore failed to recover the conditional relationship implied by the regulatory interaction. By contrast, BurstLink recovered the regulatory dependency structure through the joint model (Response Fig. 2.5K–L). As a result, BurstLink can not only provide more accurate estimates of burst kinetic parameters, but also infer gene–gene regulatory relationships that are inaccessible to models based solely on marginal distributions.

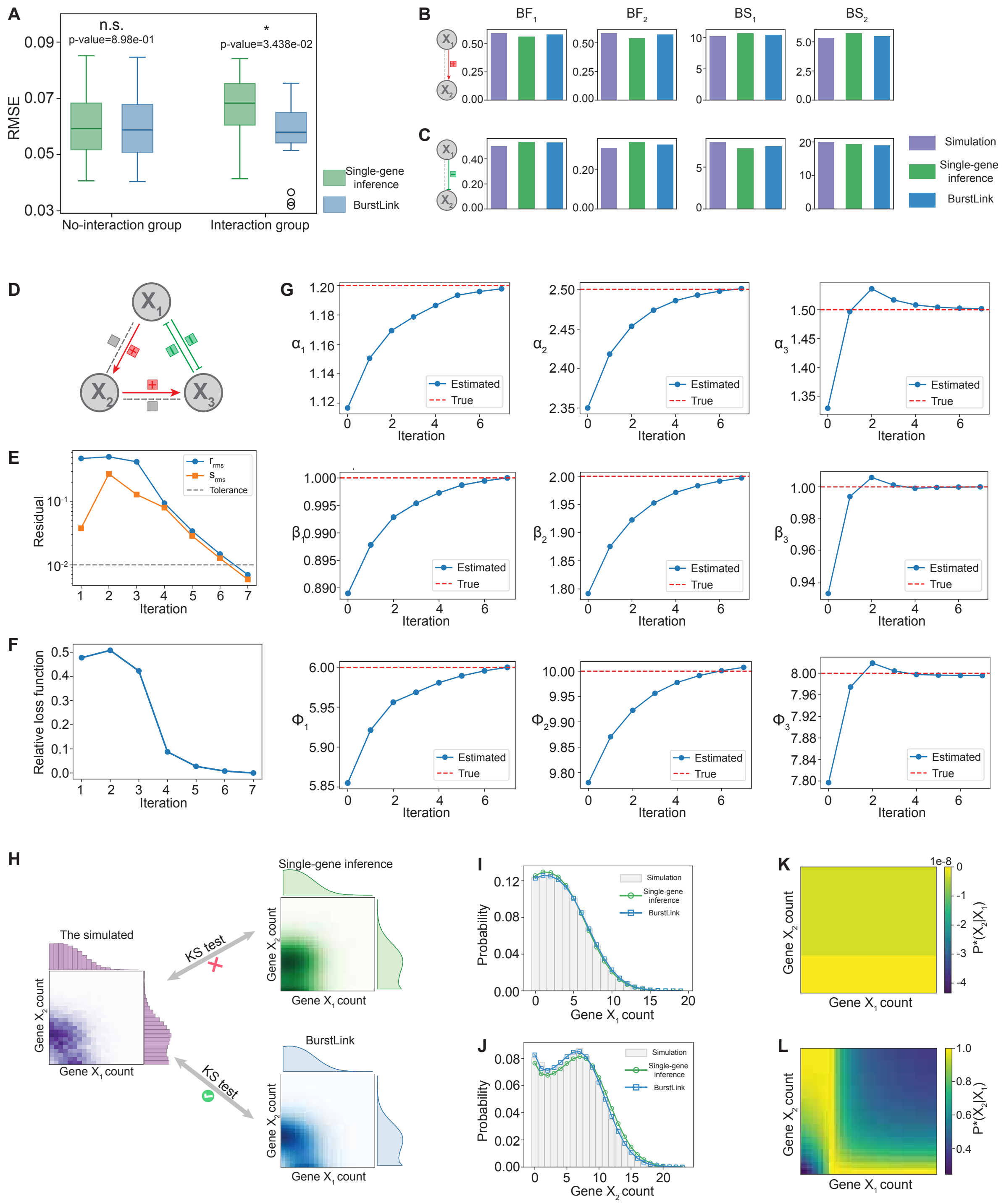

Response Fig. 2.5 | BurstLink refines burst kinetic estimates beyond single-gene fitting. (A) Comparison of burst kinetic parameter estimation errors between single-gene inference and BurstLink using simulated datasets with known ground-truth parameters. Gene pairs were divided into non-interacting and interacting groups. RMSE was calculated between inferred and true burst frequency (BF) and burst size (BS). (B–C) Two representative examples with positive and negative regulatory interactions, respectively. The inferred burst frequency (BF) and burst size (BS) obtained by single-gene inference and BurstLink are compared with the corresponding ground-truth values. (D) Simulated three-gene regulatory network used to evaluate parameter refinement during ADMM optimization. (E)

Evolution of ADMM primal residual and dual residual during optimization. (F) Evolution of the objective loss function during ADMM optimization. (G) Iterative trajectories of parameters. (H) Comparison of reconstructed joint gene expression distributions for a two-gene regulatory system. The simulated ground-truth distribution is shown on the left. Single-gene inference reconstructs marginal distributions but fails to recover the joint dependency structure and does not pass the Kolmogorov–Smirnov (KS) test. In contrast, BurstLink recovers the joint distribution and passes the KS test. (I–J) Marginal expression distributions of the two genes from single-gene inference and BurstLink, respectively. (K–L) Conditional probability landscapes inferred from single-gene inference and BurstLink, respectively.

Comment 5: Despite inferring interactions and bursting jointly, the downstream analyses (Section 2.5, Fig. 5) rely entirely on post-hoc statistical tests (t-tests, Bayesian ridge regression) to establish the relationship between regulatory interactions and burst kinetics. If the model truly captures the coupled dynamics, one would expect the parameter we and the burst parameters to be mechanistically linked within the model itself. Instead, the link between regulation type/strength and BF/BS/CV2 is established only through external statistical analysis of the inferred parameters. This raises a fundamental question: what is the gain of doing both together in a joint model if the relationship between the two must still be discovered through separate post-hoc analyses? It is worth noting that the directionality captured by RMI is also a post-hoc construction from the conditional probabilities, not something emerging from the joint model, since the coupling parameter w is itself symmetric. The authors should articulate more clearly what the joint inference provides that a sequential approach would not.

Response: We thank you for raising this important conceptual question. We agree that the analyses in Section 2.5 are downstream statistical analyses of the parameters inferred by BurstLink. However, these analyses were performed at the genome-wide level, because the biological question addressed in this section is not limited to a single transcription factor–target gene pair. To further clarify this workflow, we have added a schematic overview of the entire analysis pipeline in Response Fig. 2.7A.

The framework consists of two conceptually distinct steps. First, BurstLink jointly infers transcriptional bursting parameters and regulatory interactions for individual gene pairs within a unified statistical mechanistic model. Second, the inferred parameters are analyzed using statistical approaches to identify global relationships between regulatory interactions and bursting kinetics at the genome-wide scale. Similar strategies have been used in previous studies, where joint inference provides interpretable kinetic parameters, and downstream genome-wide analyses are then performed. For example, Sukys and Grima modeled transcriptional bursting together with cell-cycle-dependent effects and then performed genome-wide analyses of the inferred burst parameters to reveal systematic

principles of burst frequency and burst size regulation across cell-cycle phases [Sukys et al., Nucleic Acids Research, 2025].

The gain of joint inference is not that the genome-wide relationship between regulation and bursting is automatically encoded as a fixed formula in the model. Rather, the main advantage of BurstLink is that regulatory dependency and transcriptional bursting dynamics are estimated in a coupled and self-consistent manner within the same joint distributional framework. Without jointly modeling these two components, separate inference may bias parameter estimation, because regulatory interactions and burst kinetics can influence each other and together determine the observed joint expression distribution of each gene pair (see comments 2.4). Specifically, single-gene bursting inference may absorb regulatory effects into intrinsic burst parameters, whereas regulatory interaction inference based only on correlation or mutual information may be confounded by bursting-driven variability. We have now explicitly added statement in the revised manuscript what the joint inference provides that a sequential approach would not (Page 28, Line 843-846).

We agree that RMI is computed after the model is fitted, and that the coupling parameter w is symmetric. However, the directional, asymmetric quantity does not originate from raw counts or from an external association measure: as illustrated in Response Fig. 2.7B, it is derived analytically from the BurstLink-inferred joint distribution, through the model's conditional probability functions and the resulting regulation response curves. Its asymmetry arises from the gene-specific bursting kinetics embedded in the fitted joint distribution and is therefore a consequence of the joint model — expressed through its conditional structure rather than through w directly.

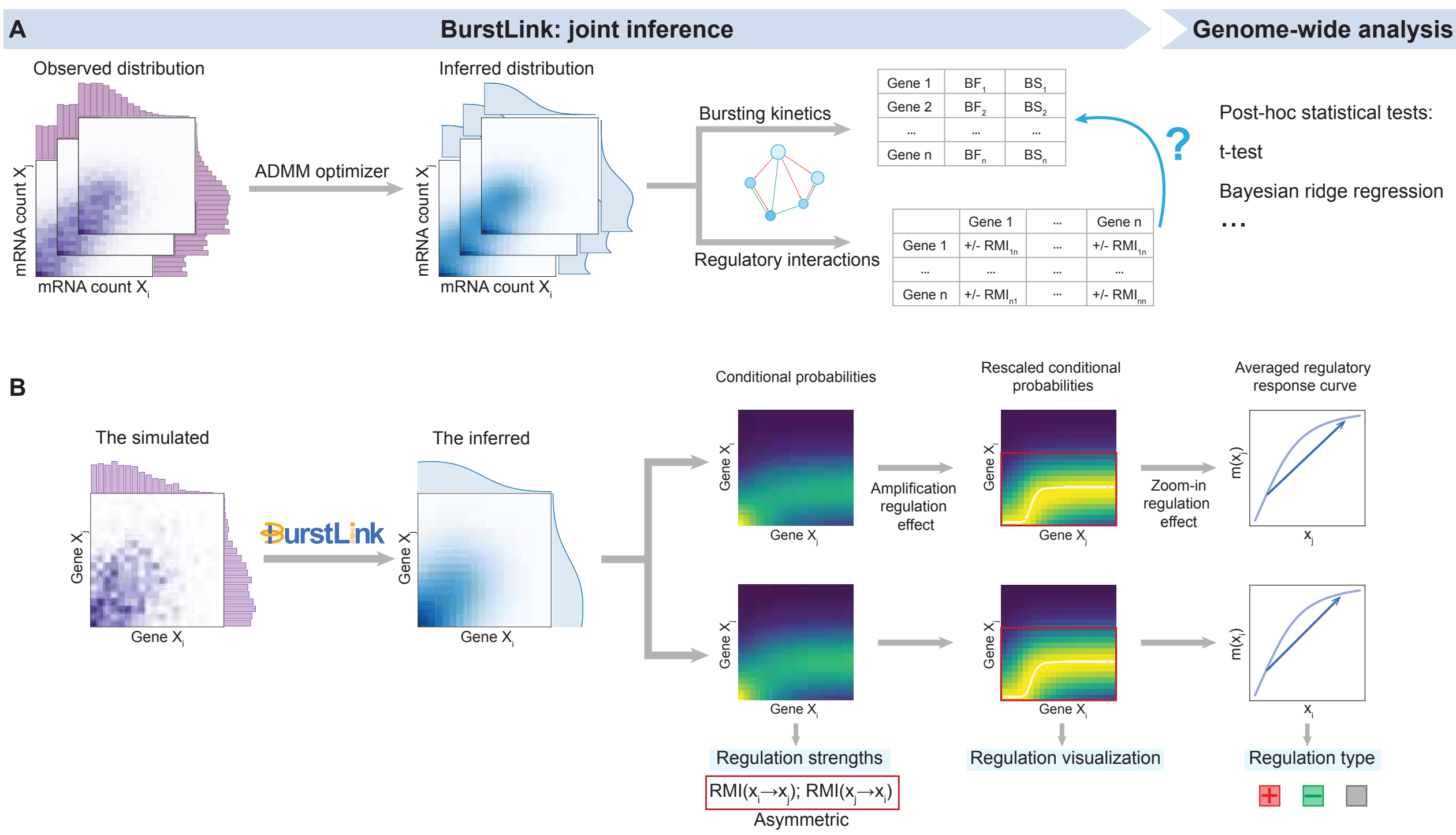

Response Fig. 2.6 | Overview of the BurstLink framework. (A) Schematic illustration of the overall analysis pipeline. BurstLink first jointly infers transcriptional bursting parameters and gene regulatory

interactions from observed single-cell gene expression distributions using an ADMM-based optimization framework. The inferred bursting and regulatory parameters are then used for downstream genome-wide analyses, including statistical modeling and hypothesis testing, to identify global relationships between transcriptional bursting dynamics and regulatory interactions. (B) Schematic illustration of the regulatory interaction inference procedure. Starting from the BurstLink-inferred joint probability distribution, conditional probability functions are constructed and subsequently transformed into regulation response curves, from which the regulation type and RMI scores are obtained to quantify regulatory directionality and strength.

Comment 6: The entire inference is performed on mature (spliced) mRNA counts. Unspliced/spliced read ratios carry substantial information about transcriptional dynamics and are now routinely extracted from scRNA-seq data (La Manno et al., 2018; Bergen et al., 2020). Recent methods for burst kinetics inference (e.g., biVI, Carilli et al., 2024, cited by the author; Gorin et al., 2022) leverage both spliced and unspliced counts to improve parameter identifiability. While the authors mention RNA splicing kinetics as a future direction (line 381), this is a significant limitation given the current state of the field. At minimum, the authors should discuss whether incorporating unspliced counts could improve the identifiability of their model parameters, particularly for separating transcription rate from degradation rate.

Response: We thank you for mentioning this important point. We agree that the current BurstLink framework does not explicitly incorporate unspliced and spliced mRNA information, and that this represents an important limitation. In the revised manuscript, we have added a discussion of this limitation, cited relevant studies that leverage unspliced/spliced counts for transcriptional dynamics and burst kinetics inference, and discussed both the potential benefits and challenges of incorporating such information into our framework (Page 20, Line 561-565).

Specifically, the current model infers effective steady-state bursting parameters from mature mRNA count distributions. Without explicitly modeling unspliced counts or RNA processing/degradation kinetics, the absolute separation between transcription rate, splicing rate, and degradation rate is not fully identifiable. Incorporating joint unspliced/spliced count modeling in future extensions could provide additional constraints on RNA production, processing, and degradation, thereby improving parameter identifiability and enabling a more detailed decomposition of burst kinetics. At the same time, such an extension would require a more complex stochastic model. For example, for each TF–target gene pair, one may need to model the joint distribution of unspliced and spliced counts for both genes, leading to a four-dimensional count distribution rather than the current two-dimensional mature mRNA count distribution. This would substantially increase computational complexity and would also require careful treatment of technical noise, sparsity, and modality-specific biases in unspliced count measurements. We have now clarified these points in the Discussion.

Comment 7: Lines 111-114 state that the input is scRNA-seq data, with scATAC-seq used for "pre-filtering." The manuscript should be more transparent about whether BurstLink is practically useful with scRNA-seq data alone or whether scATAC-seq is effectively required. Additionally, does BurstLink require UMI counts from plate-based protocols (Smart-seq2, as used in the paper), or can it work with standard 10x Genomics data (higher dropout)? The data requirements and technology compatibility should be stated upfront.

Response: We thank you for raising this important point. In the revised manuscript. We have further clarified the data requirements, technology compatibility, and the optional role of scATAC-seq in the Methods section of the revised manuscript to avoid ambiguity for readers (Page 31, Line 951-952).

First, BurstLink can be trained and applied entirely using scRNA-seq data alone. The core inference framework relies on transcriptional snapshot measurements from scRNA-seq count distributions to jointly infer transcriptional bursting parameters and regulatory interaction information between genes. In other words, neither scATAC-seq data nor additional multi-omics measurements are strictly required for the inference procedure itself. The mechanistic model and likelihood-based inference are fundamentally driven by scRNA-seq count distributions.

The role of scATAC-seq in our framework is optional and serves only as a pre-filtering or structural prior step. Specifically, for large-scale GRN inference, considering all possible pairwise gene interactions leads to a combinatorially large search space and substantial computational cost. When available, scATAC-seq data can be used to prioritize candidate TF-target gene pairs and remove many biologically implausible regulatory edges before model fitting, thereby reducing the computational burden. Importantly, scATAC-seq is not used to estimate bursting kinetics or regulatory strengths directly, but only to constrain the candidate interaction topology. Therefore, BurstLink remains fully applicable in settings where only scRNA-seq data are available.

Second, BurstLink is compatible with scRNA-seq data generated from different sequencing technologies. In the current manuscript, we demonstrate this compatibility using both Smart-seq2 and 10x Genomics datasets. Specifically, the analyses in Results 4 and 5 are based on Smart-seq2 data, whereas the analysis in Result 6 uses 10x Genomics data. Although different technologies exhibit different sparsity and dropout characteristics, BurstLink operates on snapshot count distributions and can therefore be applied broadly across commonly used single-cell RNA-seq platforms.

Comment 8: To support that the model can infer biologically relevant regulatory interactions, the authors should dissect the network inferred in Fig. 4E and validate it through a thorough analysis of published literature in the relevant cell types.

Response: Thank you for this helpful suggestion. We agree that a biological interpretation and literature-based validation of the inferred network are important for supporting the ability of BurstLink to recover biologically relevant regulatory interactions.

We selected a biologically interpretable regulatory subnetwork (Fig. 4E) consisting of 12 genes from the mouse embryonic fibroblast dataset. Collectively, these genes define a coordinated fibroblast stress-response regulatory network that integrates immediate-early activation, inflammatory amplification, proliferative remodeling, and oxidative-stress adaptation. A schematic representation of these functional modules and their cross-regulatory relationships is shown in Response Fig. 2.7A. The BurstLink-inferred regulatory subnetwork is shown in Response Fig. 2.7B, where node color and size indicate bursting-related features and edges represent inferred regulatory interactions.

We further performed a literature-based validation of the inferred regulatory interactions. For each directed gene pair in the subnetwork, we curated published evidence and assigned a regulatory label indicating positive regulation, negative regulation, no reported regulation, or insufficient literature evidence. We then compared these literature-supported annotations with the regulatory interactions inferred by BurstLink. First, we evaluated whether BurstLink could correctly identify the existence of regulatory interactions, regardless of whether the interaction was activating or repressive. This binary edge-detection analysis yielded an AUROC value 0.74 (Response Fig. 2.7C), indicating that the inferred network is significantly enriched for literature-supported regulatory relationships.

Second, for interactions with literature-supported regulatory signs, we evaluated whether BurstLink could correctly predict the direction of regulation. BurstLink achieved a regulatory sign prediction Macro-F1 score of approximately 0.81 (Response Fig. 2.7D), demonstrating substantial agreement with experimentally reported regulatory effects. Notably, none of the curated negative regulatory interactions were misclassified as positive interactions. This result suggests that BurstLink is capable of capturing biologically meaningful antagonistic regulatory relationships in addition to recovering regulatory connectivity. The complete literature-based validation table, including curated regulatory signs, supporting PMIDs, and performance evaluation results, has been added to the Supplementary Information in the revised manuscript (Appendix Fig. S6; Appendix Table S3; Response Table S1).

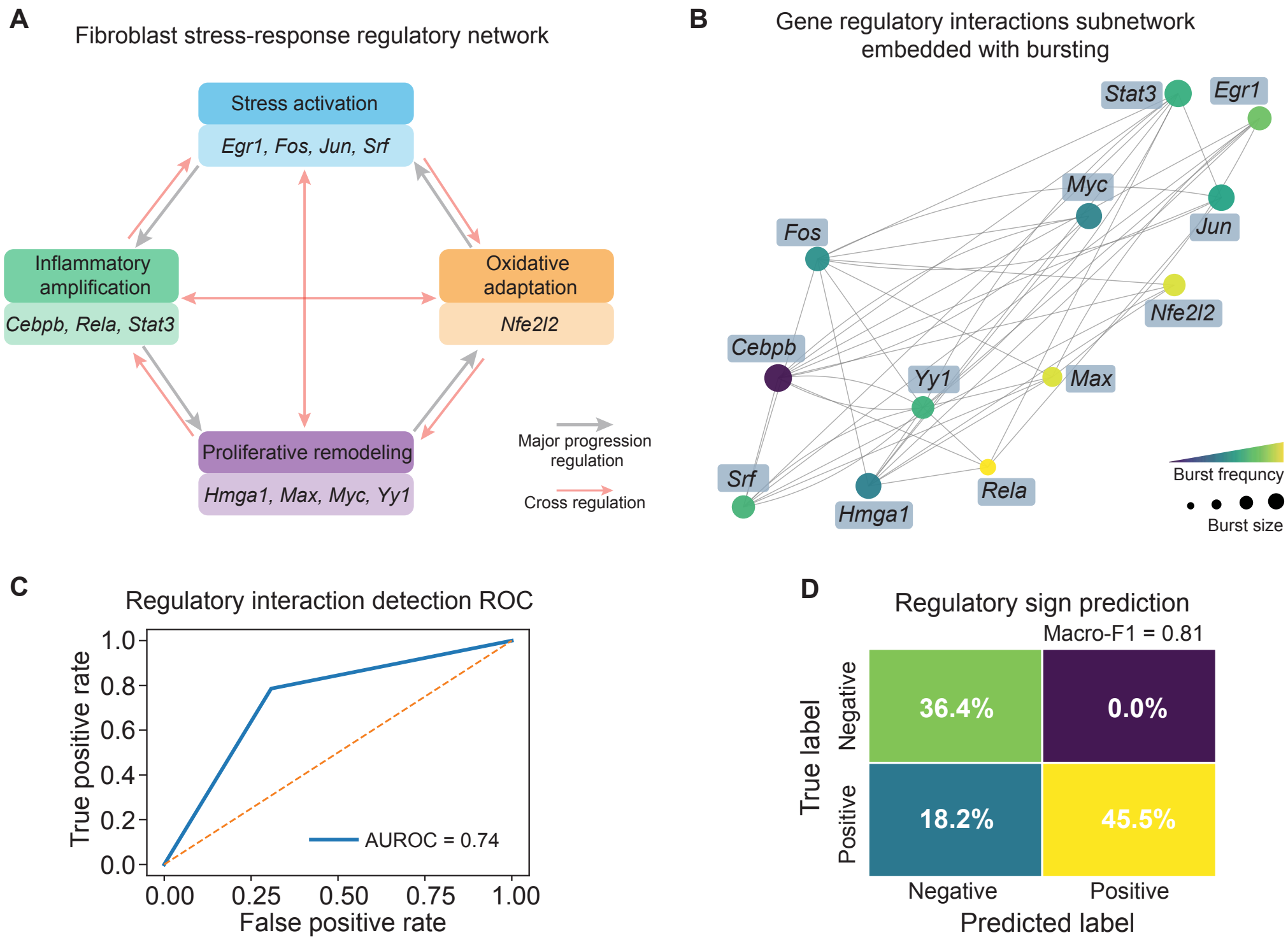

Response Fig. 2.7 | Literature-supported validation of a fibroblast stress-response regulatory network inferred by BurstLink. (A) Conceptual schematic of a fibroblast stress-response regulatory network constructed from 12 genes identified from mouse embryonic fibroblast (MEF) scRNA-seq data. These regulators were grouped into four interconnected transcriptional programs: stress activation (*Egr1*, *Fos*, *Jun*, and *Srf*), inflammatory amplification (*Cebpb*, *Rela*, and *Stat3*), proliferative remodeling (*Hmga1*, *Max*, *Myc*, and *Yy1*), and oxidative adaptation (*Nfe2l2*). Gray arrows indicate major progression relationships among functional states, whereas red arrows denote cross-regulatory interactions between modules. (B) BurstLink-inferred regulatory subnetwork for the selected regulators. Node size represents log-transformed burst size, node color represents log-transformed burst frequency, and edges denote inferred regulatory interactions. (C) Literature-based validation of regulatory interaction detection. Receiver operating characteristic (ROC) analysis demonstrates the ability of BurstLink to recover literature-supported regulatory interactions, achieving an AUROC of 0.74. (D) Validation of inferred regulatory signs using literature-supported activating and repressive interactions. The normalized confusion matrix shows the agreement between curated regulatory signs and BurstLink predictions, with a macro-F1 score of 0.81.

Response Table S1. Literature-supported gene regulatory interactions used for biological validation of the BurstLink-inferred subnetwork

Gene name1	Gene name2	Regulation type1	Regulation type2	PMID
Cebpb	*Fos*	Positive	Positive	11151091
Cebpb	*Hmga1*	Positive	Positive	12665574
Cebpb	*Jun*	Regulation	Regulation	18026136

Cebpb	*Myc*	Positive	Regulation	20581311
Cebpb	*Rela*	Regulation	Regulation	12667216
Cebpb	*Srf*	Positive	Positive	11151091
Cebpb	*Stat3*	Regulation	Regulation	20581311
Cebpb	*Yy1*	Regulation	Regulation	8892889
Egr1	*Fos*	Regulation	Regulation	1909429
Egr1	*Jun*	——	Positive	18281687
Egr1	*Max*	Negative	——	19032775
Egr1	*Myc*	Regulation	Regulation	25147211
Egr1	*Srf*	——	Positive	2040600
Egr1	*Stat3*	——	Positive	25012664
Fos	*Hmga1*	Positive	——	27882471
Fos	*Jun*	Regulation	Regulation	1904542, 3141060
Fos	*Myc*	Regulation	Regulation	21135252
Fos	*Rela*	Regulation	Regulation	12667216
Fos	*Srf*	——	Positive	2453393, 3071491
Fos	*Stat3*	——	Positive	12600988
Fos	*Yy1*	——	Negative	7769693, 8276234
Hmga1	*Jun*	Positive	——	26980699
Hmga1	*Myc*	Positive	——	23184673
Hmga1	*Rela*	Regulation	——	27025651
Hmga1	*Stat3*	Regulation	Regulation	19074878
Jun	*Myc*	Positive	——	21135252
Jun	*Rela*	Regulation	Regulation	12667216
Jun	*Srf*	Regulation	Regulation	10318842
Jun	*Stat3*	Regulation	Regulation	20516108
Jun	*Yy1*	——	Regulation	7935475
Max	*Myc*	Positive	Positive	1501888, 8425218
Myc	*Nfe2l2*	Positive	——	33782124
Myc	*Rela*	——	Positive	8289784, 1114727
Myc	*Stat3*	——	Positive	9874564, 20581311
Myc	*Yy1*	Negative	Negative	9696045, 10858544
Nfe2l2	*Rela*	Negative	Negative	18241676
Nfe2l2	*Stat3*	Regulation	Regulation	32336239
Rela	*Stat3*	Regulation	Regulation	22466650
Rela	*Yy1*	Positive	——	17466695
Srf	*Yy1*	——	Positive	8276234
Stat3	*Yy1*	Positive	Negative	22190092, 33300076

Note: *Positive* indicates that published studies support a positive regulatory effect (activation). *Negative* indicates that published studies support a negative regulatory effect (repression or inhibition).

Regulation indicates that published evidence supports the existence of a regulatory relationship between the two genes, but the direction or sign of regulation remains unclear. — indicates that no direct experimental evidence was identified supporting a regulatory relationship between the two genes.

Minor Comments

Comment 9: Fig. 3C panels c1, c3, c4 are not cited in the main text. Multiple supplementary figure panels (e.g., Fig. S5D-E) are referenced inconsistently or not at all. Figures contain an excessive number of panels that are difficult to parse. For instance, Fig. 4 has 14 panels (A-N), with multiple plots per panel, many of which could be moved to the supplement. The double-layer network visualization (Fig. 4J) is particularly unclear: what biological insight can the reader extract from this plot? Consider simplifying the main figures to highlight the key messages and moving supporting panels to the supplement.

Response: We thank you for this detailed suggestion. In the revised manuscript, we have carefully checked all figure citations and corrected the inconsistent or missing references to figure panels in the main text and supplementary materials. We also simplified the main figures to improve readability. In particular, we simplified Fig. 4 to focus on the key results, and moved several supporting panels to the Supplementary Figures (Page 12, Line 346, Fig. 4; Appendix Fig. S5).

Comment 10: The manuscript uses numerous acronyms (BF, BS, RMI, CMI, NMI, CV2, GRN, TF, TG, SSA, KS, ROC, AUC, ADMM, etc.) that are often not spelled out in figure legends. Since readers frequently examine figures before reading the full text, all acronyms should be defined upon first use in each figure caption. This is especially important for domain-specific terms like RMI and CMI that appear in Fig. 4F-G without definition in the caption.

Response: We thank you for this helpful suggestion. In the revised manuscript, we have carefully checked all figure legends and defined acronyms upon their first use in each caption.

Comment 11: Line 406: "the any two binary variables" should read "any two variables."

Response: Thank you for pointing this out. We have corrected the phrase from “the any two binary variables” to “any two variables” in the revised manuscript (Page 21, Line 606).

Comment 12: In the dynamic mechanistic model (Eq. 13), the degradation rates kdeg are set to 1 throughout in Table S1 and vary (0.8, 1.2, 1.5) in Table S2. Both tables lack descriptive captions, and it is unclear what the different rows represent. The authors should clarify what timescale normalization is implied. Since all rates in the telegraph model are expressed relative to the degradation rate, the absolute values of mRNA and protein half-lives matter for interpreting the biological meaning of the inferred parameters. This should be discussed.

Response: We thank you for pointing this out, and we apologize for missing descriptive captions for these tables. In the revised manuscript, we have added detailed captions to Tables S1 and S2 to clarify what each row represents and how the parameter settings were used in the simulations.

We have also clarified the timescale normalization used in the dynamic mechanistic model. For the simulation in Fig.2B and Fig. S2, the mRNA degradation rate was set to $k_{\text{deg}} = 1$. For the simulations shown in Fig. 2C, parameter configurations corresponding to different relative mRNA degradation-rate settings are listed in Table S2. In the inference procedure, all kinetic parameters were similarly normalized by the mRNA degradation rate and should therefore be interpreted as relative, dimensionless quantities. We have now stated this explicitly in the Methods and discussed that assigning absolute biological timescales would require external information about mRNA and protein half-lives (Page 19, Line 537-544).

Comment 13: The average capture efficiency is set to 0.3 for Smart-seq2 (line 583). How sensitive are the results to this value? This should be tested, especially since the method claims applicability to different scRNA-seq technologies, which have widely varying capture efficiencies. This is shortly tested in the simulation (Fig S2) but barely described; one sentence for 12 panels. How does the binomial sampling change the Poisson-Beta model? (See above).

Response: Thank you for this question. As discussed above, we have added sensitivity analysis regarding the capture efficiency (Comment 2.3, Response Fig. 2.4; Appendix Fig. S16), and more description for original Fig. S2 (now in Appendix Fig. S19) (Page 30, Line 928-936). We have also included a theoretical derivation and discussion of the invariance of the Poisson-Beta model under binomial sampling (Comment 2.3).

Comment 14: The manuscript emphasizes scalability but does not report runtime or memory usage. How long does inference take for the 4311 genes and 291'228 gene pairs? How does this scale with gene number and cell number?

Response: We thank you for raising this important point. We agree that computational scalability is an important consideration for practical applications of BurstLink (Appendix Text S1.7; Appendix Fig. S21).

For the full mouse embryonic fibroblast (MEF) dataset containing 4,311 genes and 291,228 candidate gene pairs, the complete BurstLink analysis required approximately 14.2 h using 8 CPU cores and was reduced to approximately 7.8 h on a 16-core workstation. These results demonstrate the favorable parallel scalability of BurstLink and indicate that computational efficiency can be substantially improved with additional computing resources.

For the gene-number benchmark, we randomly sampled subnetworks containing 20, 50, 100, 500, and 1,000 genes from the MEF dataset. For each network size, the sampling and inference procedures

were repeated 50 times, and the runtime distributions were recorded (Response Fig. 2.8A). For the cell-number benchmark we generated synthetic datasets using stochastic simulation algorithm (SSA) simulations and randomly sampled 500, 1,000, 2,000, and 5,000 cells from the simulated data. For each cell number, the sampling and inference procedures were repeated 50 times, and the runtime distributions were recorded (Response Fig. 2.8B). Together, BurstLink remained computationally feasible for large subnetworks, demonstrating favorable scalability with respect to network size. Runtime is substantially more sensitive to network size than to cell number, indicating that the number of candidate gene pairs is the primary determinant of computational cost.

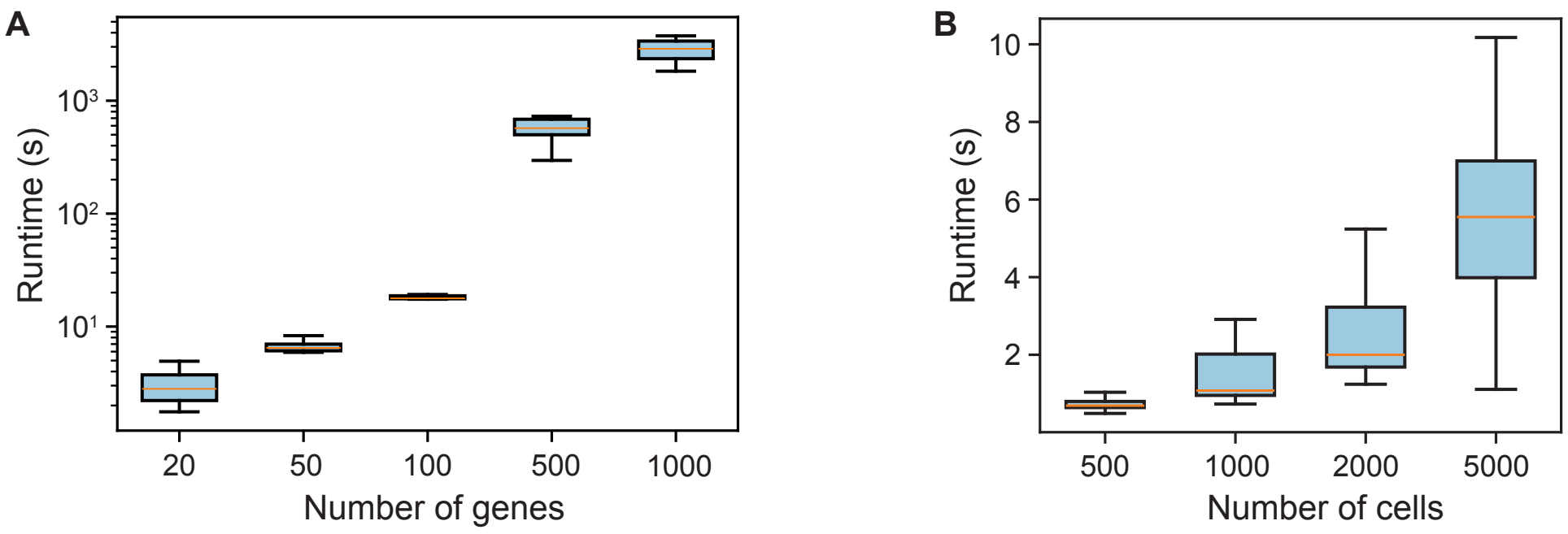

Response Fig. 2.8 | Computational scalability of BurstLink. (A) Boxplots of runtime distributions for subnetworks containing different numbers of genes. (B) Boxplots of runtime distributions for datasets containing different numbers of cells.

3rd Jul 2026

Manuscript Number: MSB-2026-13561R
Title: Deciphering global transcriptional dynamics coordinated by gene-gene regulatory interactions using single-cell data
Author: Liying Zhou
Songhao Luo
Zhiwei Huang
Zhenquan Zhang
Zihao Wang
Jiajun Zhang

Dear Prof Zhang,

Thank you for sending us your revised manuscript. We have now heard back from the original Reviewer #3 who was asked to re-evaluate your study. As you will see, the reviewer is now satisfied with the modifications made. Before we can formally accept your manuscript, we would ask you to address the following editorial-level issues:

1. Please remove the "Author contributions" section from the manuscript file.

2. Please add a "Disclosure and competing interests statement" section.

3. Please remove the figures from the manuscript file. The figure legends should be placed after the "References" section.

4. Please ensure that all figure and table callouts appear in sequential order. Also, add the missing callouts for Appendix Figure S21 and Appendix Table S1.

5. Appendix: Please submit the Appendix as a PDF file. The title page should contain "Appendix for [Manuscript Title]" (the author and affiliation list is not required). The title page should also include a Table of Contents with page numbers for all listed items.

Please use the nomenclature Appendix Figure Sx and Appendix Table Sx consistently throughout both the manuscript and the Appendix PDF. In addition, please remove all highlighted text, as the Appendix will not be proofread.

6. Please remove the "Public Data" section. Instead, please cite all publicly available datasets that were reused by including data citations in the reference list.

Data citations in the article text are also distinct from normal bibliographical citations and should directly link to the database records from which the data can be accessed. In the main text, data citations are formatted as follows: "Data ref: Smith et al, 2001".

In the Reference list, data citations must be labeled with "[DATASET]". A data reference must provide the database name, accession number/identifiers and a resolvable link to the landing page from which the data can be accessed at the end of the reference.

Please carefully review the further instructions are available at < https://link.springer.com/journal/44320/submission-guidelines#cms-Reference-guidelines > and ensure that all data citations comply with our journal's requirements.

7. Please address the following issues in figure legends:
- Please define the annotated p values ****/***/**/* as well as provide the exact p-values for the same in the legend of figure 4D, I, K; 5F-H as appropriate.
- Please indicate the statistical test used for data analysis in the legends of figures 4D,I-K; 5F-H
- Please note that the box plots need to be defined in terms of minima, maxima, centre, bounds of box and whiskers, and percentile in the legends of figures 4C, D; 5C, D, F, G, H
- Please note that information related to n is missing in thelegends of figures 4C, D; 5B, C, D, F, G, H
- Please note that the error bars are not defined in the legends of figures 5B, C, D, F, G, H.

8. Sections need to be named and the order should be corrected: Title page - Abstract - Introduction - Results - Discussion - Methods - Data Availability - Acknowledgements - Disclosure and Competing Interests Statement - References - Figure Legends - Table(s) - Expanded View Figure Legends.

Use the following link to submit your revised paper:

https://msb.msubmit.net/cgi-bin/main.plex

As a matter of course, please make sure that you have correctly followed the instructions for authors as given on the submission website.

Thank you for submitting this paper to Molecular Systems Biology.

Kind regards,
Jingyi

Jingyi Hou, PhD
Senior Editor
Molecular Systems Biology

*** PLEASE NOTE *** As part of the EMBO Press transparent editorial process initiative (see our Editorial at https://dx.doi.org/10.1038/msb.2010.72 , Molecular Systems Biology will publish online a Review Process File to accompany accepted manuscripts. When preparing your letter of response, please be aware that in the event of acceptance, your cover letter/point-by-point document will be included as part of this File, which will be available to the scientific community. More information about this initiative is available in our Instructions to Authors. If you have any questions about this initiative, please contact the editorial office (msb@embo.org).

Reviewer #3:

We are satisfied with the extensive revision and highly detailed replies to our questions.

Response to Editor

Thank you for your positive decision and the detailed guidance on the final amendments. We have carefully addressed all the comments point by point. Below is our detailed response.

1. Please remove the "Author contributions" section from the manuscript file.

Response: We have removed the "Author contributions" section entirely from the manuscript as requested. The author contributions have already been provided in the submission system.

2. Please add a "Disclosure and competing interests statement" section.

Response: We have added the "Disclosure and competing interests statement" section as requested. The updated section appears on Line 980 of the manuscript. We have reviewed the journal's updated competing interests policy (January 2022) and confirm that our disclosure remains accurate and complete; no additional competing interests need to be declared.

3. Please remove the figures from the manuscript file. The figure legends should be placed after the "References" section.

Response: We have removed all figures from the manuscript file and placed the Figure Legends after the References section, as requested.

4. Please ensure that all figure and table callouts appear in sequential order. Also, add the missing callouts for Appendix Figure S21 and Appendix Table S1.

Response: We have checked all figure and table callouts to ensure that they appear in sequential order and have added the missing callouts for Appendix Figure S21 (Page 24, Line 856-859) and Appendix Table S1 (Page 5, Line 184).

5. Appendix: Please submit the Appendix as a PDF file. The title page should contain "Appendix for [Manuscript Title]" (the author and affiliation list is not required). The title page should also include a Table of Contents with page numbers for all listed items. Please use the nomenclature Appendix Figure Sx and Appendix Table Sx consistently throughout both the manuscript and the Appendix PDF. In addition, please remove all highlighted text, as the Appendix will not be proofread.

Response: We have revised the Appendix accordingly. Specifically, we have: (1) submitted the Appendix as a single PDF file; (2) added a title page with the title "Appendix for Deciphering global transcriptional dynamics coordinated by gene-gene regulatory interactions using single-cell data"; (3) included a Table of Contents on the title page with page numbers for all listed items; (4) used the nomenclature Appendix Figure Sx and Appendix Table Sx consistently throughout both the manuscript and the Appendix PDF; and (5) removed all highlighted text from the Appendix.

6. Please remove the "Public Data" section. Instead, please cite all publicly available datasets that were reused by including data citations in the reference list. Data citations in the article text are also distinct from normal bibliographical citations and should directly link to the database records from which the

data can be accessed. In the main text, data citations are formatted as follows: "Data ref: Smith et al, 2001". In the Reference list, data citations must be labeled with "[DATASET]". A data reference must provide the database name, accession number/identifiers and a resolvable link to the landing page from which the data can be accessed at the end of the reference. Please carefully review the further instructions are available at <https://link.springer.com/journal/44320/submission-guidelines#cms-Reference-guidelines> and ensure that all data citations comply with our journal's requirements.

Response: We have removed the "Public Data" section and revised all data citations in both the main text and the reference list to comply with the journal's requirements.

7. Please address the following issues in figure legends:

- Please define the annotated p values ****/***/**/* as well as provide the exact p-values for the same in the legend of figure 4D, I, K; 5F-H as appropriate.
- Please indicate the statistical test used for data analysis in the legends of figures 4D, I-K; 5F-H
- Please note that the box plots need to be defined in terms of minima, maxima, centre, bounds of box and whiskers, and percentile in the legends of figures 4C, D; 5C, D, F, G, H
- Please note that information related to n is missing in the legends of figures 4C, D; 5B, C, D, F, G, H
- Please note that the error bars are not defined in the legends of figures 5B, C, D, F, G, H.

Response: We have revised all relevant figure legends accordingly, including the definitions of statistical significance annotations, exact P values, statistical tests, box plot components, sample sizes (n), and error bars, as requested (Page 36-37, Line 1283-1286, 1292-1296, 1302, 1307-1308, 1309-1311, 1315-1319, 1321 and 1324-1328).

8. Sections need to be named and the order should be corrected: Title page - Abstract - Introduction - Results - Discussion -Methods - Data Availability - Acknowledgements - Disclosure and Competing Interests Statement - References - Figure Legends - Table(s) - Expanded View Figure Legends.

Response: We have reorganized the manuscript accordingly. The section headings and their order have been revised to comply with the journal's requirements.

7th Jul 2026
Manuscript number: MSB-2026-13561RR
Title: Deciphering global transcriptional dynamics coordinated by gene-gene regulatory interactions using single-cell data

Dear Prof Zhang,

Thank you again for sending us your revised manuscript. We are now satisfied with the modifications made and I am pleased to inform you that your paper has been accepted for publication.

Your manuscript will be processed for publication by EMBO Press. It will be copy edited and you will receive page proofs prior to publication. Please note that you will be contacted by Springer Nature Author Services to complete licensing and payment information.

You may qualify for financial assistance for your publication charges - either via a Springer Nature fully open access agreement or an EMBO initiative. Check your eligibility: https://link.springer.com/journal/44320/how-to-publish-with-us

Should you be planning a Press Release on your article, please notify the production team via this form in order to coordinate publication and release dates: https://forms.cloud.microsoft/e/eH4A8JVP6g

If you have any questions, please do not hesitate to contact the Editorial Office. Thank you for your contribution to Molecular Systems Biology.

Sincerely,
Jingyi

Jingyi Hou, PhD
Senior Editor
Molecular Systems Biology

>>> Please note that it is Molecular Systems Biology policy for the transcript of the editorial process (containing referee reports and your response letter) to be published as an online supplement to each paper. If you do NOT want this, you will need to inform the Editorial Office via email immediately. More information is available here: https://link.springer.com/partners/embo-press/editorial-policies#Peer%20review

EMBO Press Author Checklist

Corresponding Author Name: Jiajun Zhang	
Journal Submitted to: Molecular Systems Biology	
Manuscript Number: MSB-2026-13561	

USEFUL LINKS FOR COMPLETING THIS FORM
The EMBO Journal - Author Guidelines
EMBO Reports - Author Guidelines
Molecular Systems Biology - Author Guidelines
EMBO Molecular Medicine - Author Guidelines

Reporting Checklist for Life Science Articles (updated January
This checklist is adapted from Materials Design Analysis Reporting (MDAR) Checklist for Authors. MDAR establishes a minimum set of requirements in transparent reporting in the life sciences (see Statement of Task: 10.31222/osf.io/9sm4x). Please follow the journal's guidelines in preparing your
Please note that a copy of this checklist will be published alongside your article.

Abridged guidelines for figures
1. Data
The data shown in figures should satisfy the following conditions:
- the data were obtained and processed according to the field's best practice and are presented to reflect the results of the experiments in an accurate and unbiased manner.
- ideally, figure panels should include only measurements that are directly comparable to each other and obtained with the same assay.
- plots include clearly labeled error bars for independent experiments and sample sizes. Unless justified, error bars should not be shown for technical
- if n<5, the individual data points from each experiment should be plotted. Any statistical test employed should be justified.
- Source Data should be included to report the data underlying figures according to the guidelines set out in the authorship guidelines on Data

2. Captions
Each figure caption should contain the following information, for each panel where they are relevant:
- a specification of the experimental system investigated (eg cell line, species name).
- the assay(s) and method(s) used to carry out the reported observations and measurements.
- an explicit mention of the biological and chemical entity(ies) that are being measured.
- an explicit mention of the biological and chemical entity(ies) that are altered/varied/perturbed in a controlled manner.
- the exact sample size (n) for each experimental group/condition, given as a number, not a range;
- a description of the sample collection allowing the reader to understand whether the samples represent technical or biological replicates (including how many animals, litters, cultures, etc.).
- a statement of how many times the experiment shown was independently replicated in the laboratory.
- definitions of statistical methods and measures:
  - common tests, such as t-test (please specify whether paired vs. unpaired), simple χ2 tests, Wilcoxon and Mann-Whitney tests, can be unambiguously identified by name only, but more complex techniques should be described in the methods section;
  - are tests one-sided or two-sided?
  - are there adjustments for multiple comparisons?
  - exact statistical test results, e.g., P values = x but not P values < x;
  - definition of 'center values' as median or average;
  - definition of error bars as s.d. or s.e.m.

Please complete ALL of the questions below.
Select "Not Applicable" only when the requested information is not relevant for your study.

Materials

Newly Created Materials	Information included in the manuscript?	In which section is the information available? (Reagents and Tools Table, Materials and Methods, Figures, Data Availability Section)
New materials and reagents need to be available; do any restrictions apply?	Not Applicable	

Antibodies	Information included in the manuscript?	In which section is the information available? (Reagents and Tools Table, Materials and Methods, Figures, Data Availability Section)
For **antibodies** provide the following information:<br>- Commercial antibodies: RRID (if possible) or supplier name, catalogue number and or/clone number<br>- Non-commercial: RRID or citation	Not Applicable	

DNA and RNA sequences	Information included in the manuscript?	In which section is the information available? (Reagents and Tools Table, Materials and Methods, Figures, Data Availability Section)
**Short novel DNA or RNA including primers, probes**: provide the sequences.	Not Applicable	

Cell materials	Information included in the manuscript?	In which section is the information available? (Reagents and Tools Table, Materials and Methods, Figures, Data Availability Section)
**Cell lines:** Provide species information, strain. Provide accession number in repository **OR** supplier name, catalog number, clone number, and/**OR** RRID.	Not Applicable	
**Primary cultures:** Provide species, strain, sex of origin, genetic modification status.	Not Applicable	
Report if the cell lines were recently **authenticated** (e.g., by STR profiling) and tested for mycoplasma contamination.	Not Applicable	

Experimental animals	Information included in the manuscript?	In which section is the information available? (Reagents and Tools Table, Materials and Methods, Figures, Data Availability Section)
**Laboratory animals or Model organisms:** Provide species, strain, sex, age, genetic modification status. Provide accession number in repository **OR** supplier name, catalog number, clone number, **OR** RRID.	Not Applicable	
**Animal observed in or captured from the field:** Provide species, sex, and age where possible.	Not Applicable	
Please detail **housing and husbandry conditions**.	Not Applicable	

Plants and microbes	Information included in the manuscript?	In which section is the information available? (Reagents and Tools Table, Materials and Methods, Figures, Data Availability Section)
**Plants:** provide species and strain, ecotype and cultivar where relevant, unique accession number if available, and source (including location for collected wild specimens).	Not Applicable	
**Microbes:** provide species and strain, unique accession number if available, and source.	Not Applicable	

Human research participants	Information included in the manuscript?	In which section is the information available? (Reagents and Tools Table, Materials and Methods, Figures, Data Availability Section)
If collected and within the bounds of privacy constraints report on age, sex and gender or ethnicity for all study participants.	Not Applicable	

Core facilities	Information included in the manuscript?	In which section is the information available? (Reagents and Tools Table, Materials and Methods, Figures, Data Availability Section)
If your work benefited from core facilities, was their service mentioned in the acknowledgments section?	Yes	Acknowledgments

Design

Study protocol	Information included in the manuscript?	In which section is the information available? (Reagents and Tools Table, Materials and Methods, Figures, Data Availability Section)
If study protocol has been **pre-registered, provide DOI in the manuscript**. For clinical trials, provide the trial registration number **OR** cite DOI.	Not Applicable	
Report the **clinical trial registration number** (at ClinicalTrials.gov or equivalent), where applicable.	Not Applicable	

Laboratory protocol	Information included in the manuscript?	In which section is the information available? (Reagents and Tools Table, Materials and Methods, Figures, Data Availability Section)
Provide DOI OR other citation details if **external detailed step-by-step protocols** are available.	Not Applicable	

Experimental study design and statistics	Information included in the manuscript?	In which section is the information available? (Reagents and Tools Table, Materials and Methods, Figures, Data Availability Section)
Include a statement about **sample size** estimate even if no statistical methods were used.	Yes	Materials and Methods
Were any steps taken to minimize the effects of subjective bias when allocating animals/samples to treatment (e.g. **randomization procedure**)? If yes, have they been described?	Not Applicable	
Include a statement about **blinding** even if no blinding was done.	Not Applicable	
Describe **inclusion/exclusion criteria** if samples or animals were excluded from the analysis. Were the criteria pre-established?<br><br>If sample or data points were omitted from analysis, report if this was due to attrition or intentional exclusion and provide justification.	Not Applicable	
For every figure, are **statistical tests** justified as appropriate? Do the data meet the assumptions of the tests (e.g., normal distribution)? Describe any methods used to assess it. Is there an estimate of variation within each group of data? **Is** the variance similar between the groups that are being statistically compared?	Yes	Materials and Methods

Sample definition and in-laboratory replication	Information included in the manuscript?	In which section is the information available? (Reagents and Tools Table, Materials and Methods, Figures, Data Availability Section)
In the figure legends: state number of times the experiment was **replicated** in laboratory.	Not Applicable	
In the figure legends: define whether data describe **technical or biological replicates**.	Not Applicable	

Ethics

Ethics	Information included in the manuscript?	In which section is the information available? (Reagents and Tools Table, Materials and Methods, Figures, Data Availability Section)
Studies involving **human participants**: State details of **authority granting ethics approval** (IRB or equivalent committee(s), provide reference number for approval.	Not Applicable	
Studies involving **human participants**: Include a statement confirming that **informed consent** was obtained from all subjects and that the experiments conformed to the principles set out in the WMA Declaration of Helsinki and the Department of Health and Human Services Belmont Report.	Not Applicable	
Studies involving **human participants:** For publication of **patient photos**, include a statement confirming that consent to publish was obtained.	Not Applicable	
Studies involving experimental **animals**: State details of **authority granting ethics approval** (IRB or equivalent committee(s), provide reference number for approval. Include a statement of compliance with ethical regulations.	Not Applicable	
Studies involving **specimen and field samples:** State if relevant **permits** obtained, provide details of authority approving study; if none were required, explain why.	Not Applicable	

Dual Use Research of Concern (DURC)	Information included in the manuscript?	In which section is the information available? (Reagents and Tools Table, Materials and Methods, Figures, Data Availability Section)
Could your study fall under dual use research restrictions? Please check biosecurity documents and list of **select agents and toxins** (CDC): https://www.selectagents.gov/sat/list.htm	Not Applicable	
If you used a select agent, is the security level of the lab appropriate and reported in the manuscript?	Not Applicable	
If a study is subject to dual use research of concern regulations, is the name of the **authority granting approval and reference number** for the regulatory approval provided in the manuscript?	Not Applicable	

Reporting

The MDAR framework recommends adoption of discipline-specific guidelines, established and endorsed through community initiatives. Journals have their own policy about requiring specific guidelines and recommendations to complement MDAR.

Adherence to community standards	Information included in the manuscript?	In which section is the information available? (Reagents and Tools Table, Materials and Methods, Figures, Data Availability Section)
State if relevant guidelines or checklists (e.g., **ICMJE, MIBBI, ARRIVE, PRISMA**) have been followed or provided.	Not Applicable	
For **tumor marker prognostic studies,** we recommend that you follow the **REMARK** reporting guidelines (see link list at top right). See author guidelines, under 'Reporting Guidelines'. Please confirm you have followed these guidelines.	Not Applicable	
For **phase II and III randomized controlled trials**, please refer to the **CONSORT** flow diagram (see link list at top right) and submit the CONSORT checklist (see link list at top right) with your submission. See author guidelines, under 'Reporting Guidelines'. Please confirm you have submitted this list.	Not Applicable	

Data Availability

Data availability	Information included in the manuscript?	In which section is the information available? (Reagents and Tools Table, Materials and Methods, Figures, Data Availability Section)
Have **primary datasets** been deposited according to the journal's guidelines (see 'Data Deposition' section) and the respective accession numbers provided in the Data Availability Section?	Yes	Data Availability
Were **human clinical and genomic datasets** deposited in a public access-controlled repository in accordance to ethical obligations to the patients and to the applicable consent agreement?	Not Applicable	
Are **computational models** that are central and integral to a study available without restrictions in a machine-readable form? Were the relevant accession numbers or links provided?	Yes	Code Availability
If publicly available data were reused, provide the respective **data citations in the reference list**.	Yes	Data Availability, References
